# Allyl methyl trisulfide protected against LPS-induced acute lung injury in mice via inhibition of the NF-κB and MAPK pathways

**DOI:** 10.3389/fphar.2022.919898

**Published:** 2022-08-08

**Authors:** Shuo Wang, Jinqian Liu, Jing Dong, Zongqiang Fan, Fugui Wang, Ping Wu, Xiaojing Li, Ruirui Kou, Fang Chen

**Affiliations:** ^1^ School of Pharmaceutical Sciences, Liaocheng University, Liaocheng, Shandong, China; ^2^ School of Public Health, Shandong University, Jinan, Shandong, China

**Keywords:** acute lung injury, allyl methyl trisulfide, inflammatory mediators, NF-κB, mapks

## Abstract

Allyl methyl trisulfide (AMTS) is one major lipid-soluble organosulfur compound of garlic. Previous studies have reported the potential therapeutic effect of garlic on acute lung injury (ALI) or its severe condition acute respiratory distress syndrome (ARDS), but the specific substances that exert the regulatory effects are still unclear. In this study, we investigate the protective effects of AMTS on lipopolysaccharide (LPS)-induced ALI mice and explored the underlying mechanisms. In *vivo* experiments, ICR mice were pretreated with 25–100 mg/kg AMTS for 7 days and followed by intratracheal instillation of LPS (1.5 mg/kg). The results showed that AMTS significantly attenuated LPS-induced deterioration of lung pathology, demonstrated by ameliorative edema and protein leakage, and improved pulmonary histopathological morphology. Meanwhile, the expression of inflammatory mediators and the infiltration of inflammation-regulation cells induced by LPS were also inhibited. *In vitro* experiments also revealed that AMTS could alleviate inflammation response and inhibit the exaggeration of macrophage M1 polarization in LPS-induced RAW264.7 cells. Mechanistically, we identified that AMTS treatment could attenuate the LPS-induced elevation of protein expression of p-IκBα, nuclear NF-κB-p65, COX2, iNOS, p-P38, p-ERK1/2, and p-JNK. Collectively, these data suggest that AMTS could attenuate LPS-induced ALI and the molecular mechanisms should be related to the suppression of the NF-κB and MAPKs pathways.

## Introduction

Acute lung injury (ALI) and its severe condition, acute respiratory distress syndrome (ARDS), are common causes of morbidity and mortality in critically ill patients (Quan et al., 2021). It arises from certain insults, including pulmonary (e.g., pneumonia, aspiration) or nonpulmonary conditions (e.g., sepsis, trauma), and is characterized by severe hypoxemia and pulmonary edema. The SARS-CoV-2/COVID-19 pandemic has inspired global interest in the pathophysiological pathways of ARDS (Hariri and Hardin, 2020). However, its pathogenesis remains unclear. And until now, there is no specific and effective pharmacologic strategy for the treatment of ALI/ARDS, which highlights the urgent need to develop therapeutic strategies (Fan et al., 2018).

During the processes of ALI, the balance of pro- and anti-inflammatory cytokines play an important role in the mediating, amplifying, and perpetuating stage (Goodman et al., 2003). The production of inflammatory cytokines is regulated by multiple signal pathways, among which, the mitogen-activated protein kinase (MAPK) and nuclear factor kappa B (NF-κB) pathways are believed to be particularly relevant to the pathophysiology of ALI (Shanley and Wong, 2001). Microbial components (pathogen-associated molecular pattern molecules [PAMPs] such as lipopolysaccharide [LPS]) or tissue injury–associated endogenous molecules (danger-associated molecular patterns [DAMPs]) bind to the cellular pattern recognition receptors (PRRs; e.g. Toll-like receptors [TLRs]) (Opitz et al., 2010). And then, PRR signaling leads to an inappropriate pro-inflammatory cytokine (e.g. interleukin 6 [IL-6], IL-1β, and tumor necrosis factor [TNF-a]) and chemokines (e.g. monocyte chemotactic protein 1 [MCP-1]) release via the MAPK and NF-κB pathways. Therefore, suppression of inflammatory responses by blocking the activation of the MAPK and NF-κB could be an effective strategy to minimize the clinical severity and mortality of ALI and ARDS.

Garlic (*Allium sativum L.*) has been widely used for centuries as spice and medicinal ingredient (El-Saber Batiha et al., 2020). Its pharmacological activities, such as antimicrobial, antioxidant, anticancer, antidiabetic, and anti-inflammatory activities, have been corroborated by researchers (Rana et al., 2011). The various biological properties exhibited by garlic are highly related to the various organosulfur compounds (OSCs) produced by its metabolic transformation (Trio et al., 2014). Allyl methyl trisulfide (AMTS) is one major sulfide of garlic oil, obtained by steam distillation (Yan et al., 1992). Previous studies have demonstrated the protective effect of other two major sulfides of garlic oil, diallyl disulfide (DADS) and diallyl trisulfide (DATS) on lung disease (Zhang et al., 2015; Kumar and Tamizhselvi, 2020). However, as a subtle change in the structure of compounds might strictly affect their potentials *in vivo* (Hosono-Fukao et al., 2009), whether AMTS could protect against lung damage remains unclear. The present study was designed to investigate the protective effect of AMTS in LPS-induced ALI and explore the underlying mechanisms *in vivo* and *in vitro* with a focus on the inflammatory signaling pathways.

## Materials and methods

### Chemicals and reagents

LPS (strain O111:B4) was purchased from Sigma-Aldrich. AMTS was provided by Hubei Xinkang Pharmaceutical Chemical Co., Ltd (Tianmen China). Mouse monoclonal anti-F4/80 and anti-CD68 were purchased from Santa Cruz Biotechnology. Primary antibodies against cytoplasm inhibitor of kappa B alpha (IKBα), p-IKBα, NF-κB p65, p-p65, iNOS, COX2, JNK, phospho-JNK, p38, phospho-p38, Erk1/2, phospho-Erk1/2 were provided by Cell Signaling Technology Inc (Beverly, MA, United States). Mouse monoclonal β-actin and lamin B antibodies were purchased from Proteintech (Wuhan, China). Mouse TNF-α, IL-1β, IL-6, and MCP-1 enzyme-linked immunosorbent assay (ELISA) kits were purchased from R&D (Minneapolis, MN, United States). Myeloperoxidase (MPO) and nitric oxide (NO) commercial assay kits were bought from JianCheng Bio Co., Ltd (Nanjing, China). All other chemicals were of the highest quality commercially available.

### Animal treatment

Male ICR Mice (6–8 weeks, 18–22 g) were obtained from Jinan Pengyue Laboratory Animal Breeding Co. Ltd (Jinan, China) and maintained in standard laboratory conditions (20–22 °C, 50%–55% humidity, 12 h light/dark cycle) with free access to diet and drinking water. All the procedures were approved by the Ethics Committee of Liaocheng University. After 1 week of acclimation, sixty animals were randomly divided into five groups (*n* = 12 per group), i.e. Control group, ALI group, and three ALI + AMTS groups. Mice in the ALI + AMTS groups were pretreated with 25, 50, 100 mg/kg AMTS (o.p.) for 7 days, respectively, while mice in the control group and ALI group were treated with the same volume of the vehicle (corn oil). The doses of AMTS selected were based on previous reports in the literature (Zhao et al., 2019). On day 8, mice were anesthetized with 4% isoflurane and animals in the ALI group and ALI/AMTS groups were intratracheally instilled with LPS (1.5 mg/kg, 50 µL per animal), while animals in the control group were treated with the same volume of the vehicle (sterile saline). The mice were anesthetized and sacrificed 6 h after saline or LPS treated and samples were collected. In each group, six animals were used to collect the bronchoalveolar lavage fluid (BALF). For the other six mice, after recording their wet weight, the left lung was collected for western blot, MPO activity, and NO assay. The right lung was fixed with 4% paraformaldehyde (PFA) for histopathological study.

### Cell culture and preparation

Mouse Raw264.7 macrophage cell line (ATCC, MD, America) was used for *in vitro* experiment. The cells were cultured in 10% FBS-DMEM medium and maintained at 37 °C with 5% CO_2_. The dose of AMTS was chosen based on cell cytotoxic assays using CCK-8 kits (Dojindo Laboratorise, Japan). In the LPS-driven inflammatory experiment, the macrophages were stimulated with 1 μg/ml LPS and treated with or without 30 μM AMTS for 24 h. The culture medium and cells were harvested for subsequent experiments.

### Histopathological examination

Paraffin sections (5 μm) of the lung were prepared with the PFA-fixed tissue and stained with hematoxylin and eosin (H&E) following a standard protocol. The histopathological changes of each mouse were viewed and representative photographs were captured by using an Olympus BX53 microscope. The lung injury was scored according to the method described by An *et al* (An et al., 2020). Briefly, Lung injury scoring criteria of ALI were as following: alveolar and interstitial hemorrhage, pulmonary edema, alveolar or interstitial inflammatory cells infiltration or aggregation, and thickness of alveolar wall/hyaline membrane formation. The severity of damage scored from 0–4 with 0 for no damage and four for the maximum damage.

### Enzyme-linked immunosorbent assay (ELISA)

ELISA was used to measure the protein level of inflammatory cytokines (e.g., TNF-α, IL-1β, IL-6) in BALF and cell culture medium, together with MCP-1 in lung tissue homogenate, according to the manufacturer’s protocols. Optic density was measured using a SpectraMax^®^ iD3 microplate reader (Molecular Devices, CA, United States) at 450 nm. The concentrations of cytokines were calculated according to the standard curve and expressed as pg/mL.

### MPO and NO assay

The MPO activity in lung tissue homogenate was measured according to the manufacturer of commercial kits (JianCheng Bio, Nanjing, China). The production of NO in cell culture medium and lung tissue homogenate was measured as the amount of nitrite with the Griess method. The amount of MPO and NO was expressed as U/mg protein and nmol/mg protein, respectively.

### Immunohistochemistry and immunofluorescence

Paraffin sections (5 µm) of the lung were used to perform the immunohistochemical assay. After deparaffinization and rehydration, endogenous peroxidase activity and nonspecific staining were blocked with 3% H_2_O_2_ and 5% sheep serum, respectively. Then the sections were incubated with F4/80 or CD68 primary antibody at 4 °C overnight. Afterward, sections were incubated with poly peroxidase-anti-mouse IgG antibody for 20 min at room temperature and counterstained with hematoxylin for 2 min. The sections were viewed and photographed under a light microscope. The percentage of the immunolabeled area within the total area were measured by using ImageJ software as previous described (Li et al., 2019).

For immunofluorescence, fixed and permeabilized RAW264.7 were incubated with mouse anti-F4/80 antibody and rabbit anti-iNOS antibody followed by an Alexa Fluor 488-labeled goat anti-mouse IgG antibody (Jackson ImmunoResearch) and by an Alexa Fluor 594-labeled goat anti-rabbit IgG antibody (Jackson ImmunoResearch), respectively. The nuclei were stained by Hochest 33342 (Thermo Fisher). All sections were photographed in five random visual fields. The F4/80^+^ positive cells represented the total macrophages and the F4/80^+^iNOS^+^ represented the M1 phenotype macrophages.

### Western blot analysis

The total protein of lung tissues and cultured cells was extracted by using RIPA buffer (P0013D, Beyotime, Shanghai, China) containing 1% protease and phosphatase inhibitor cocktail. The homogenates were centrifuged at 12,000 g for 20 min and the supernatants were collected as the total protein. The cytosolic and nuclear protein fractions of mice lungs were prepared according to the instruction of a commercial Nuclear and Cytoplasmic Protein Extraction Kit (Sangon Biotech, Shanghai, China). The protein concentration of the prepared samples was determined by using BCA protein assay kits. Equal aliquots of samples were mixed with loading buffer, and heated at 100°C for 5 min.

An appropriate amount of protein samples (10–40 μg) was separated by 7.5–15% SDS/polyacrylamide gel electrophoresis and then transferred to PVDF membranes. After blocking for 1 h with 4% skim milk in TBST buffer, the membranes were incubated with primary antibodies overnight at 4°C. Then the membranes were washed in TBST and incubated with horseradish peroxidase-conjugated secondary antibody for 1 h at room temperature. Immunoreactive bands of protein were detected by an enhanced chemiluminescence kit and exposed to X-ray films. The blots were scanned using an EPSON DS-6500 scanner, and the relative optical density of the bands was quantified by using ImageJ software.

### Statistical analysis

SPSS version 16.0 (SPSS, Chicago, IL, United States) and GraphPad Prism 8 (La Jolla, CA, United States) were used for statistical analysis. The data were expressed as the mean ± SD and were analyzed by one-way ANOVA, followed by Tukey’s test. Differences were considered significant when *p*-values were less than 0.05.

## Results

### The effects of AMTS on LPS-induced ALI

**FIGURE 1:**
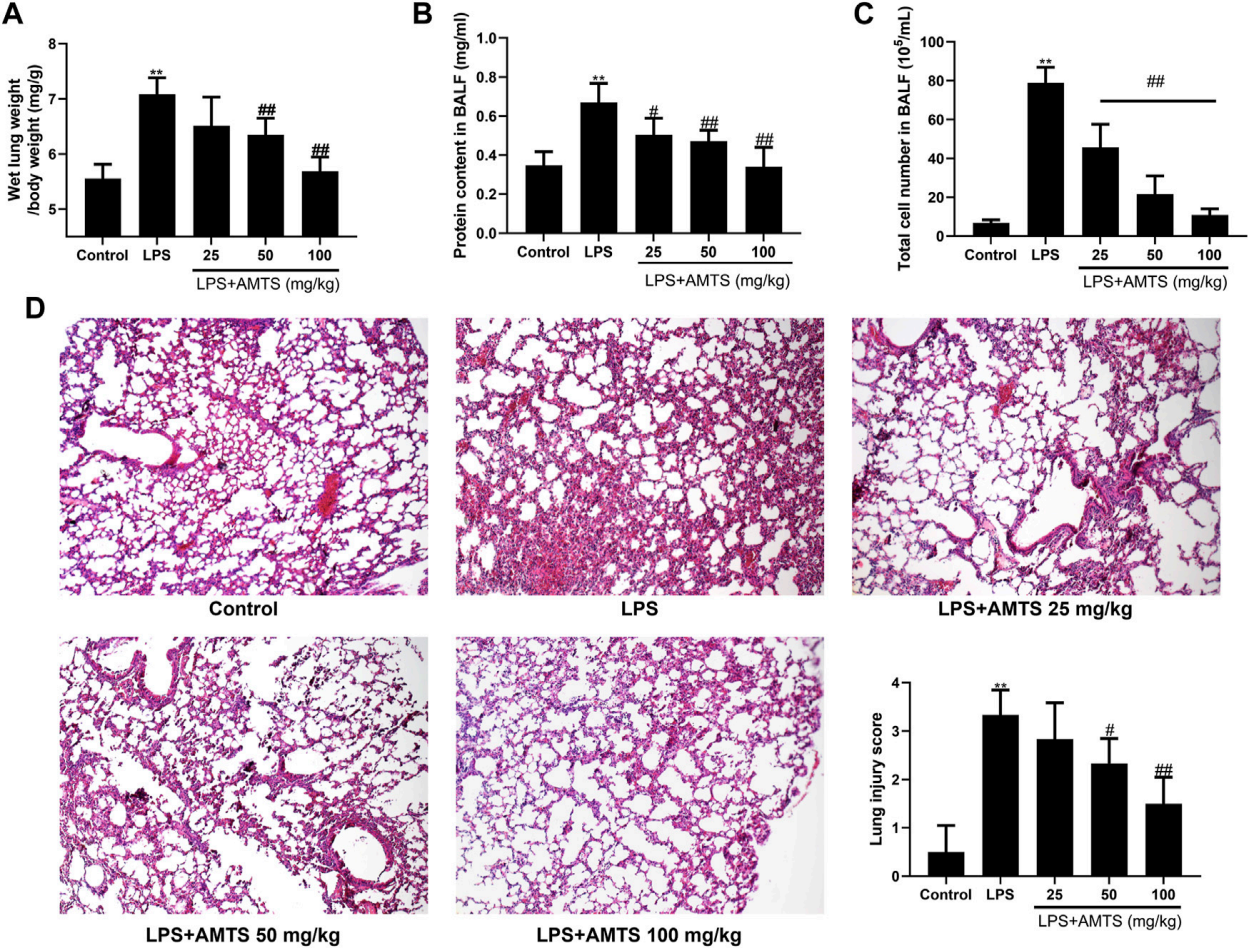
AMTS pretreatment attenuated LPS-induced ALI in mice. **(A)** Wet lung weight is normalized by body weight. Protein concentration **(B)** and total cells **(C)** in BALF. **(D)** Representative H and E staining (100×) and lung injury score for histological examination. Data were shown as mean ± S. D (n = 6). *p < 0.05, **p < 0.01, compared with the control group; ^#^ *p* < 0.05, ^##^ *p* < 0.01, compared with the LPS group.

The increase of wet lung weight and protein leakage usually indicated the permeability of alveolar-capillary members and pulmonary edema, which are critical features of ALI (Thompson et al., 2017). As shown in Figures 1A,B, LPS installation significantly increased the wet lung weight (*p* < 0.01) and protein leakage in BALF (*p* < 0.01). However, 50 mg/kg and 100 mg/kg AMTS administration remarkably reduced the lung weight increase (*p* < 0.01). Additionally, the protein accumulation was also remarkably reduced in all AMTS co-treated groups (*p* < 0.05). Another major central feature of ALI is inflammatory cell infiltration. Compared with the control group, LPS treatment significantly increased the number of total cells in BALF (*p* < 0.01, Figure 1C). The increased cell number in BALF was dose-dependently reduced after AMTS administration (*p* < 0.01). The histological examination further demonstrated AMTS pretreated suppressed LPS induced ALI (Figure 1D). Compared with the sections of control group mice, lung sections of mice in the LPS group expressed extensive morphological changes, such as infiltration of inflammatory cells into the alveolar space, parenchyma, hemorrhage, and alveolar wall thickening. Again, the severity of histopathological change and lung injury scores were ameliorated by AMTS.

### AMTS administration inhibited LPS-induced release of inflammatory mediators

**FIGURE 2:**
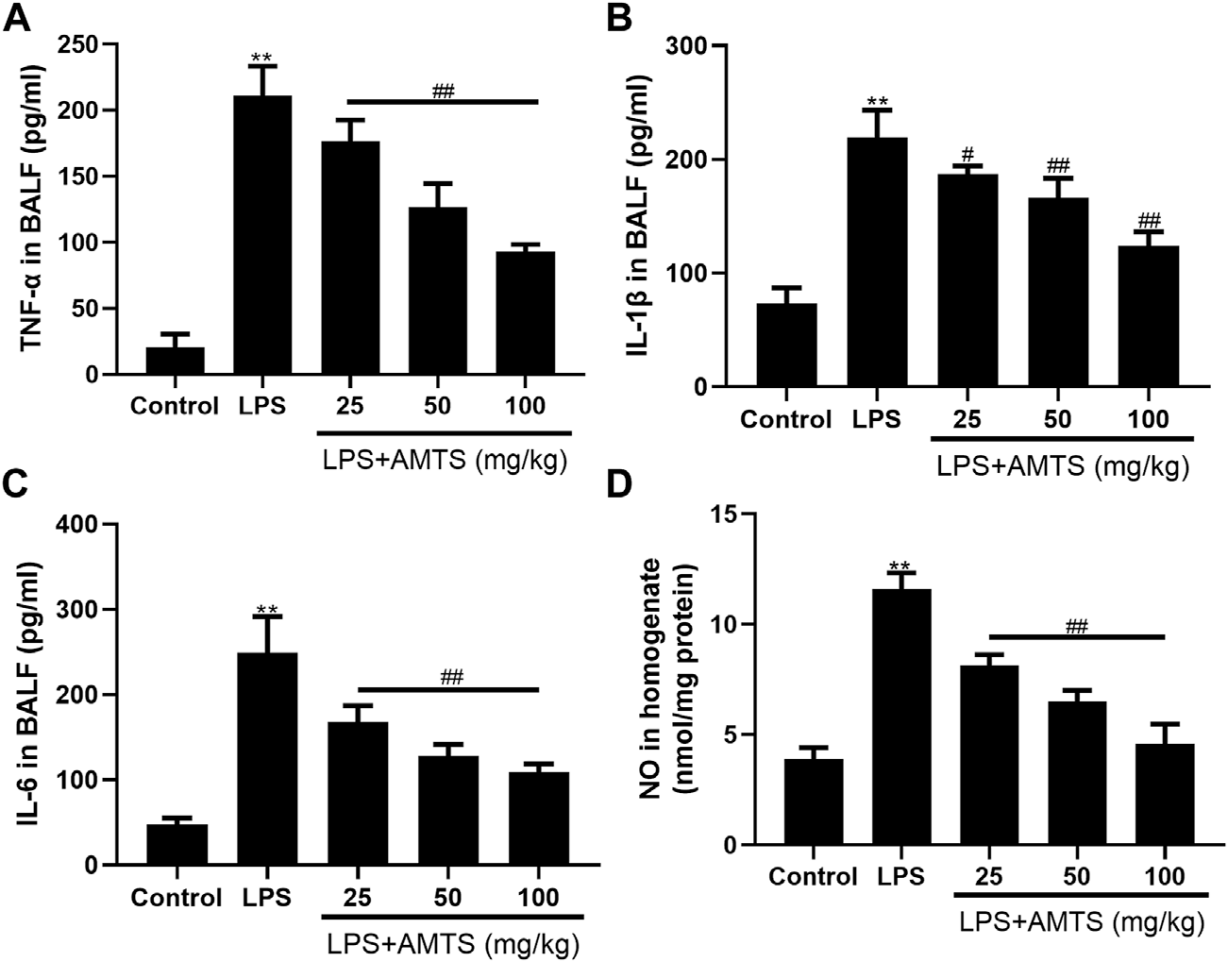
Effects of AMTS on inflammatory mediators. **(A)**TNF-α **(B)** IL-1β, **(C)** IL-6 in BALF. **(D)** NO in lung tissue. Data were presented as mean ± S. D (n = 6). *p < 0.05, **p < 0.01, compared with the control group; ^#^ *p* < 0.05, ^##^ *p* < 0.01, compared with the LPS group.

It has been well documented that the expression of inflammatory mediators is associated with the outcome of lung injury. Therefore, we investigated the protein levels of pro-inflammatory cytokines in BALF by ELISA. As shown in Figure 2, LPS administration significantly increased BALF levels of TNF-α, IL-1β, and IL-6 (*p* < 0.01). In contrast, compared with the ALI group, pretreatment with different dosages of AMTS dose-dependently decreased the levels of TNF-α, IL-1β, and IL-6 (*p* < 0.05). Interestingly, the level of NO in lung tissue, another key inflammatory mediator, also significantly increased compared with that of the control group (*p* < 0.01), whereas the AMTS intervention notably reduced its level by 29.9, 44.1, and 60.6%, respectively, compared with that of the LPS group (*p* < 0.01).

### AMTS inhibited the infiltration of inflammation-regulation cells in ALI

**FIGURE 3:**
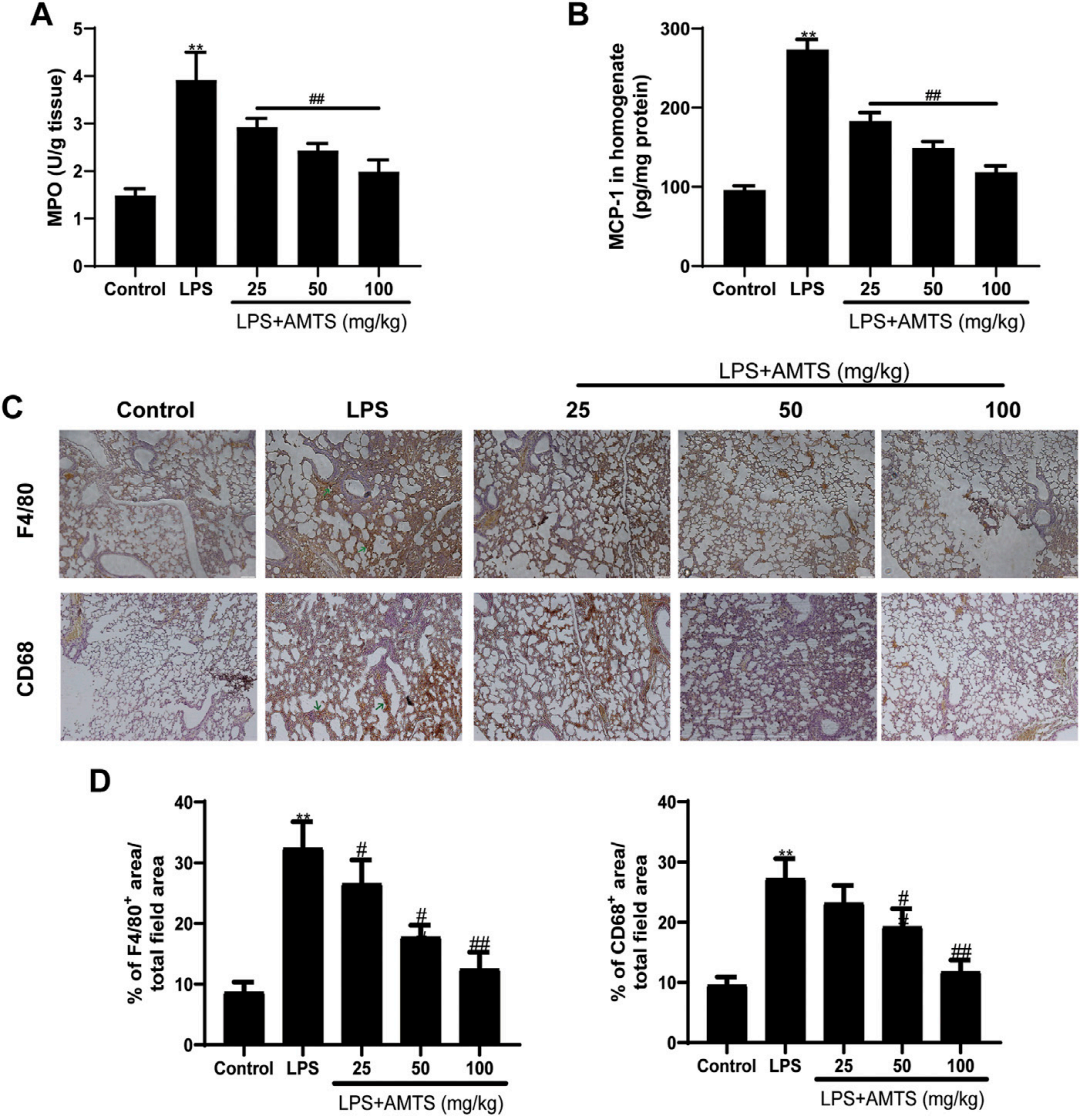
Effects of AMTS on the infiltration of inflammation-regulation cells. **(A)** The activity of MPO was determined to indicate the infiltration of neutrophils. **(B)** AMTS pretreatment reduced MCP-1, an inflammatory cell recruitment chemokine, which was measured by ELISA. **(C)** Representative pictures of immune stain for macrophage using F4/80 and CD11 b antibody. The green arrows indicate the infiltration of inflammatory cells. **(D)**The percentage of positive staining area to the total area for F4/80 and CD68 in lung tissues. *p < 0.05, **p < 0.01, compared with the control group; ^#^ *p* < 0.05, ^##^ *p* < 0.01, compared with the LPS group.

Leukocytes infiltration is another physiologic characterization of ALI. The activity of MPO is widely used to assess the infiltration of neutrophils in tissue (Khan et al., 2018). As shown in Figure 3A, compared with the control group, LPS treatment significantly increased the activity of MPO in the lung tissue (*p* < 0.01), while pretreatment of AMTS significantly reversed the rise of it (*p* < 0.01). Besides, we detected the level of chemokines as an indirect measurement of inflammatory cell recruitment to the lung. LPS treatment significantly increased the expression of MCP-1, and the increase was significantly reversed by different dose treatments of AMTS (*p* < 0.01, Figure 3B). Beyond that, we also evaluated the infiltration of macrophages by immunostaining using macrophage-specific anti-F4/80 and anti-CD68 antibodies. As shown in Figures 3C,D, F4/80^+^ and/or CD68^+^ cells in the sections of the LPS group increased significantly compared with the control group (*p* < 0.01), and AMTS administration led to a significant decline of F4/80^+^ and/or CD68^+^ cells (*p* < 0.05).

### AMTS repressed NF-κB activation in LPS-induced ALI

**FIGURE 4:**
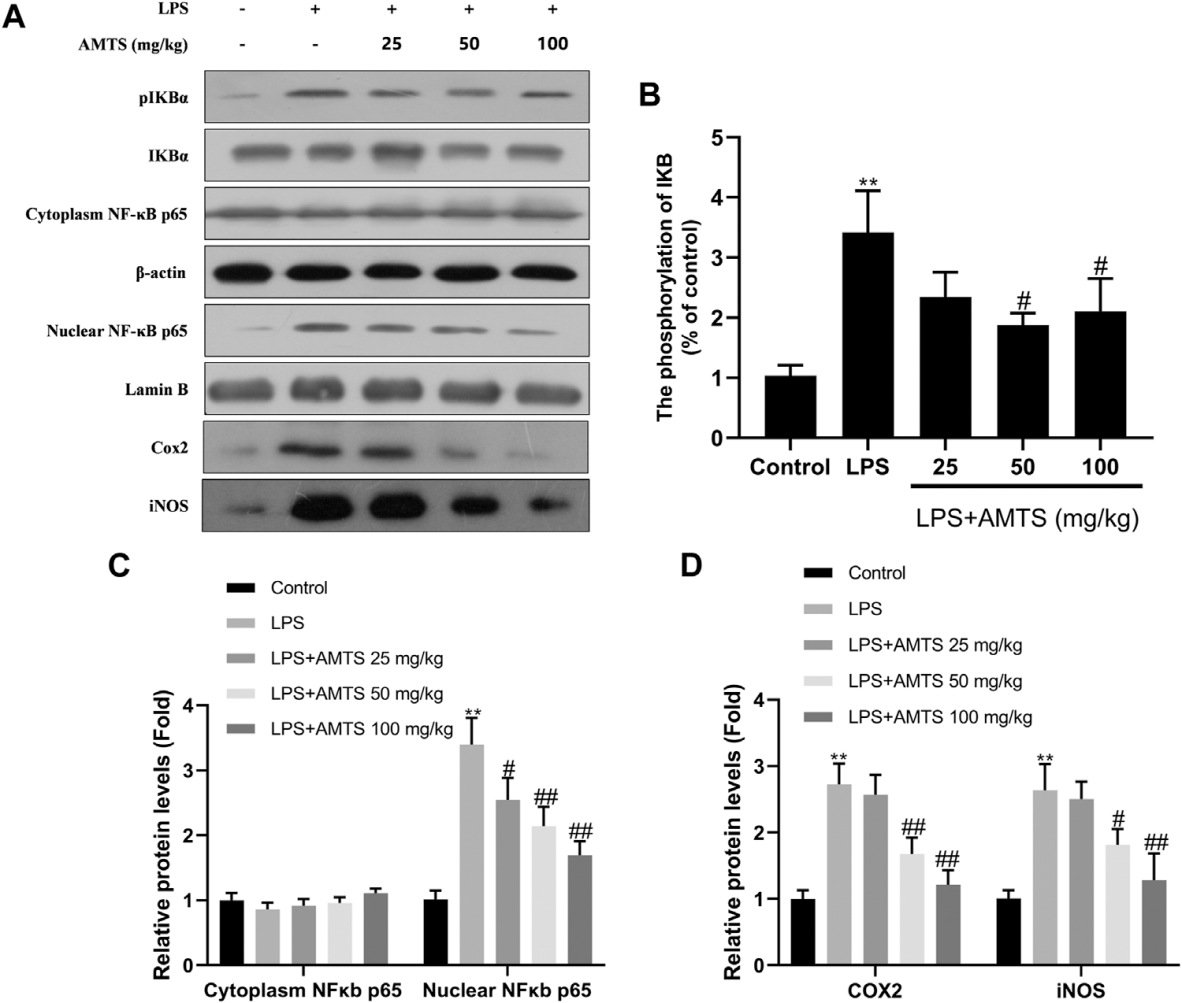
AMTS treatment inhibited the activation of the NF-κB pathway. **(A)** Representative western blot bands for IκBα, p-IκBα, cytoplasm and nuclear NF-κB p65; **(B)** quantitative data analyses of the phosphorylation levels of IκB; **(C)** the expression of cytoplasm and nuclear NFκB p65; **(D)** the expression of COX2 and iNOS. Data were presented as mean ± SD from at least three independent experiments and expressed as the percentage of the control. *p < 0.05, **p < 0.01, compared with control group; ^#^ *p* < 0.05, ^##^ *p* < 0.01, compared with LPS group.

Since the pro-inflammatory cytokines are targets of the NF-κB signaling pathway, the protein expression of IκBα, p-IκBα, NF-κB p65, and nuclear NF-κB p65 was examined by western blotting (Figure 4). As shown in the figure, the ratio of p-IκBα/IκBα was dramatically increased after LPS exposure, which was inhibited by 50 and 100 mg/kg AMTS (*p* < 0.05). Beyond that, the protein level of nuclear NF-κB p65 was increased in the LPS group compared with the control group (*p* < 0.01), while AMTS pretreatment normalized the level of NF-κB p65 in the cytoplasm and nucleus in a dose-dependent manner (*p* < 0.05). Furthermore, AMTS feeding also blocked the increase expression of NF-κB downstream protein COX2 and iNOS. These results suggested that AMTS could suppress LPS-induced NF-κB activation in lung.

### Effects of AMTS on the change of MAPK pathway in LPS-induced ALI

**FIGURE 5:**
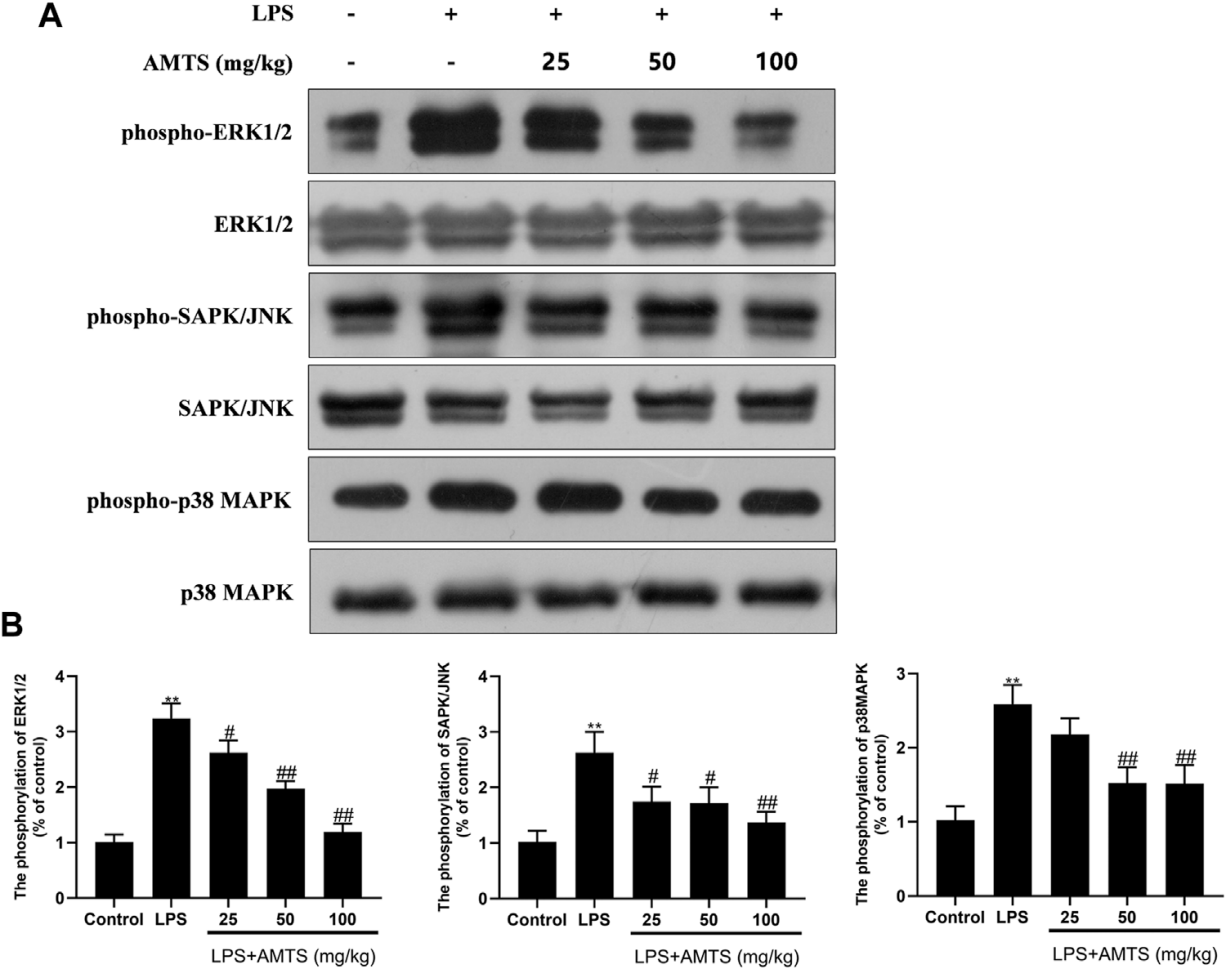
AMTS treatment inhibited the phosphorylation of Erk1/2, SAPK/NK, and p38MAPK. **(A)** Representative western blot bands for phospho-Erk1/2, Erk1/2, phospho-SAPK/JNK, SAPK/JNK, phospho-p38MAPK, and p38MAPK; **(B)** Quantitative data analyses. Data were presented as mean ± SD from at least three independent experiments and expressed as the percentage of the control. *p < 0.05, **p < 0.01, compared with control group; ^#^ *p* < 0.05, ^##^ *p* < 0.01, compared with LPS group.

In addition to NF-κB, pro-inflammatory cytokines could be also affected by MAPK. Therefore, the total and phosphorylated forms of three kinds of MAPK (ERK1/2, SAPK/JNK, and p38MAPK) were investigated in the lung tissue. As shown in Figure 5, the phosphorylation of MAPKs in the LPS group was significantly increased when compared with the control group (*p* < 0.01). Meanwhile, AMTS pretreatment significantly decreased the up-regulated phosphorylation of ERK1/2, SAPK/JNK, and p38 (*p* < 0.05).

### AMTS alleviated inflammation response and suppressed M1 polarization in LPS-stimulated RAW264.7 cells

**FIGURE 6:**
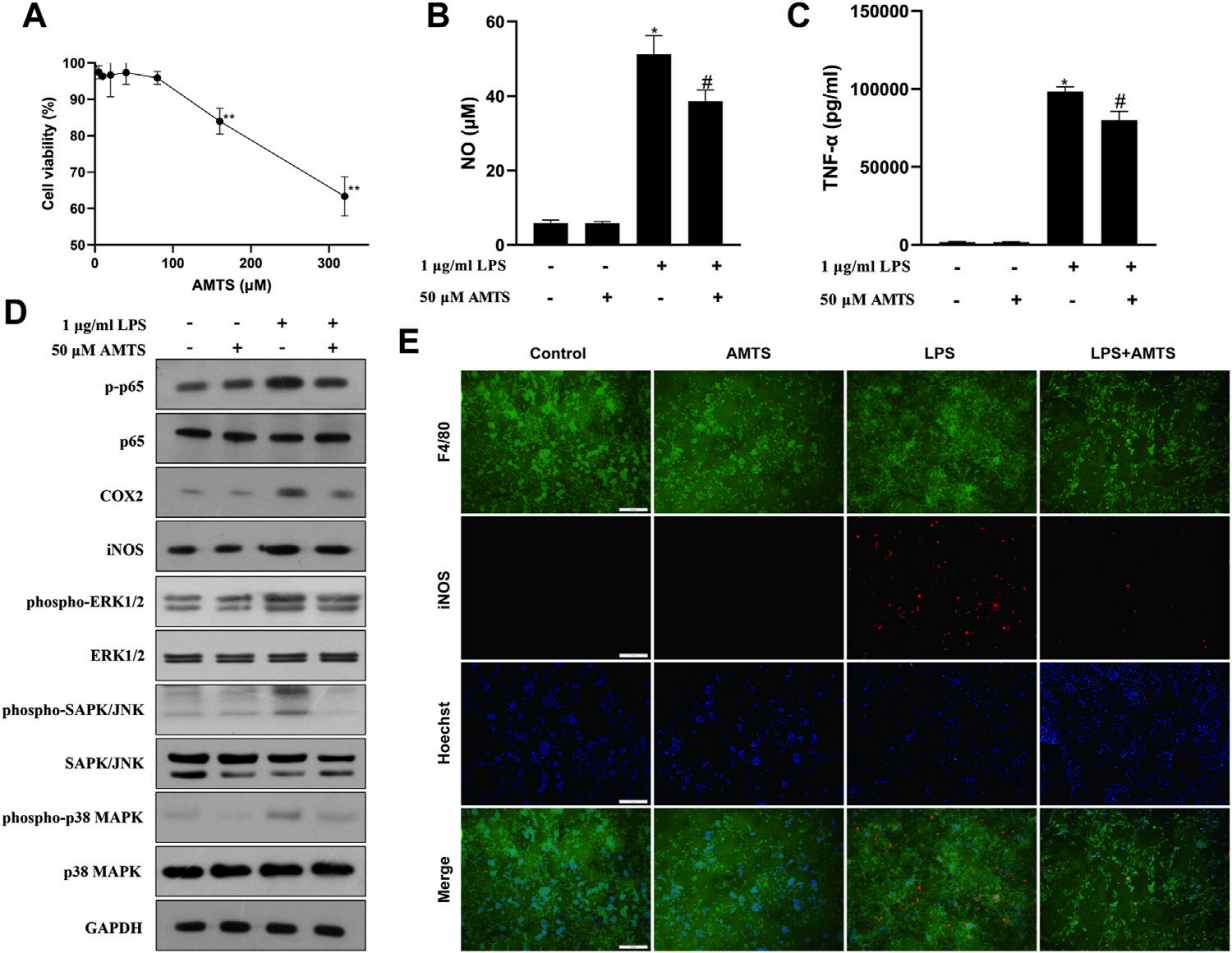
Effects of AMTS on LPS-induced inflammatory response in RAW264.7 cells. **(A)** The cytotoxicity of AMTS was assessed by CCK8 assay. The levels of NO **(B)** and TNF-α **(C)** in the culture medium were detected by ELISA. **(D)** Protein levels of the NF-κB pathway and the MAPKs pathway-related proteins were detected by western blotting. **(E)** Macrophage M1 polarization (F4/80^+^iNOS^+^) was detected by double immunofluorescence. *p < 0.05, **p < 0.01, compared with untreated cells (Control); ^#^ *p* < 0.05, ^##^ *p* < 0.01, compared with the cells challenged with LPS (LPS).

To verify the direct effect of AMTS on macrophages, LPS-stimulated RAW264.7 cells were co-treat with AMTS. According to the CCK8 assay, no cytotoxicity was found under the subsequently tested concentration (50 μM) on RAW264.7 cells (Figure 6A). AMTS decreased levels of NO and TNF-α in LPS-induced RAW264.7 cells (Figures 6B,C). Besides, AMTS decreased the phosphorylation of p65 and the expression of downstream iNOS and COX2 (Figure 6D). Moreover, AMTS prevented the increase of LPS-induced M1 polarized macrophages (F4/80^+^iNOS^+^, Figure 6E). These data suggested that AMTS could inhibit LPS inflammation, block the activation of the NFκB and the MAPKs signaling pathways, and suppress the macrophage M1 polarization in LPS-stimulated RAW264.7 cells.

## Discussion

The anti-inflammatory effect of garlic-derived organosulfur has been demonstrated in numerous *in vitro* and *in vivo* experiments (Arreola et al., 2015). With the pandemic outbreak of SARS-CoV-2 infection, reports on the protective effects of garlic organosulfur compounds for pulmonary disease have mushroomed (An et al., 2020; Mo et al., 2020). In the current study, we firstly demonstrated that AMTS, another garlic-derived organosulfur compound, could attenuate the intratracheal LPS induced ALI. Pretreatment with AMTS restrained the edema, protein leakage, and the histopathological changes of the lung, The overexpression of pro-inflammatory cytokines and chemokines, together with the infiltration of leukocytes were also suppressed. Additionally, LPS-induced NF-κB and MAPKs activation in pulmonary tissue was also found to be downregulated by the pretreatment of different doses of AMTS. The *in vitro* experiments substantiated the above results and further revealed that AMTS could modulate macrophages polarization. These finds provided solid evidence that AMTS could protect against LPS-induced ALI.

Previous studies have demonstrated that the derangement of cytokine drives ALI/ARDS pathology and disease progress (Deng and Standiford, 2010). These cytokines initiate the lung injury by direct cytotoxic or apoptotic effects to the alveolar-capillary membrane, or by promoting leukocyte activation and recruitment. TNF-α and IL-1β are of prominent importance in the context of ALI. They can stimulate the production of a host of other cytokines. Elevated levels of both IL-1β and TNF-α can be found in the BALF of patients with ARDS (Hyers et al., 1991; Pugin et al., 1996). In the current study, we also demonstrated that the expression of TNF-α, IL-1β, and IL-6 was significantly increased in BALF after LPS treatment. Furthermore, AMTS intervention significantly reduced the production of these pro-inflammatory cytokines in the BALF, accompanied by the alleviation of ALI pathological manifestations. These results indicated that the suppression of pro-inflammatory cytokines might attribute to the protective effect of AMTS against LPS-induced ALI.

As mentioned, pro-inflammatory mediators could also induce pulmonary damage by chemotaxis of leukocytes, such as macrophages and neutrophils. In the exudative phase of ALI, recruited macrophages activate and release excessive pro-inflammatory mediators to further promote lung damage (Huang et al., 2018). And the aggregate neutrophils produce numerous reactive oxygen species and protease that lead to epithelial injury and exacerbate the pro-inflammatory state (Potey et al., 2019). In the present study, LPS treatment induced the recruitment of macrophages and neutrophils. It was verified with routine histological markers of macrophage lineage cells (F4/80, a marker of mature macrophages and CD68, a marker of active macrophages) (Dambach et al., 2002) and the activity of MPO, respectively. Interestingly, oral administration of AMTS abrogated LPS-induced accumulation of leukocytes in the pulmonary tissue. Besides recruitment, the polarization of macrophages also plays an important role in the pathogenesis of ALI (Chen et al., 2020). Under different microenvironmental stimuli, macrophages can be divided into two main subtypes: the classically activated (M1) type and the alternatively activated (M2) type. M1 activated macrophages could exacerbate the release of pro-inflammatory mediators, thus promote the cascade amplification of inflammation, and therefore serve as facilitators in the process of lung tissue damage in ALI/ARDS (Lee et al., 2021). Modulating the polarization of lung macrophages is believed to be a promising method for the treatment of ALI and recent studies have revealed that some synthesized and naturally derived materials might exert their therapeutic effects by this way (Lin et al., 2020; Xie et al., 2021). The current *in vitro* study also directly revealed that AMTS had a negative effect on M1 polarization. Combined the above findings, it suggests that AMTS could inhibit the recruitment of leukocytes and modulate macrophage polarization to alleviate LPS-induced lung damage.

LPS-induced lung injury is one of the most practical and invariable animal models to explore and evaluate the efficient protective agents of ARDS/ALI (Domscheit et al., 2020; Chen et al., 2022). As a component of the outer membrane in Gram-negative bacteria, LPS could associate with LPS binding protein (LBP) and CD14, bind to the TLR4 and mediate its dimerization (Hu et al., 2013). The engagement of TLR4 homodimers interacts with myeloid differentiation factor 88 (MyD88), which could further facilitate the phosphorylation of IκB and MAPKs by other kinases. The phosphorylation of IκB causes the release and nuclear translocation of NF-κB and the phosphorylated MAPKs lead to the activation of adaptor protein-1 (AP-1). Then, nuclear translocation of NF-κB and AP-1 activates their target genes to promote the expression of various pro-inflammatory cytokines, such as TNF-α, IL-1β, IL-6, IL-8, and other inflammatory mediators, such as NO and prostaglandin. Therefore, the activation of the NF-κB and MAPK pathways could induce the overexpression of inflammatory cytokines, which leads to pathological changes of ALI (Zhang et al., 2017). Furthermore, the inflammatory cytokines, such as TNFα and IL-1β, could further promote the activation of the NF-κB and MAPKs, thereby performing “positive feedback” regulation to amplify the inflammatory response (Gaestel et al., 2009; Dorrington and Fraser, 2019). Besides, studies have also shown that the activation of the p38 MAPK signaling pathway can promote the phosphorylation and degradation of IκBα, which directly activates the NF-κB p65 pathway (Je et al., 2004). The interaction between these signaling pathways amplifies the inflammatory process and aggravates the pulmonary injury. Previous studies have demonstrated that NF-κB and MAPKs inhabitation by pharmacological activators could protect against LPS-induced ALI in mice, suggesting that NF-κB and/or MAPKs are potential therapeutic targets for ALI (Everhart et al., 2006; Zhou et al., 2019). In the current study, we found that pretreatment with AMTS suppressed the activation of NF-κB and MAPKs pathways induced by LPS. These results suggest that suppressed NF-κB and MAPKs pathway activation might contribute to the protective effects of AMTS against LPS-induced ALI.

Previous studies have revealed that some OSCs could exert their anti-inflammatory effect through the inhibition of the MAPK and NF-κB pathways, but their detailed mechanisms are still unclear (Rodrigues and Percival, 2019). A large amount of literature has indicated that the benefits of OSCs are associated with the number of sulfur atoms in their structure, which is closely related to its potential to increase endogenous hydrogen sulfide (H_2_S) (Benavides et al., 2007; Magli et al., 2021). H_2_S has been shown to play an important regulatory role in the inflammatory processes and inhibit the activation of NF-κB and MAPKs (Stuhlmeier et al., 2009; Wu et al., 2017). In addition to H_2_S, some studies demonstrated that DATS exerted inhibitory effects through the inhibition of their common upstream protein kinase and its antioxidant activities (You et al., 2013). Recently, another study suggested that DADS suppresses the activation of MAPK and NF-κB pathways through the activating nuclear factor erythroid 2-related factor 2 (Nrf2) (Zhang et al., 2022). Although research on the effects of OSCs has made some progress, there is a lack of research on the underlying mechanisms of AMTS. Therefore, further studies are needed to illustrate the mechanism of NF-κB and MAPKs pathway inhibition by AMTS.

## Conclusion

The results of the current study demonstrated that AMTS could significantly inhibit LPS-induced ALI demonstrated by ameliorative edema and protein leakage, improved pulmonary histopathological morphology, down expression of inflammatory mediators, and inhibition of inflammation-regulation cells infiltration. *In vitro* experiments also confirmed that AMTS could alleviate LPS-induced inflammation response and suppress M1 polarization of macrophages. Meanwhile, AMTS significantly attenuated the LPS-induced increase of the protein levels of p-IκBα, nuclear NF-κB-p65, COX2, iNOS, p-P38, p-ERK1/2, and p-JNK. These data suggest that the molecular mechanisms of AMTS against LPS-induced ALI should be related to the suppression of the NF-κB and MAPKs pathways in the lung tissue.

## Data Availability

The original contributions presented in the study are included in the article/Supplementary Material. Further inquiries can be directed to the corresponding authors.

## References

[B1] An L. Zhao J. Sun X. Zhou Y. Zhao Z. (2020). S-allylmercaptocysteine inhibits mucin overexpression and inflammation via mapks and pi3k-akt signaling pathways in acute respiratory distress syndrome. Pharmacol. Res. 159, 105032. 10.1016/j.phrs.2020.105032 32574825 PMC7305891

[B2] Arreola R. Quintero-Fabián S. López-Roa R. I. Flores-Gutiérrez E. O. Reyes-Grajeda J. P. Carrera-Quintanar L. (2015). Immunomodulation and anti-inflammatory effects of garlic compounds. J. Immunol. Res. 2015, 401630. 10.1155/2015/401630 25961060 PMC4417560

[B3] Benavides G. A. Squadrito G. L. Mills R. W. Patel H. D. Isbell T. S. Patel R. P. (2007). Hydrogen sulfide mediates the vasoactivity of garlic. Proc. Natl. Acad. Sci. U. S. A. 104, 17977–17982. 10.1073/pnas.0705710104 17951430 PMC2084282

[B4] Chen X. Tang J. Shuai W. Meng J. Feng J. Han Z. (2020). Macrophage polarization and its role in the pathogenesis of acute lung injury/acute respiratory distress syndrome. Inflamm. Res. 69, 883–895. 10.1007/s00011-020-01378-2 32647933 PMC7347666

[B5] Chen Y. Kuang Z. Wei W. Hu Y. Mu S. Ding H. (2022). Protective role of (5r)-5-hydroxytriptolide in lipopolysaccharide-induced acute lung injury by suppressing dendritic cell activation. Int. Immunopharmacol. 102, 108410. 10.1016/j.intimp.2021.108410 34865994

[B6] Dambach D. M. Watson L. M. Gray K. R. Durham S. K. Laskin D. L. (2002). Role of Ccr2 in macrophage migration into the liver during acetaminophen-induced hepatotoxicity in the mouse. Hepatology 35, 1093–1103. 10.1053/jhep.2002.33162 11981759

[B7] Deng J. C. Standiford T. J. (2010). Growth factors and cytokines in acute lung injury. Compr. Physiol. 1, 81–104. 10.1002/cphy.c090011 23737165

[B8] Domscheit H. Hegeman M. A. Carvalho N. Spieth P. M. (2020). Molecular dynamics of lipopolysaccharide-induced lung injury in rodents. Front. Physiol. 11, 36. 10.3389/fphys.2020.00036 32116752 PMC7012903

[B9] Dorrington M. G. Fraser I. D. (2019). Nf-κb signaling in macrophages: Dynamics, crosstalk, and signal integration. Front. Immunol. 10, 705. 10.3389/fimmu.2019.00705 31024544 PMC6465568

[B10] El-Saber Batiha G. Magdy Beshbishy A. G Wasef L. Elewa Y. H. A Al-Sagan A. El-Hack A. (2020). Chemical constituents and pharmacological activities of garlic (allium sativum L.): A review. Nutrients 12, 872. 10.3390/nu12030872 PMC7146530 32213941

[B11] Everhart M. B. Han W. Sherrill T. P. Arutiunov M. Polosukhin V. V. Burke J. R. (2006). Duration and intensity of NF-kappaB activity determine the severity of endotoxin-induced acute lung injury. J. Immunol. 176, 4995–5005. 10.4049/jimmunol.176.8.4995 16585596

[B12] Fan E. Brodie D. Slutsky A. S. (2018). Acute respiratory distress syndrome: Advances in diagnosis and treatment. Jama 319, 698–710. 10.1001/jama.2017.21907 29466596

[B13] Gaestel M. Kotlyarov A. Kracht M. (2009). Targeting innate immunity protein kinase signalling in inflammation. Nat. Rev. Drug Discov. 8, 480–499. 10.1038/nrd2829 19483709

[B14] Goodman R. B. Pugin J. Lee J. S. Matthay M. A. (2003). Cytokine-mediated inflammation in acute lung injury. Cytokine Growth Factor Rev. 14, 523–535. 10.1016/s1359-6101(03)00059-5 14563354

[B15] Hariri L. Hardin C. C. (2020). Covid-19, angiogenesis, and ards endotypes. N. Engl. J. Med. 383, 182–183. 10.1056/NEJMe2018629 32437597

[B16] Hosono-Fukao T. Hosono T. Seki T. Ariga T. (2009). Diallyl trisulfide protects rats from carbon tetrachloride-induced liver injury. J. Nutr. 139, 2252–2256. 10.3945/jn.109.109611 19812219

[B17] Hu R. Xu H. Jiang H. Zhang Y. Sun Y. (2013). The role of Tlr4 in the pathogenesis of indirect acute lung injury. Front. Biosci. 18, 1244–1255. 10.2741/4176 23747880

[B18] Huang X. Xiu H. Zhang S. Zhang G. (2018). The role of macrophages in the pathogenesis of ali/ards. Mediat. Inflamm. 2018, 1264913. 10.1155/2018/1264913 PMC5989173 29950923

[B19] Hyers T. M. Tricomi S. M. Dettenmeier P. A. Fowler A. A. (1991). Tumor necrosis factor levels in serum and bronchoalveolar lavage fluid of patients with the adult respiratory distress syndrome. Am. Rev. Respir. Dis. 144, 268–271. 10.1164/ajrccm/144.2.268 1859048

[B20] Je J. H. Lee J. Y. Jung K. J. Sung B. Go E. K. Yu B. P. (2004). NF-kappaB activation mechanism of 4-hydroxyhexenal via NIK/IKK and p38 MAPK pathway. FEBS Lett. 566, 183–189. 10.1016/j.febslet.2004.04.037 15147892

[B21] Khan A. A. Alsahli M. A. Rahmani A. H. (2018). Myeloperoxidase as an active disease biomarker: Recent biochemical and pathological perspectives. Med. Sci. 6, 33. 10.3390/medsci6020033 PMC6024665 29669993

[B22] Kumar M. M. Tamizhselvi R. (2020). Protective effect of diallyl disulfide against cerulein-induced acute pancreatitis and associated lung injury in mice - sciencedirect. Int. Immunopharmacol. 80, 106136. 10.1016/j.intimp.2019.106136 31991372

[B23] Lee J. W. Chun W. Lee H. J. Min J. H. Kim S. M. Seo J. Y. (2021). The role of macrophages in the development of acute and chronic inflammatory lung diseases. Cells 10, 897. 10.3390/cells10040897 33919784 PMC8070705

[B24] Li M. Wang S. Li X. Wang Q. Liu Z. Yu T. (2019). Inhibitory effects of diallyl sulfide on the activation of kupffer cell in lipopolysaccharide/D-galactosamine-induced acute liver injury in mice. J. Funct. foods 62, 103550. 10.1016/j.jff.2019.103550

[B25] Lin F. Song C. Zeng Y. Li Y. Li H. Liu B. (2020). Canagliflozin alleviates lps-induced acute lung injury by modulating alveolar macrophage polarization. Int. Immunopharmacol. 88, 106969. 10.1016/j.intimp.2020.106969 33182027

[B26] Magli E. Perissutti E. Santagada V. Caliendo G. Corvino A. Esposito G. (2021). H2s donors and their use in medicinal chemistry. Biomolecules 11, 1899. 10.3390/biom11121899 34944543 PMC8699746

[B27] Mo M. Li S. Dong Z. Li C. Sun Y. Li A. (2020). S-allylmercaptocysteine ameliorates lipopolysaccharide-induced acute lung injury in mice by inhibiting inflammation and oxidative stress via nuclear factor kappa B and keap1/nrf2 pathways. Int. Immunopharmacol. 81, 106273. 10.1016/j.intimp.2020.106273 32070920

[B28] Opitz B. Van Laak V. Eitel J. Suttorp N. (2010). Innate immune recognition in infectious and noninfectious diseases of the lung. Am. J. Respir. Crit. Care Med. 181, 1294–1309. 10.1164/rccm.200909-1427SO 20167850

[B29] Potey P. M. Rossi A. G. Lucas C. D. Dorward D. A. (2019). Neutrophils in the initiation and resolution of acute pulmonary inflammation: Understanding biological function and therapeutic potential. J. Pathol. 247, 672–685. 10.1002/path.5221 30570146 PMC6492013

[B30] Pugin J. Ricou B. Steinberg K. P. Suter P. M. Martin T. R. (1996). Proinflammatory activity in bronchoalveolar lavage fluids from patients with ards, a prominent role for interleukin-1. Am. J. Respir. Crit. Care Med. 153, 1850–1856. 10.1164/ajrccm.153.6.8665045 8665045

[B31] Quan C. Wang M. Chen H. Zhang H. (2021). Extracellular vesicles in acute respiratory distress syndrome: Recent developments from bench to bedside. Int. Immunopharmacol. 100, 108118. 10.1016/j.intimp.2021.108118 34492532

[B32] Rana S. Pal R. Vaiphei K. Sharma S. K. Ola R. (2011). Garlic in health and disease. Nutr. Res. Rev. 24, 60–71. 10.1017/S0954422410000338 24725925

[B33] Rodrigues C. Percival S. S. (2019). Immunomodulatory effects of glutathione, garlic derivatives, and hydrogen sulfide. Nutrients 11, 295. 10.3390/nu11020295 PMC6412746 30704060

[B34] Shanley T. P. Wong H. R. (2001). “Signal transduction pathways in acute lung injury: nf-?b and ap-1,” in Molecular Biology of Acute Lung Injury (Berlin, Germany: Springer), 1–16.

[B35] Stuhlmeier K. M. Bröll J. Iliev B. (2009). Nf-kappab independent activation of a series of proinflammatory genes by hydrogen sulfide. Exp. Biol. Med. 234, 1327–1338. 10.3181/0904-RM-137 19855074

[B36] Thompson B. T. Chambers R. C. Liu K. D. (2017). Acute respiratory distress syndrome. N. Engl. J. Med. 377, 1904–1905. 10.1056/NEJMc1711824 29117492

[B37] Trio P. Z. You S. He X. He J. Sakao K. Hou D.-X. (2014). Chemopreventive functions and molecular mechanisms of garlic organosulfur compounds. Food Funct. 5, 833–844. 10.1039/c3fo60479a 24664286

[B38] Wu D. Luo N. Wang L. Zhao Z. Bu H. Xu G. (2017). Hydrogen sulfide ameliorates chronic renal failure in rats by inhibiting apoptosis and inflammation through ros/mapk and nf-?b signaling pathways. Sci. Rep. 7, 455. 10.1038/s41598-017-00557-2 28352125 PMC5428696

[B39] Xie K. Chai Y. S. Lin S. H. Xu F. Wang C. J. (2021). Luteolin regulates the differentiation of regulatory T cells and activates il-10-dependent macrophage polarization against acute lung injury. J. Immunol. Res. 2021, 8883962. 10.1155/2021/8883962 33532509 PMC7834791

[B40] Yan X. Wang Z. Barlow P. (1992). Quantitative estimation of garlic oil content in garlic oil based health products. Food Chem. 45, 135–139. 10.1016/0308-8146(92)90024-v

[B41] You S. Nakanishi E. Kuwata H. Chen J. Nakasone Y. He X. (2013). Inhibitory effects and molecular mechanisms of garlic organosulfur compounds on the production of inflammatory mediators. Mol. Nutr. Food Res. 57, 2049–2060. 10.1002/mnfr.201200843 23766070

[B42] Zhang F. Zhang Y. Wang K. Zhu X. Lin G. Zhao Z. (2015). Diallyl trisulfide inhibits naphthalene-induced oxidative injury and the production of inflammatory responses in A549 cells and mice. Int. Immunopharmacol. 29, 326–333. 10.1016/j.intimp.2015.10.033 26548347

[B43] Zhang R. Ai X. Duan Y. Xue M. He W. Wang C. (2017). Kaempferol ameliorates H9n2 swine influenza virus-induced acute lung injury by inactivation of tlr4/myd88-mediated nf-?b and mapk signaling pathways. Biomed. Pharmacother. 89, 660–672. 10.1016/j.biopha.2017.02.081 28262619

[B44] Zhang X.-N. Zhao N. Guo F.-F. Wang Y.-R. Liu S.-X. Zeng T. (2022). Diallyl disulfide suppresses the lipopolysaccharide-driven inflammatory response of macrophages by activating the Nrf2 pathway. Food Chem. Toxicol. 159, 112760. 10.1016/j.fct.2021.112760 34896185

[B45] Zhao H.-J. Li M.-J. Zhang M.-P. Wei M.-K. Shen L.-P. Jiang M. (2019). Allyl Methyl Trisulfide Protected against Acetaminophen (Paracetamol)-Induced Hepatotoxicity by Suppressing Cyp2e1 and Activating Nrf2 in Mouse Liver. Food Funct. 10, 2244–2253. 10.1039/c9fo00170k 30958500

[B46] Zhou Y. Xia H. Zhao L. Mei F. Li M. You Y. (2019). Sb203580 attenuates acute lung injury and inflammation in rats with acute pancreatitis in pregnancy. Inflammopharmacology 27, 99–107. 10.1007/s10787-018-0522-9 30094758

