# Supplementary material for: Allyl methyl trisulfide protected against LPS-induced acute lung injury in mice via inhibition of the NF-κB and MAPK pathways
Source: Front Pharmacol. 2022 Aug 8;13:919898. doi: 10.3389/fphar.2022.919898 (PMC9394683; doi:10.3389/fphar.2022.919898)
Supplement: Supplementary file 1 [file DataSheet4.DOCX]

**Supplementary Figure 3.** Western blot bands.

**pIKBα**


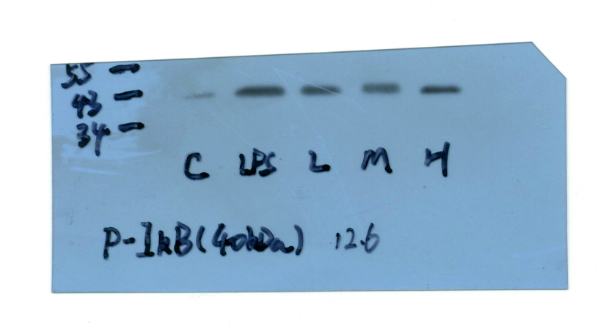


**IKBα**


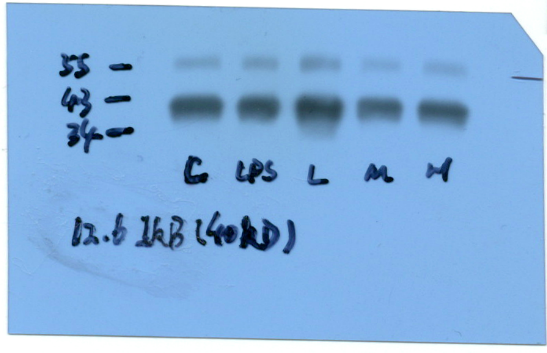


**Cytoplasm NF-κB p65**


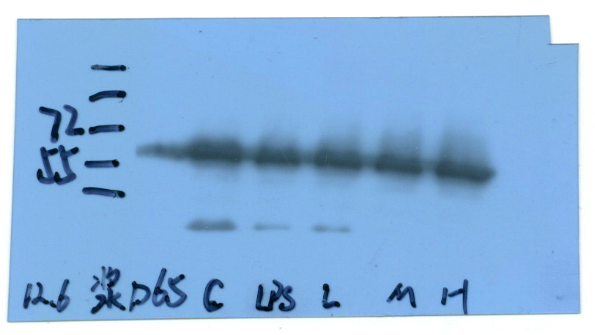


**β-actin**


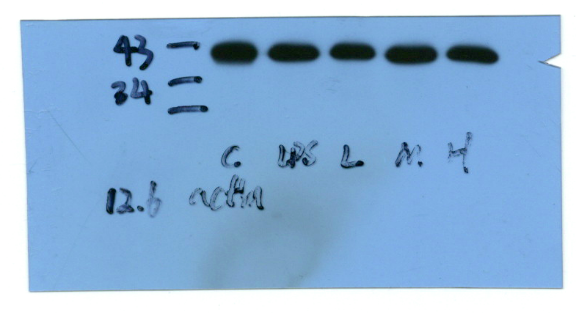


**Nuclear NF-κB p65**


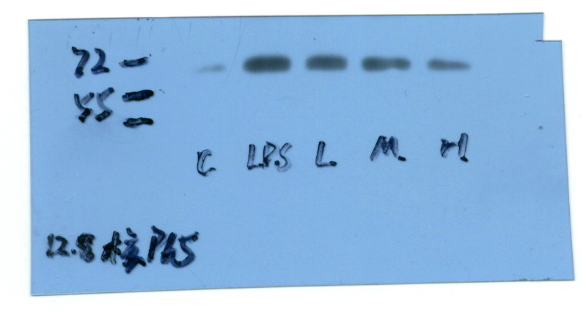


**Lamin B**


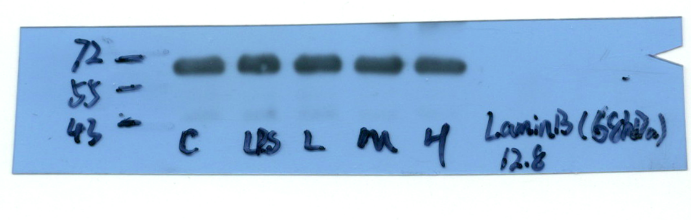


**pERK**


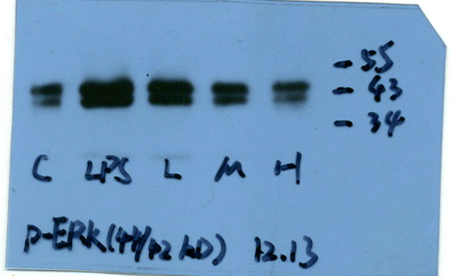


**ERK**


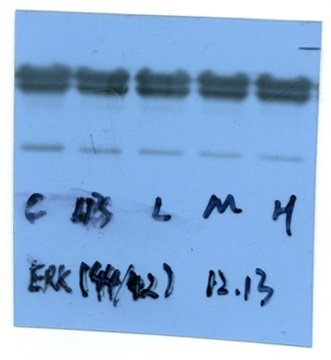


**pSAPK/JNK**


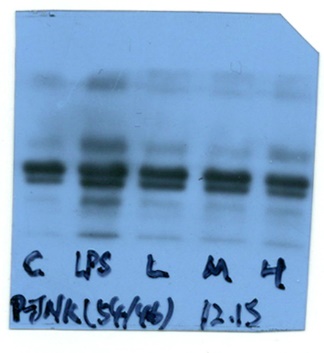


**SAPK/JNK**

**
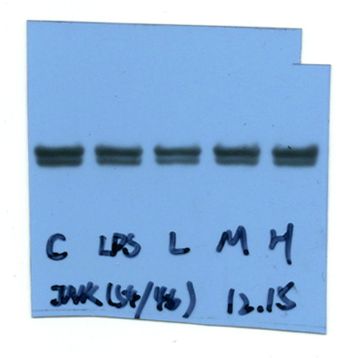
**

**phospho-p38 MAPK**

**
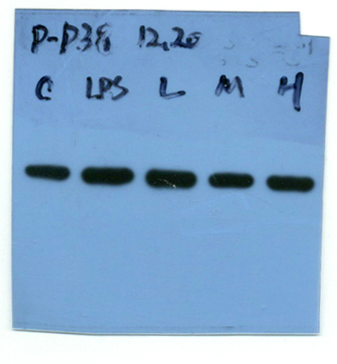
**

**p38 MAPK**

**
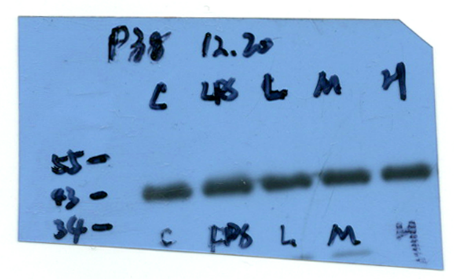
**
