# Supplementary material for: Allyl methyl trisulfide protected against LPS-induced acute lung injury in mice via inhibition of the NF-κB and MAPK pathways
Source: Front Pharmacol. 2022 Aug 8;13:919898. doi: 10.3389/fphar.2022.919898 (PMC9394683; doi:10.3389/fphar.2022.919898)
Supplement: Supplementary file 2 [file DataSheet3.DOCX]

**Supplementary Figure 2.** Immunohistochemical staining with F4/80 and CD68 antibody.

| **F4/80** | | **CD68** | |
| --- | --- | --- | --- |
| **Control Group** | | | |
| 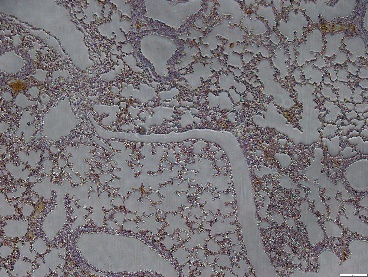 | 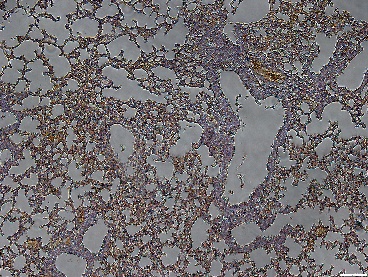 | 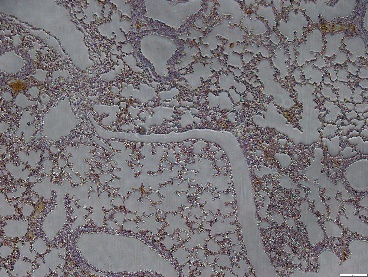 | 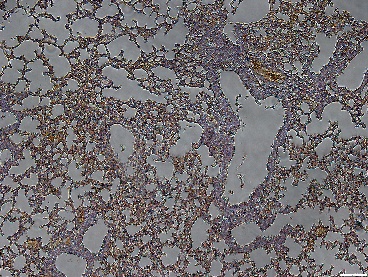 |
| **LPS** | | | |
| 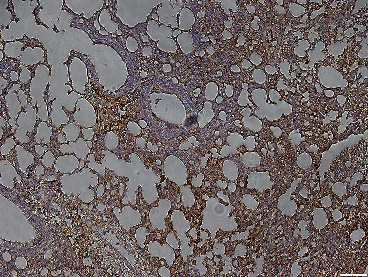 | 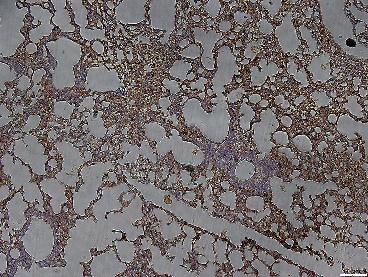 | 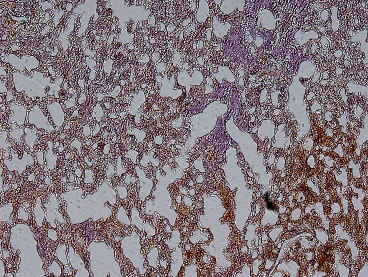 | 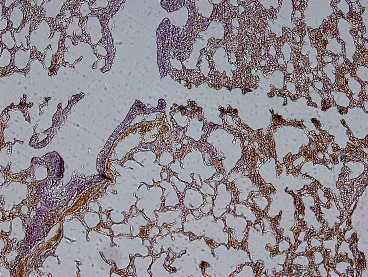 |
| **LPS+AMTS 25 mg/kg** | | | |
| 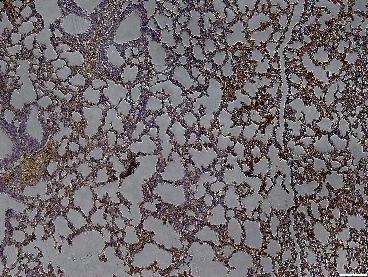 | 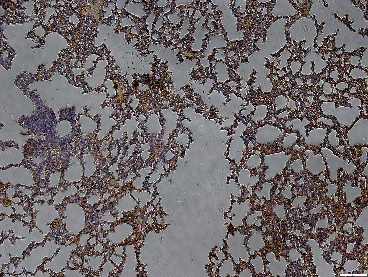 | 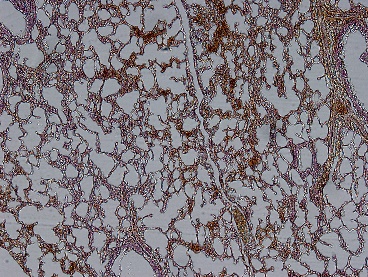 | 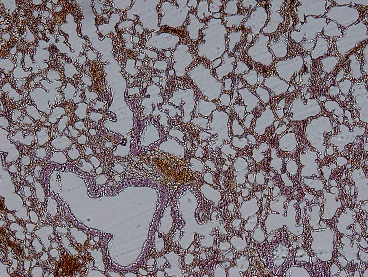 |
| **LPS+AMTS 50 mg/kg** | | | |
| 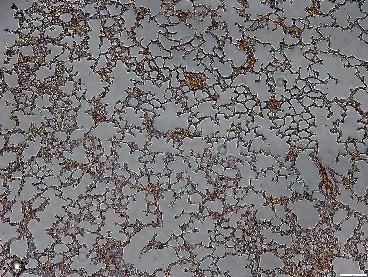 | 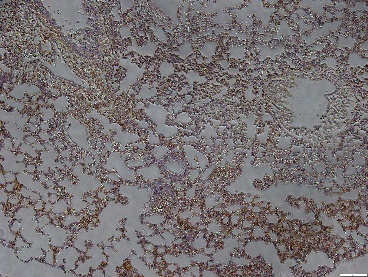 | 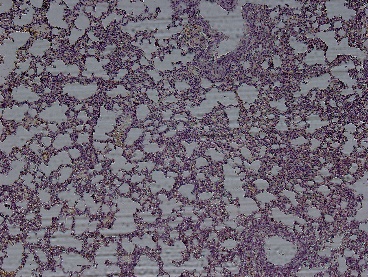 | 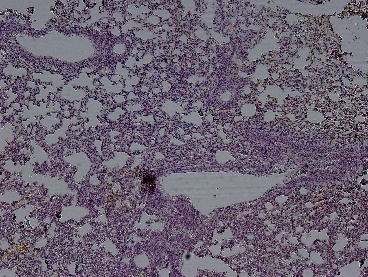 |
| **LPS+AMTS 100 mg/kg** | | | |
| 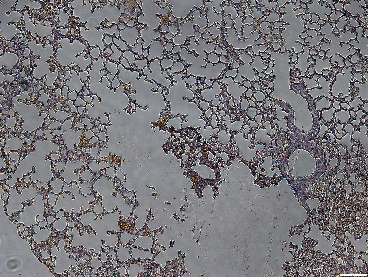 | 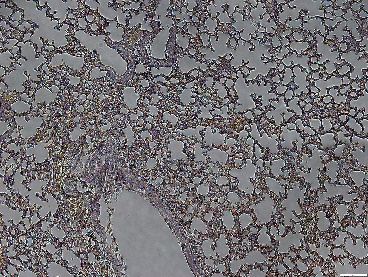 | 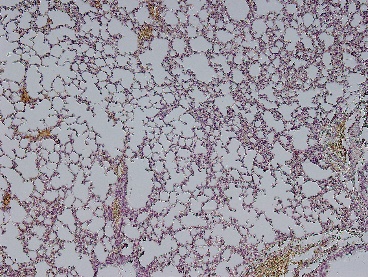 | 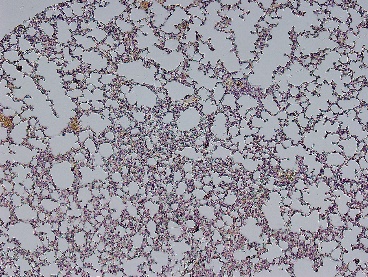 |
