# Supplementary figures and images for: Allyl methyl trisulfide protected against LPS-induced acute lung injury in mice via inhibition of the NF-κB and MAPK pathways
Source: Front Pharmacol. 2022 Aug 8;13:919898. doi: 10.3389/fphar.2022.919898 (PMC9394683; doi:10.3389/fphar.2022.919898)

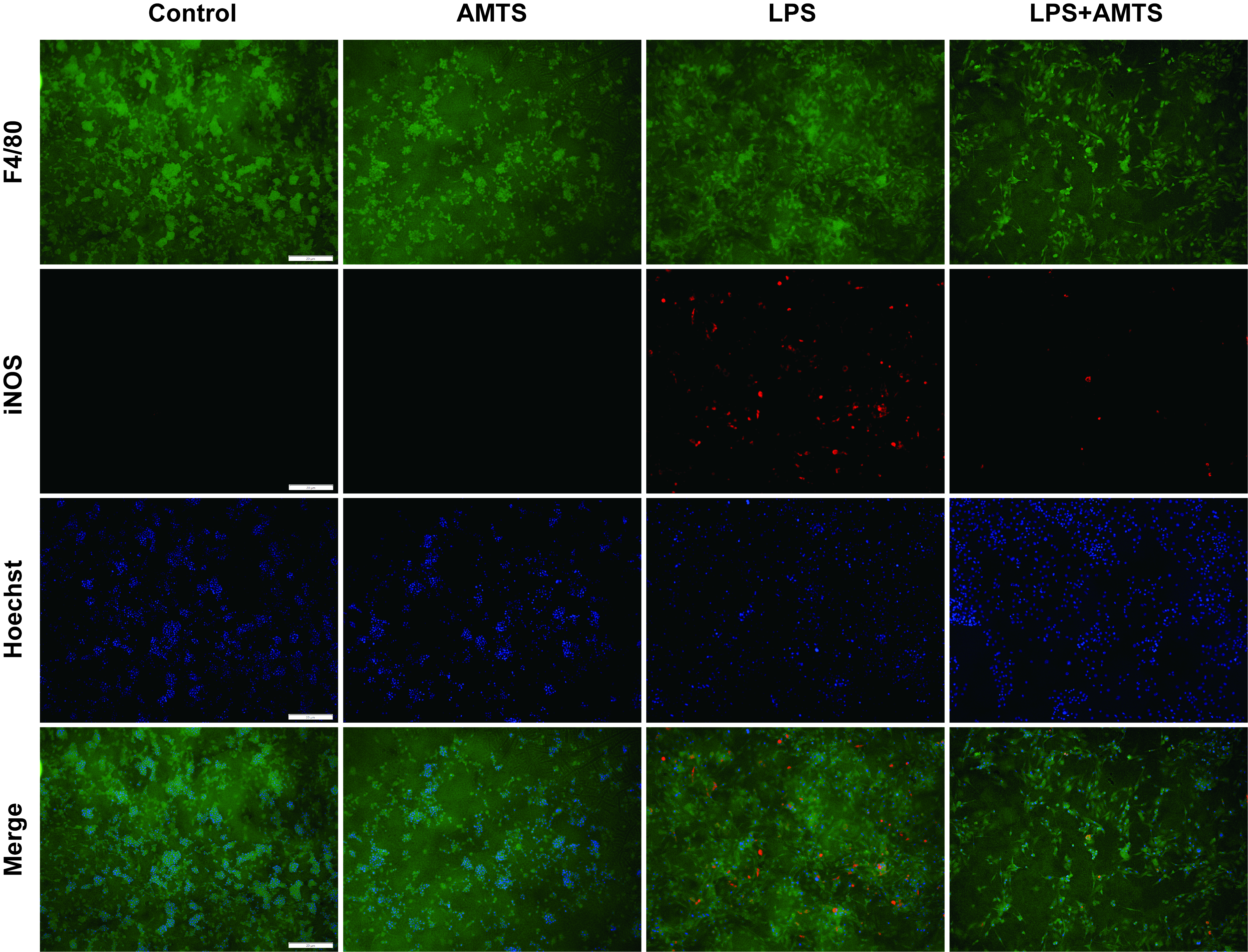

Supplement: Supplementary file 3 [file Image1.TIF]

**Supplementary Figure 2.** Immunohistochemical staining with F4/80 and CD68 antibody.

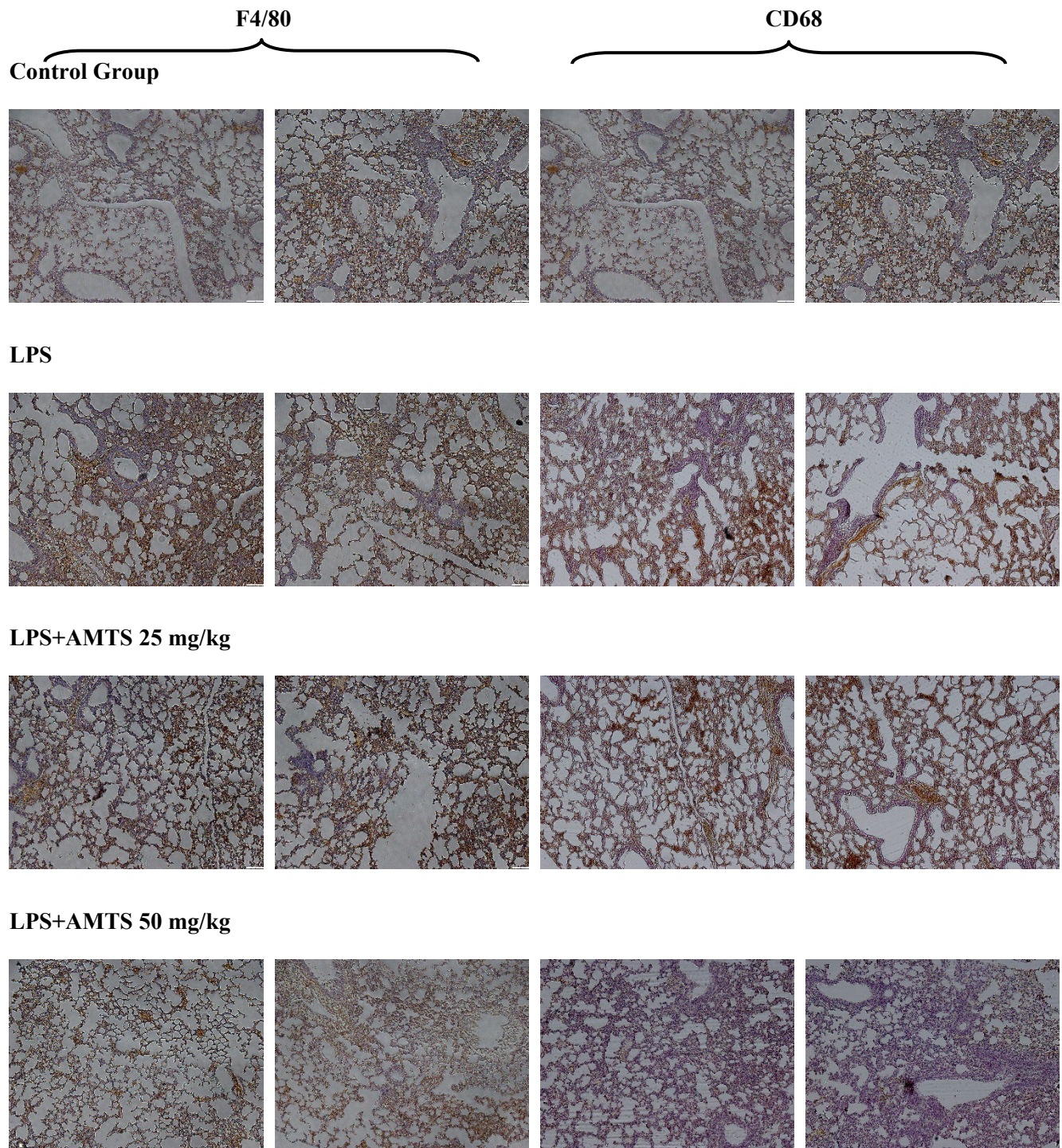

**LPS+AMTS 100 mg/kg**

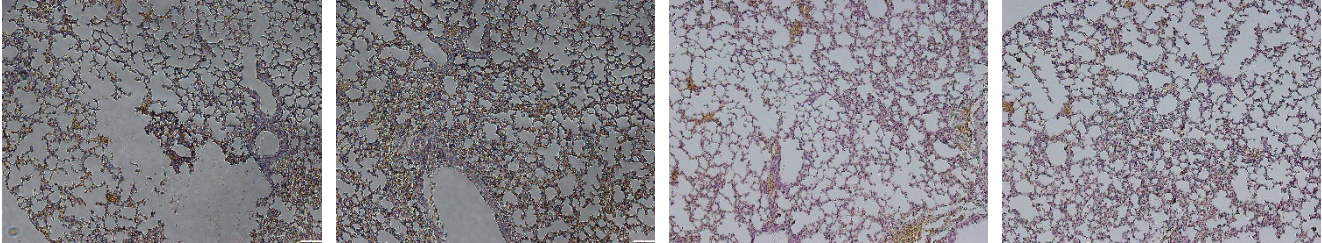

Supplement: Supplementary file 4 [file Image2.pdf]

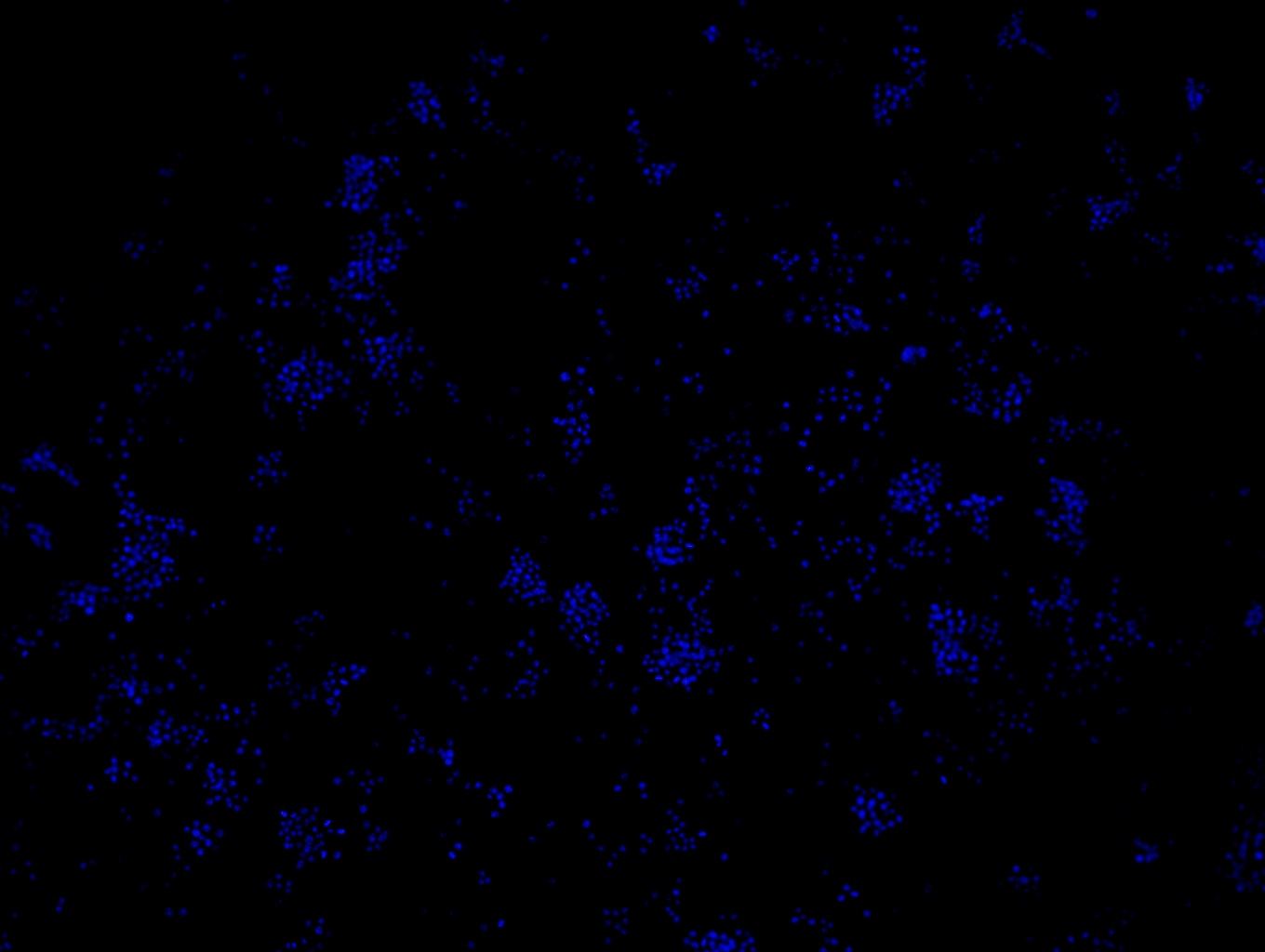

Supplement: Supplementary file 7 [file DataSheet5.ZIP › Immunofluorescence Raw data/AMTS-blue-1.tif]

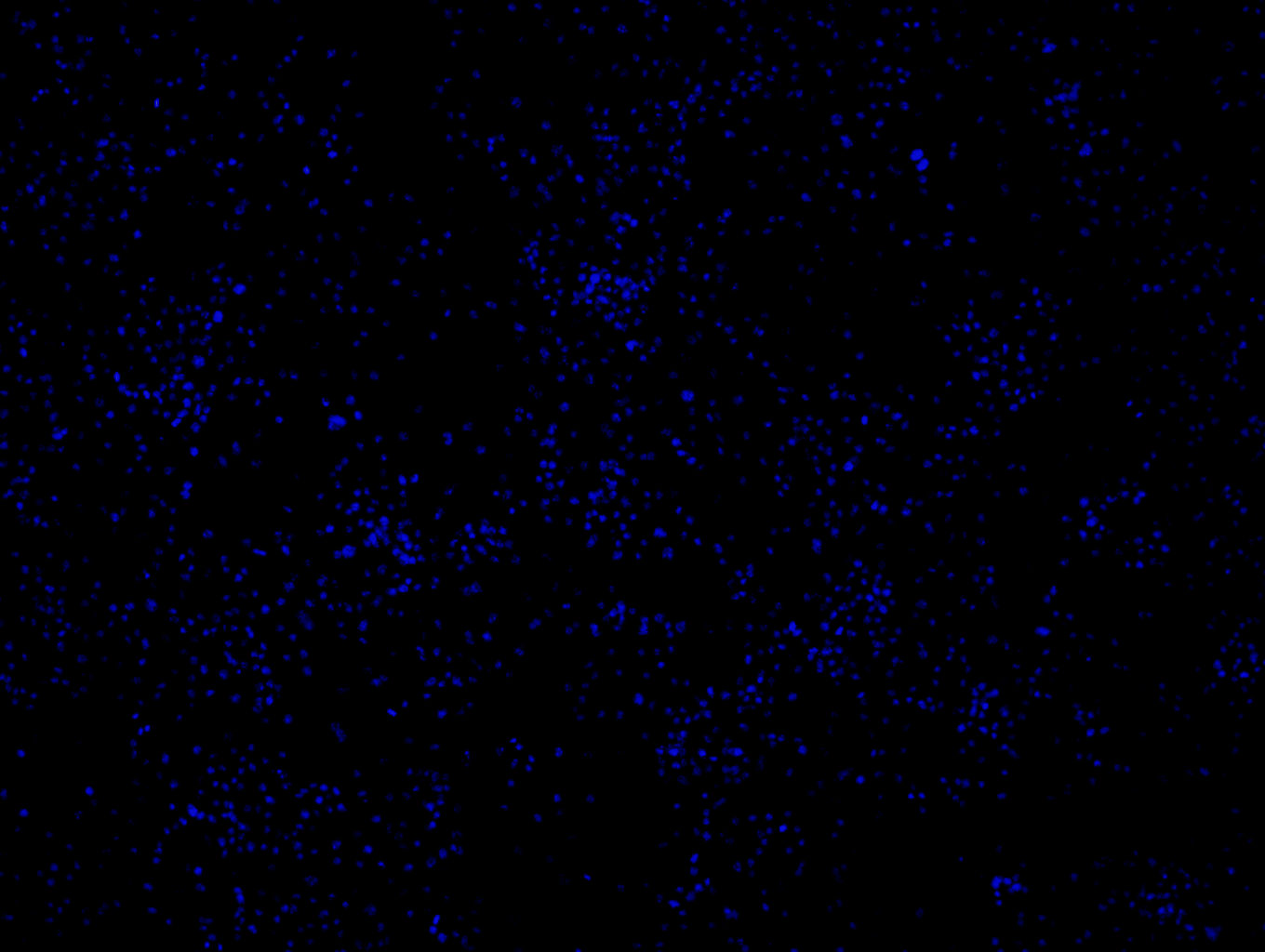

Supplement: Supplementary file 7 [file DataSheet5.ZIP › Immunofluorescence Raw data/AMTS-Blue-2.tif]

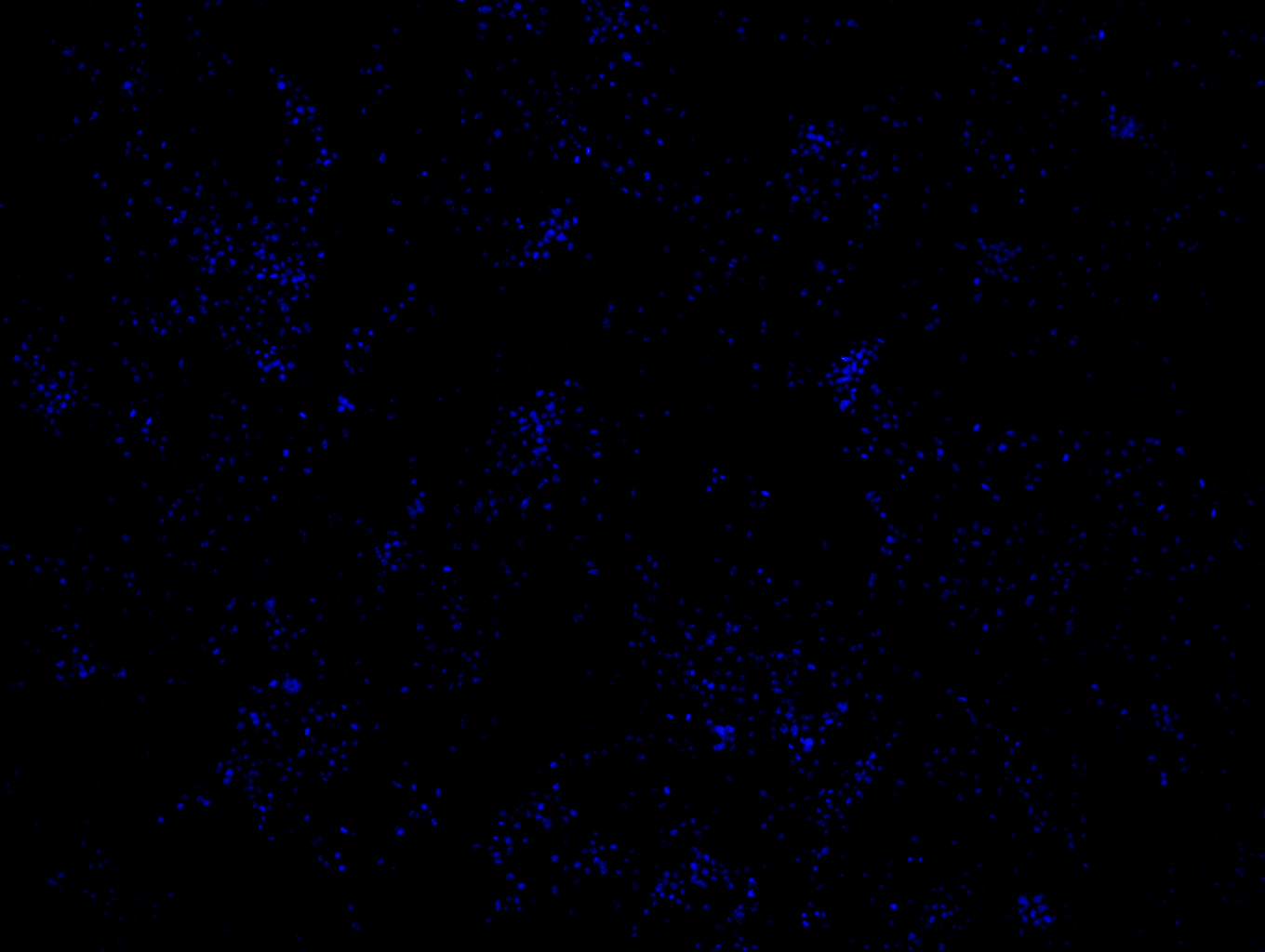

Supplement: Supplementary file 7 [file DataSheet5.ZIP › Immunofluorescence Raw data/AMTS-Blue-3.tif]

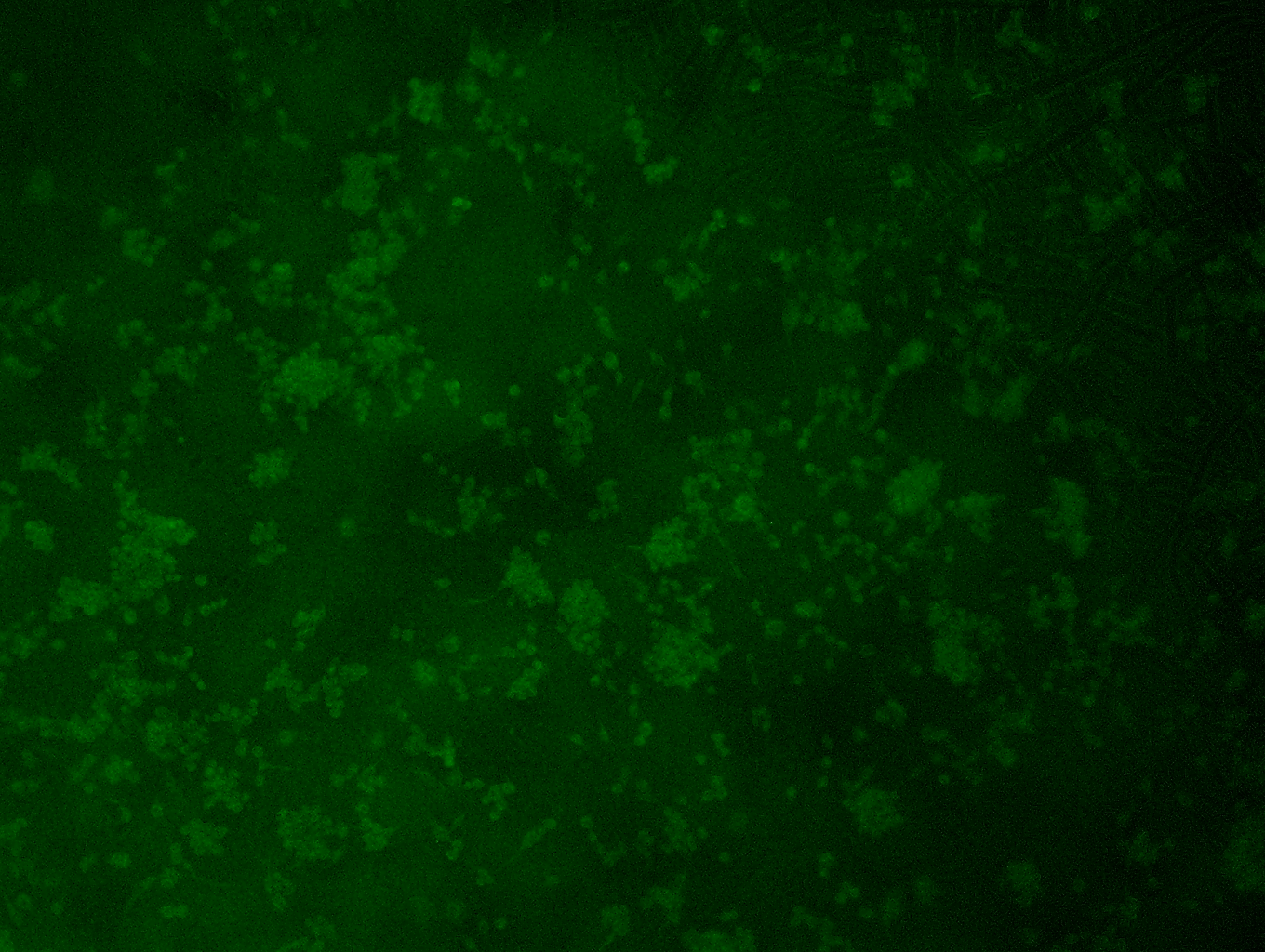

Supplement: Supplementary file 7 [file DataSheet5.ZIP › Immunofluorescence Raw data/AMTS-green-1.tif]

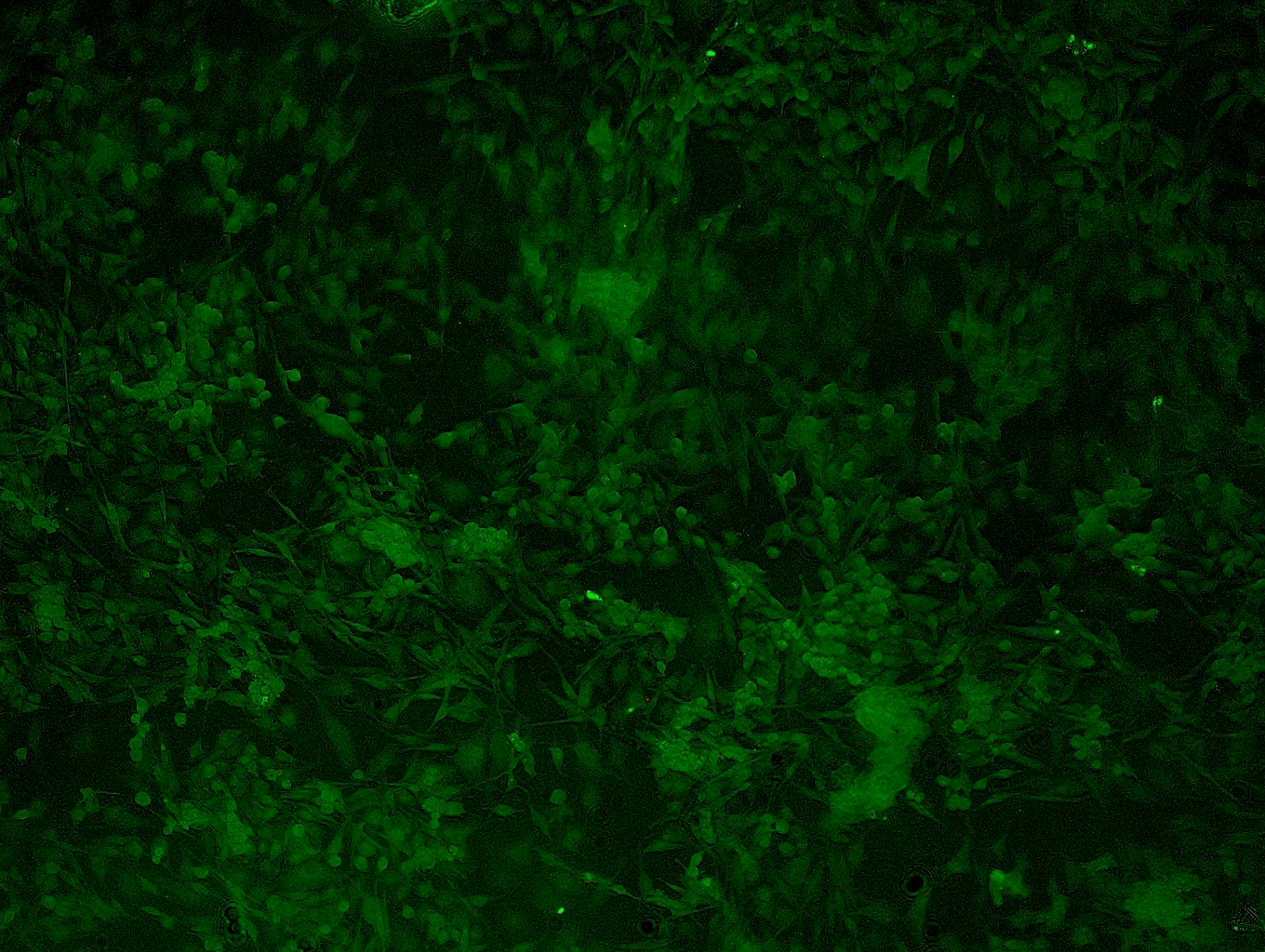

Supplement: Supplementary file 7 [file DataSheet5.ZIP › Immunofluorescence Raw data/AMTS-green-2.tif]

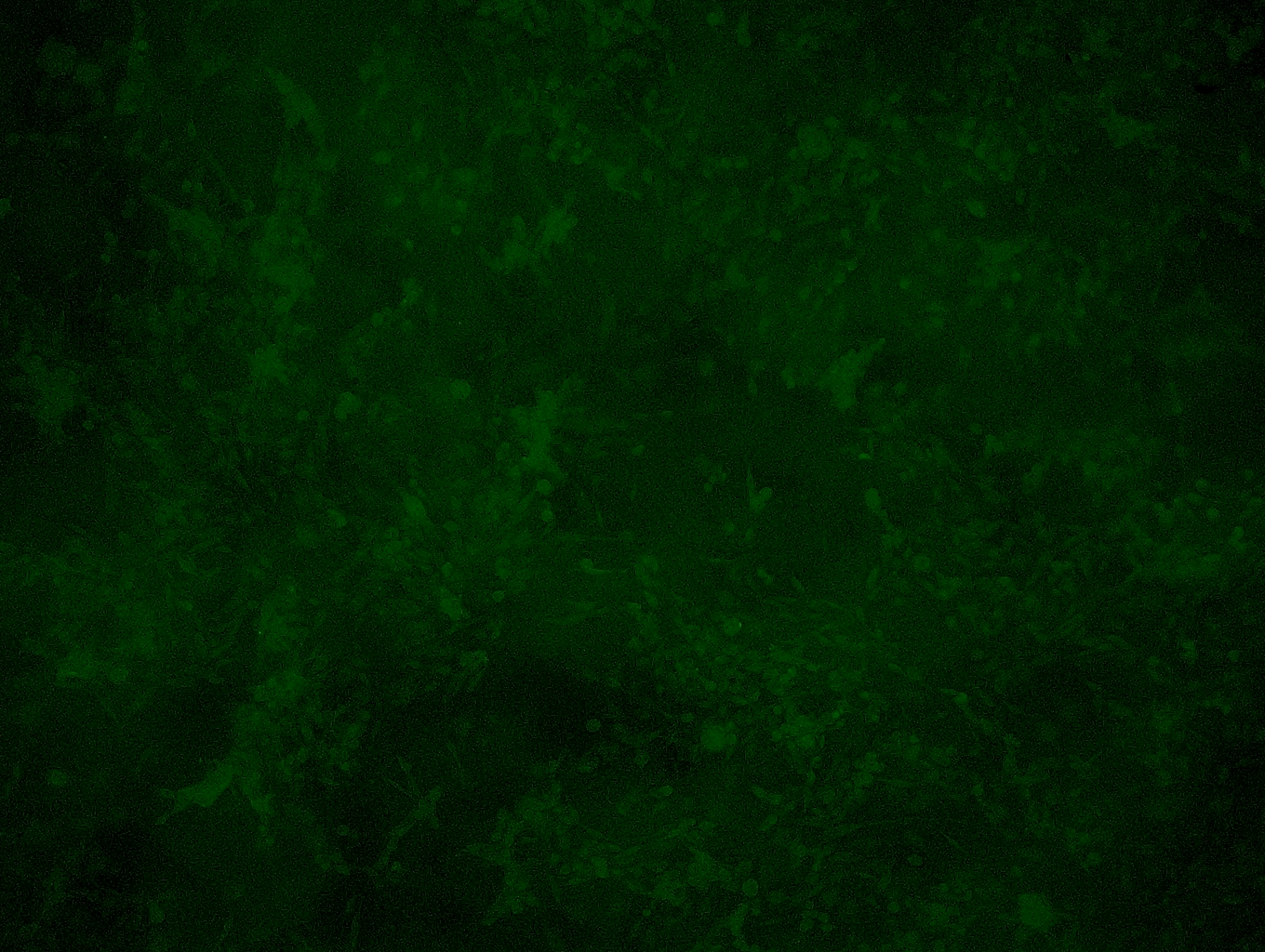

Supplement: Supplementary file 7 [file DataSheet5.ZIP › Immunofluorescence Raw data/AMTS-green-3.tif]

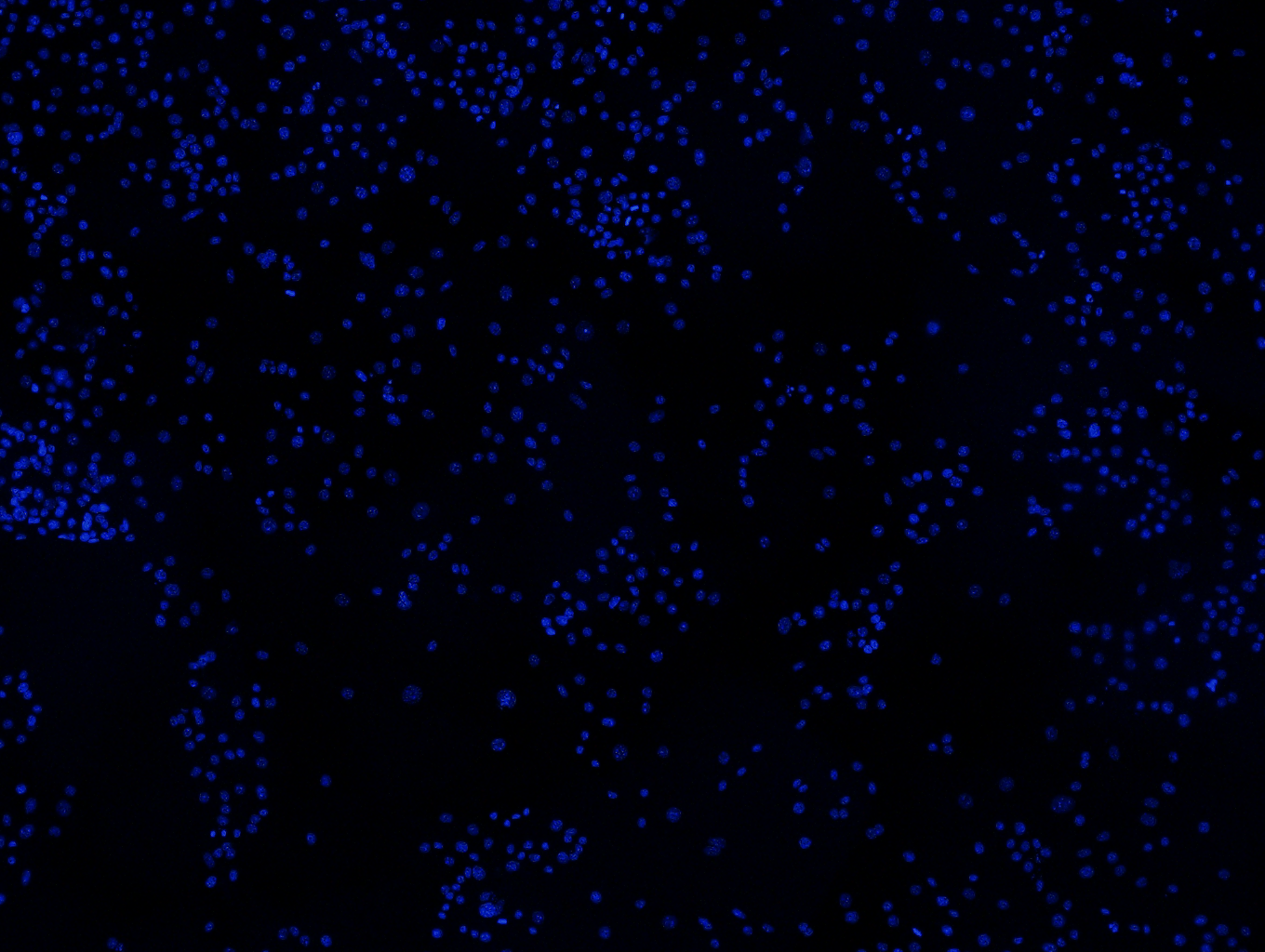

Supplement: Supplementary file 7 [file DataSheet5.ZIP › Immunofluorescence Raw data/AMTS-LPS-Blue-1.tif]

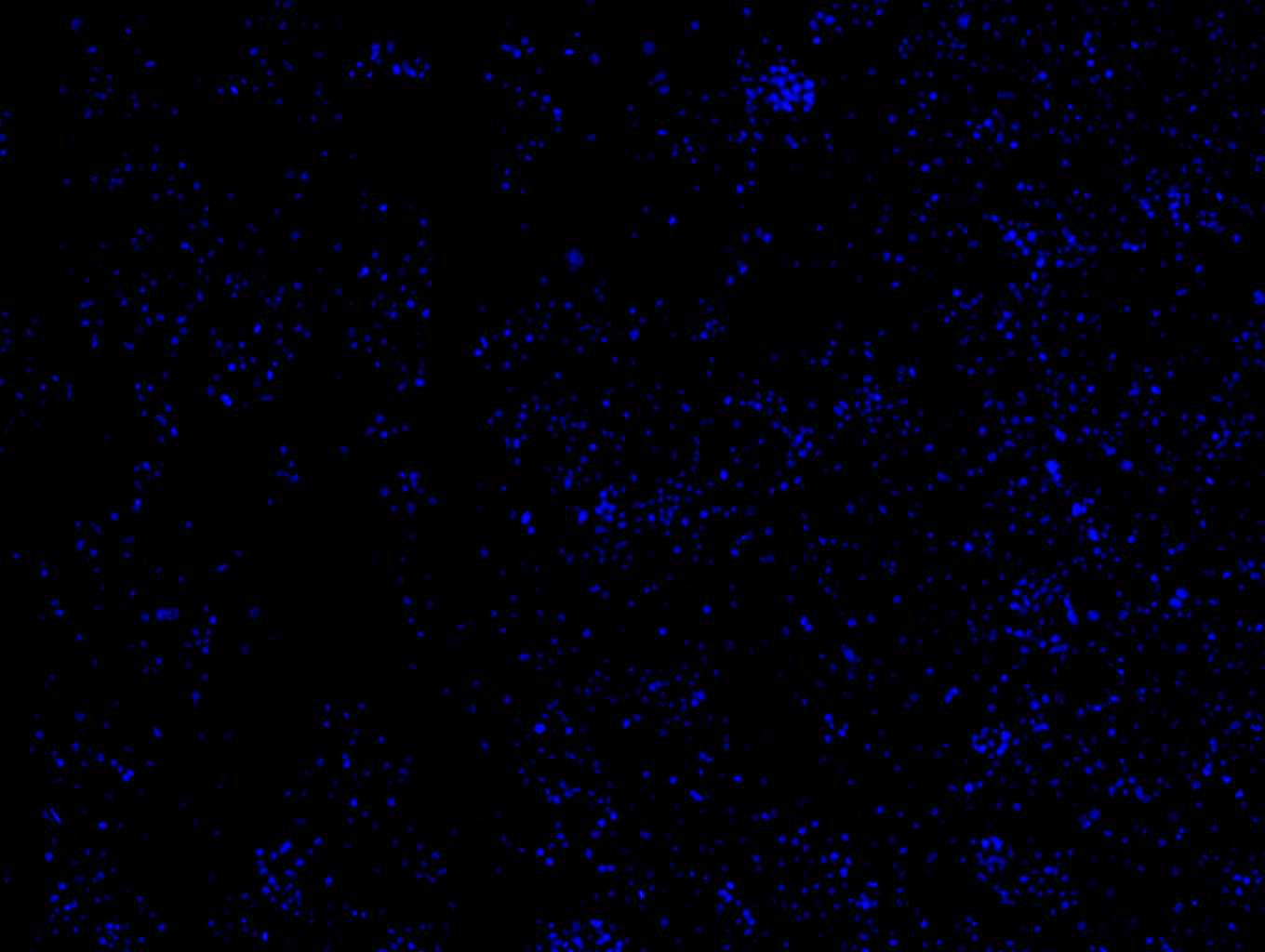

Supplement: Supplementary file 7 [file DataSheet5.ZIP › Immunofluorescence Raw data/AMTS-LPS-Blue-2.tif]

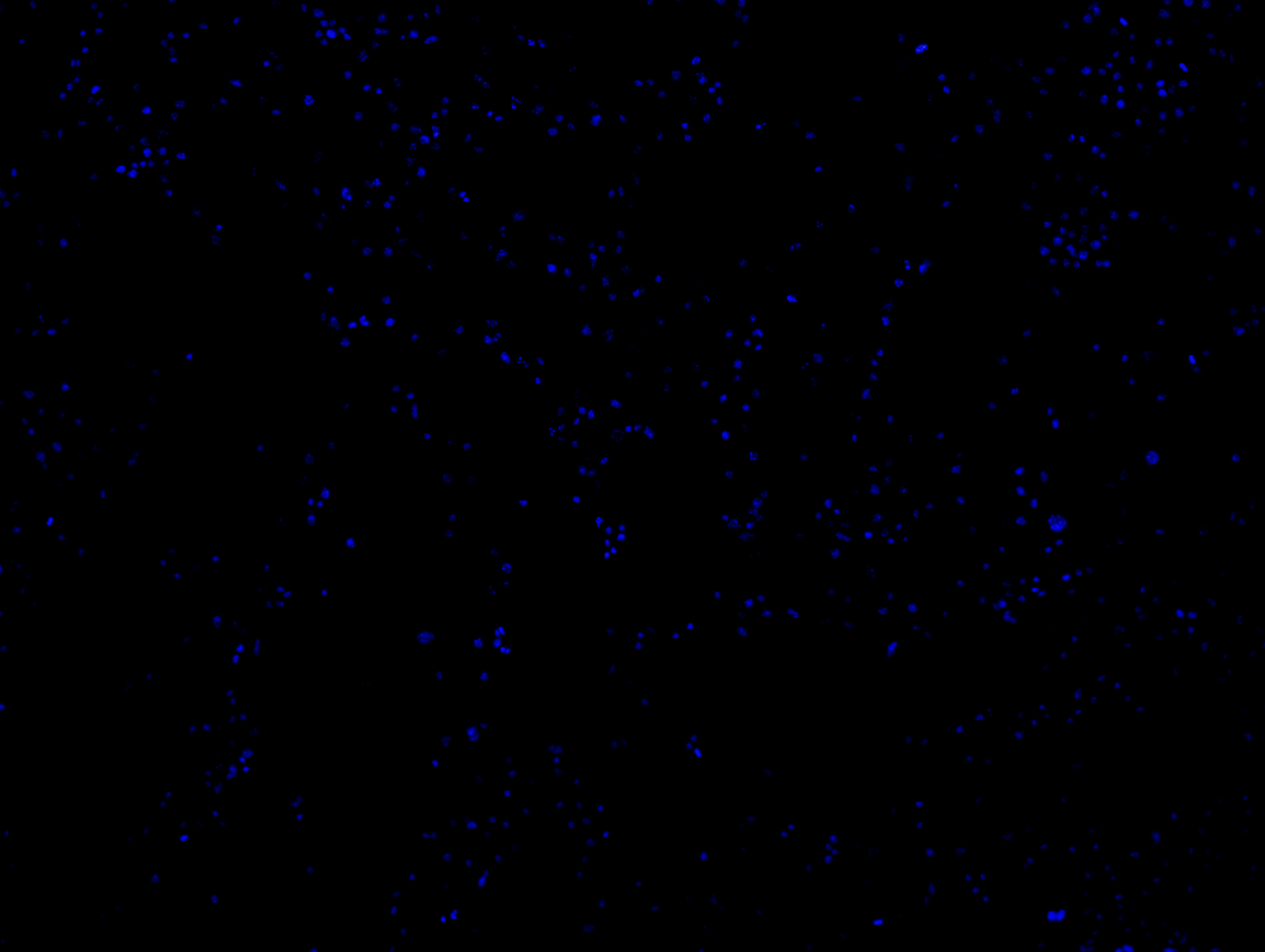

Supplement: Supplementary file 7 [file DataSheet5.ZIP › Immunofluorescence Raw data/AMTS-LPS-Blue-3.tif]

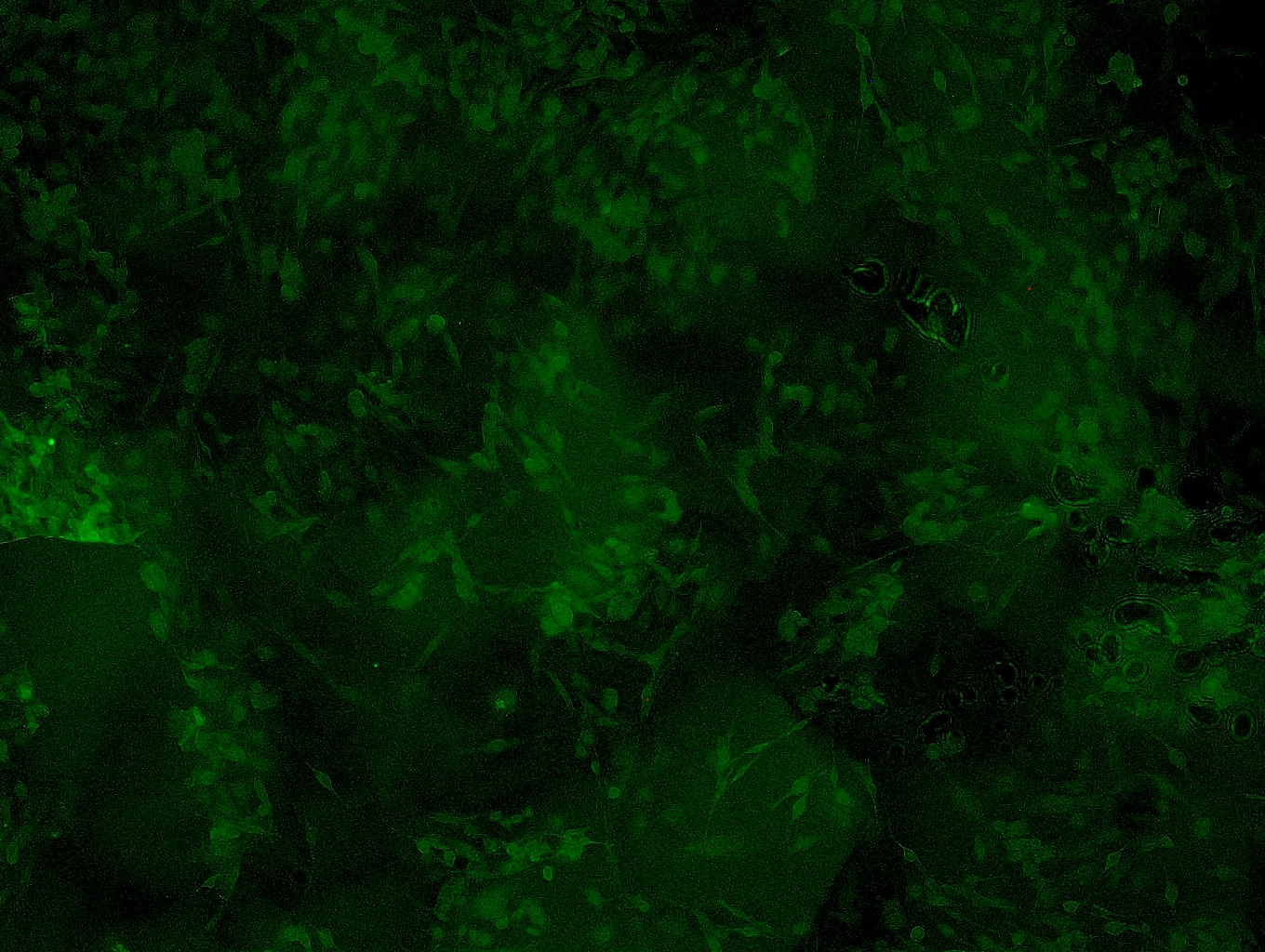

Supplement: Supplementary file 7 [file DataSheet5.ZIP › Immunofluorescence Raw data/AMTS-LPS-green-1.tif]

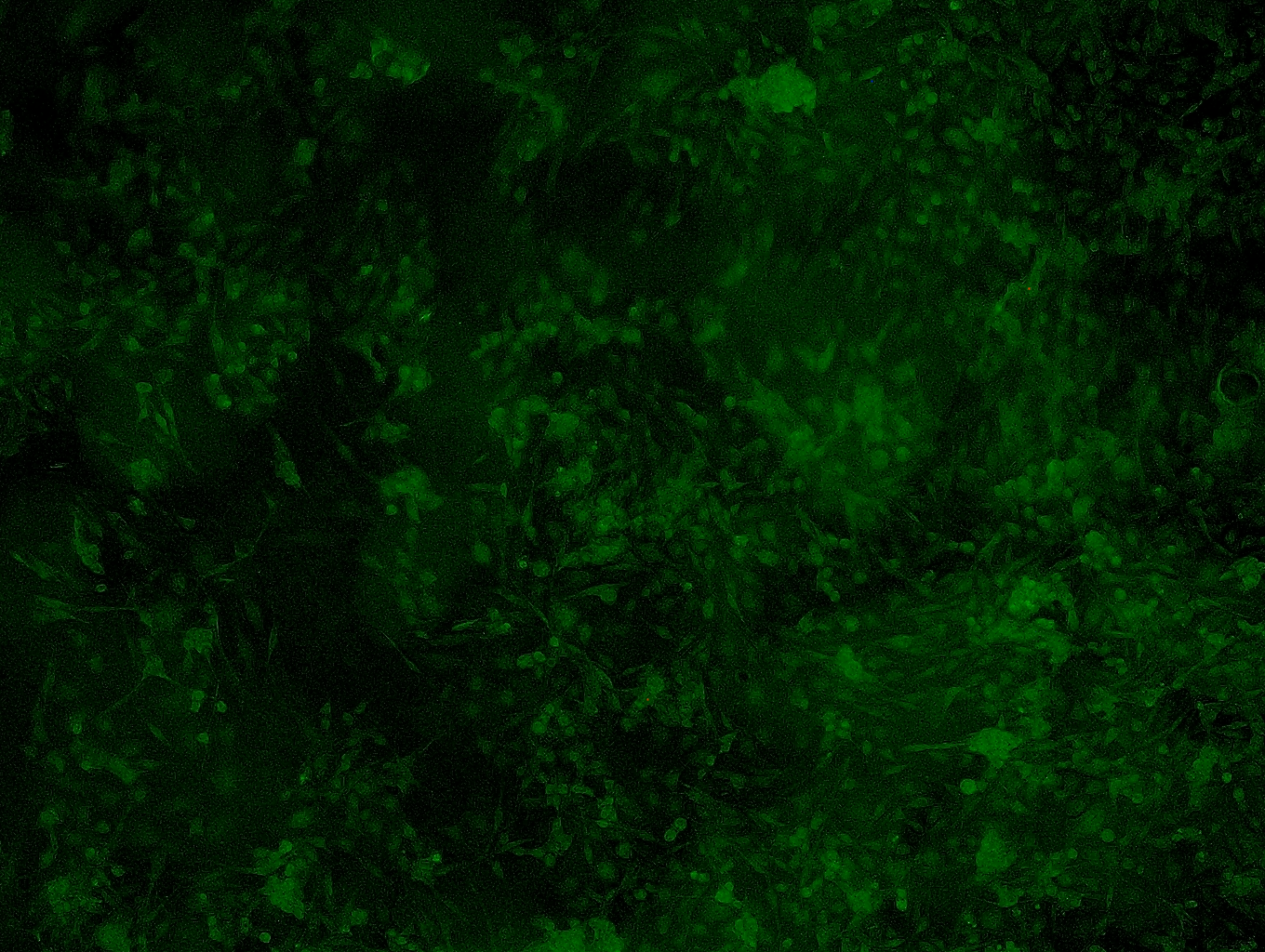

Supplement: Supplementary file 7 [file DataSheet5.ZIP › Immunofluorescence Raw data/AMTS-LPS-green-2.tif]

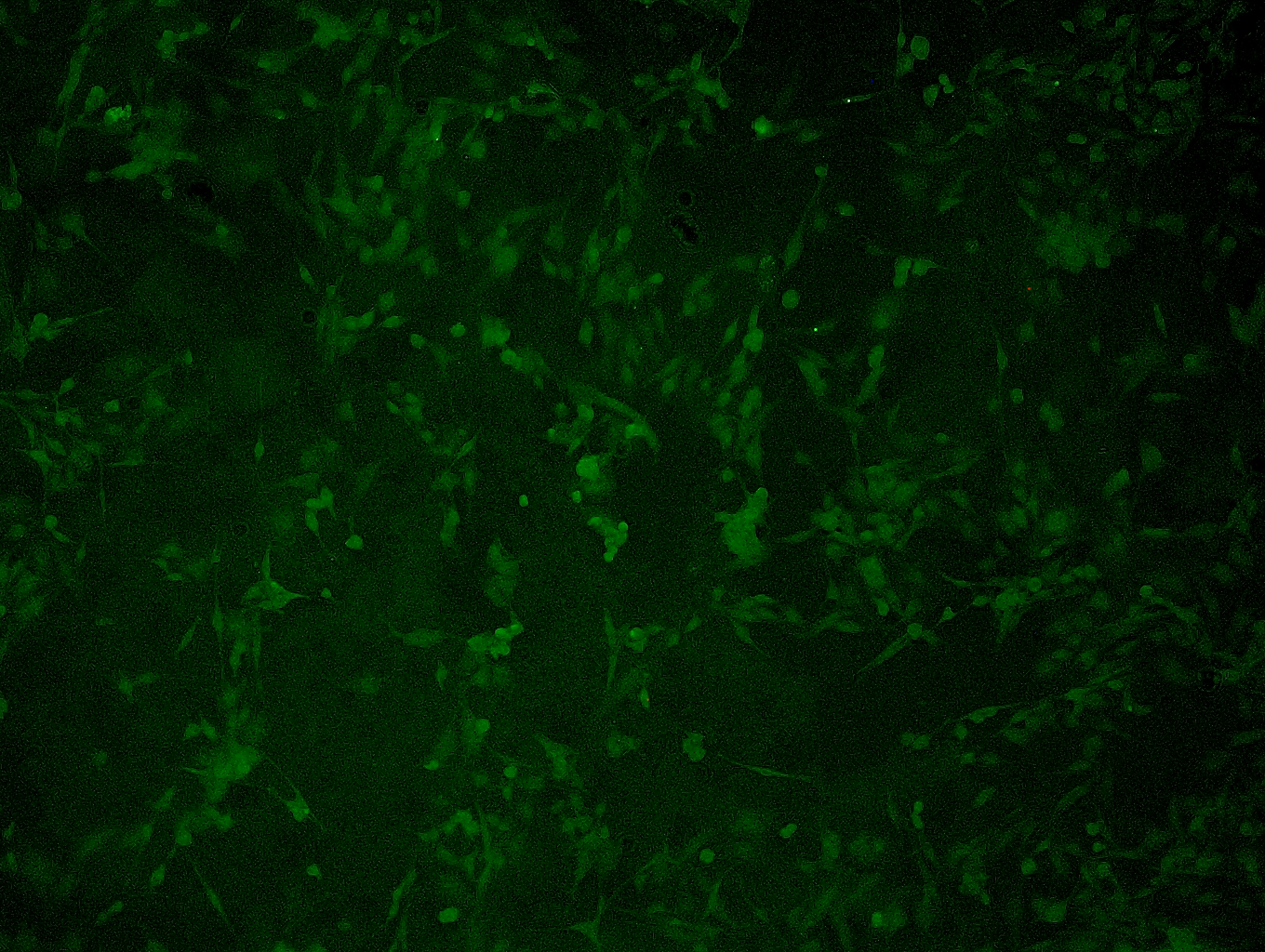

Supplement: Supplementary file 7 [file DataSheet5.ZIP › Immunofluorescence Raw data/AMTS-LPS-green-3.tif]

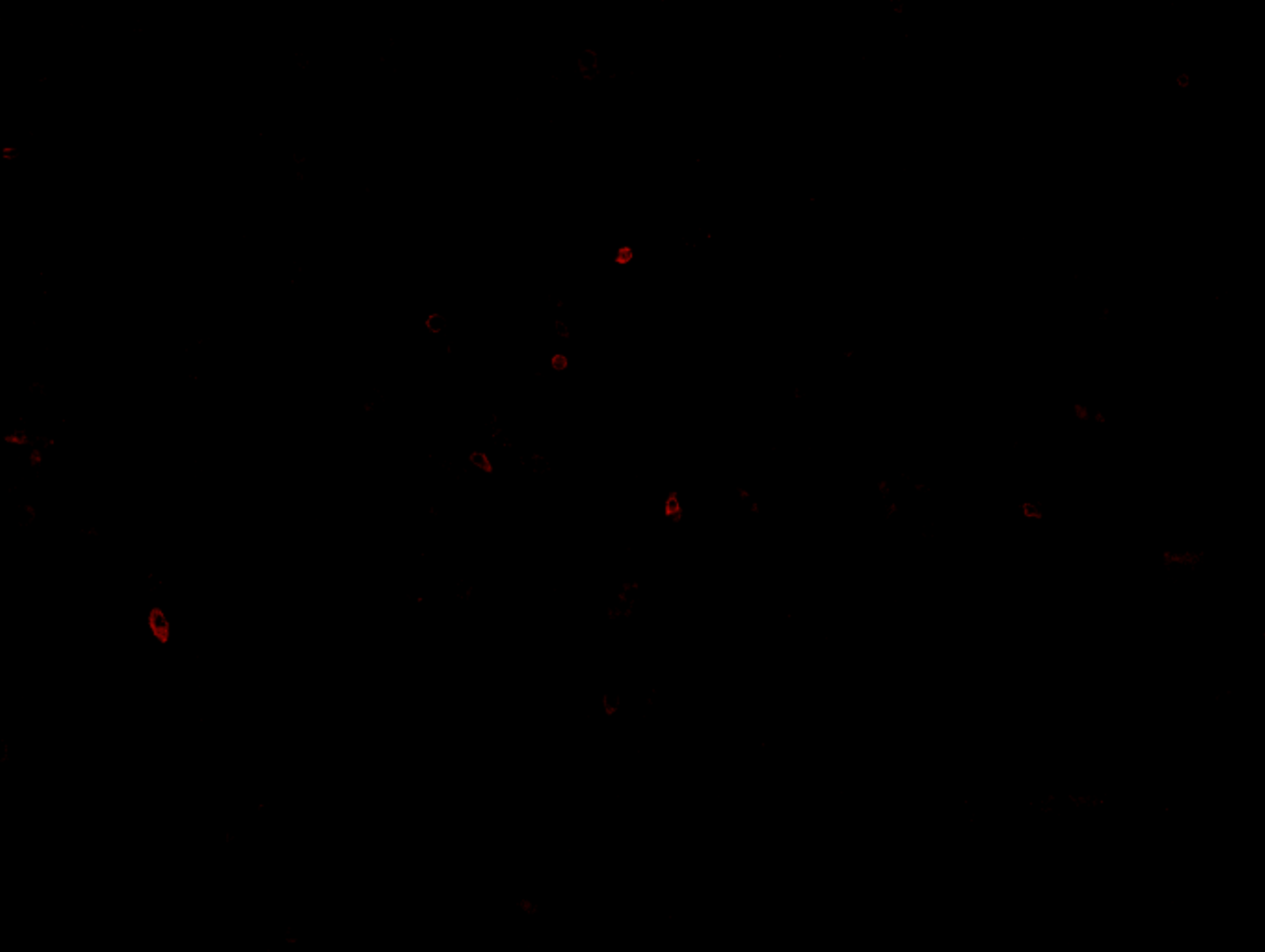

Supplement: Supplementary file 7 [file DataSheet5.ZIP › Immunofluorescence Raw data/AMTS-LPS-red-1.tif]

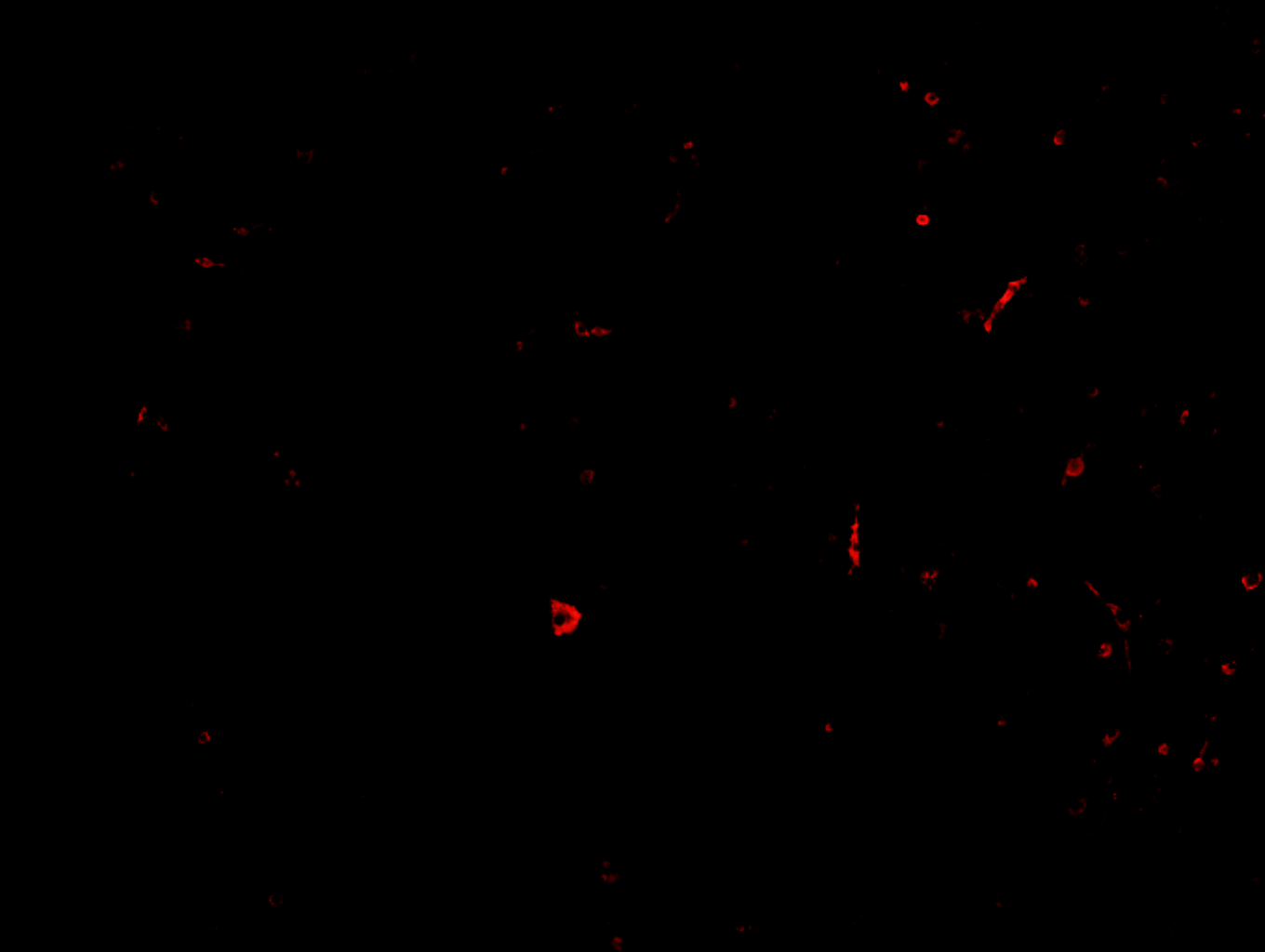

Supplement: Supplementary file 7 [file DataSheet5.ZIP › Immunofluorescence Raw data/AMTS-LPS-red-2.tif]

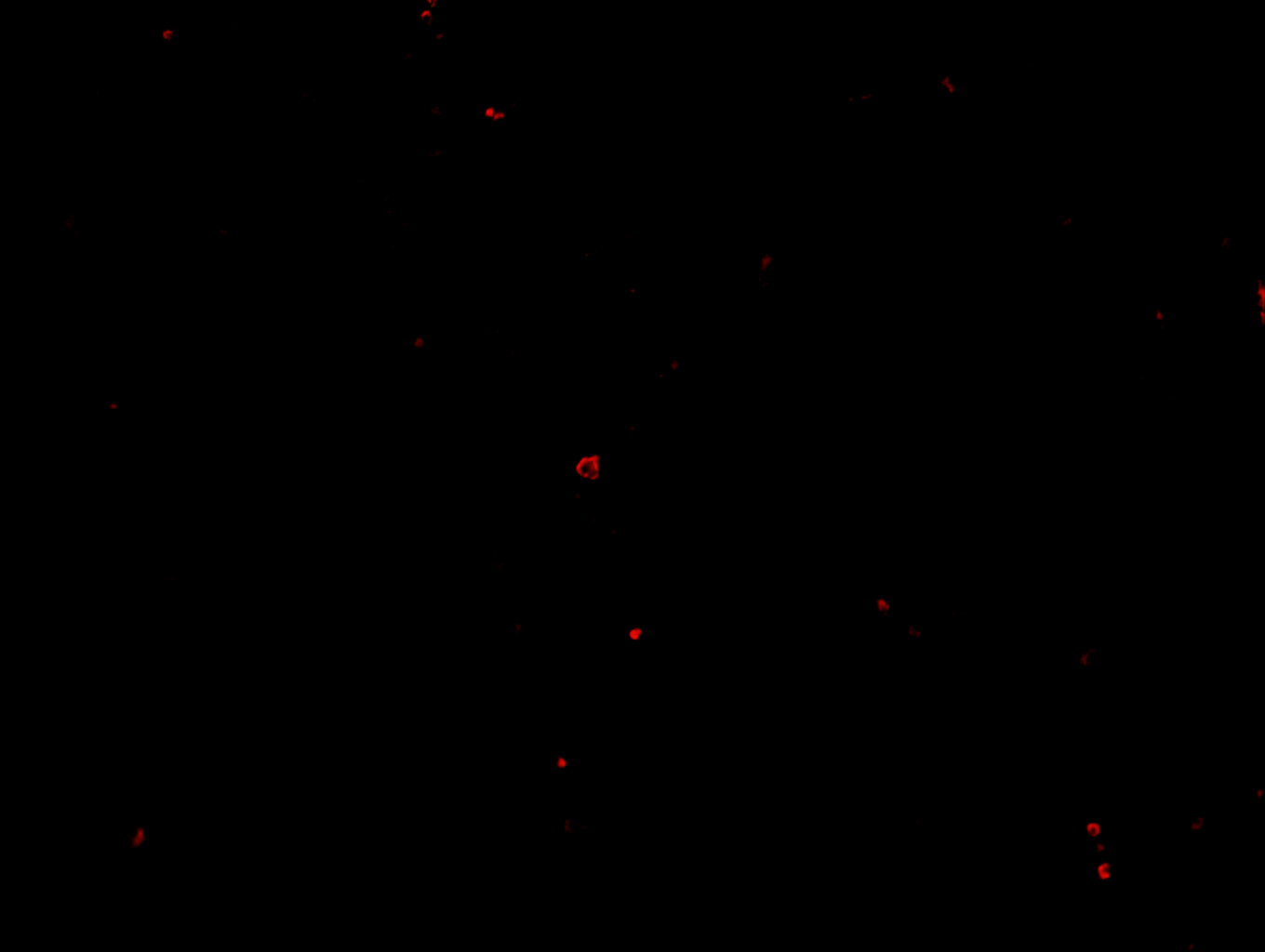

Supplement: Supplementary file 7 [file DataSheet5.ZIP › Immunofluorescence Raw data/AMTS-LPS-red-3.tif]

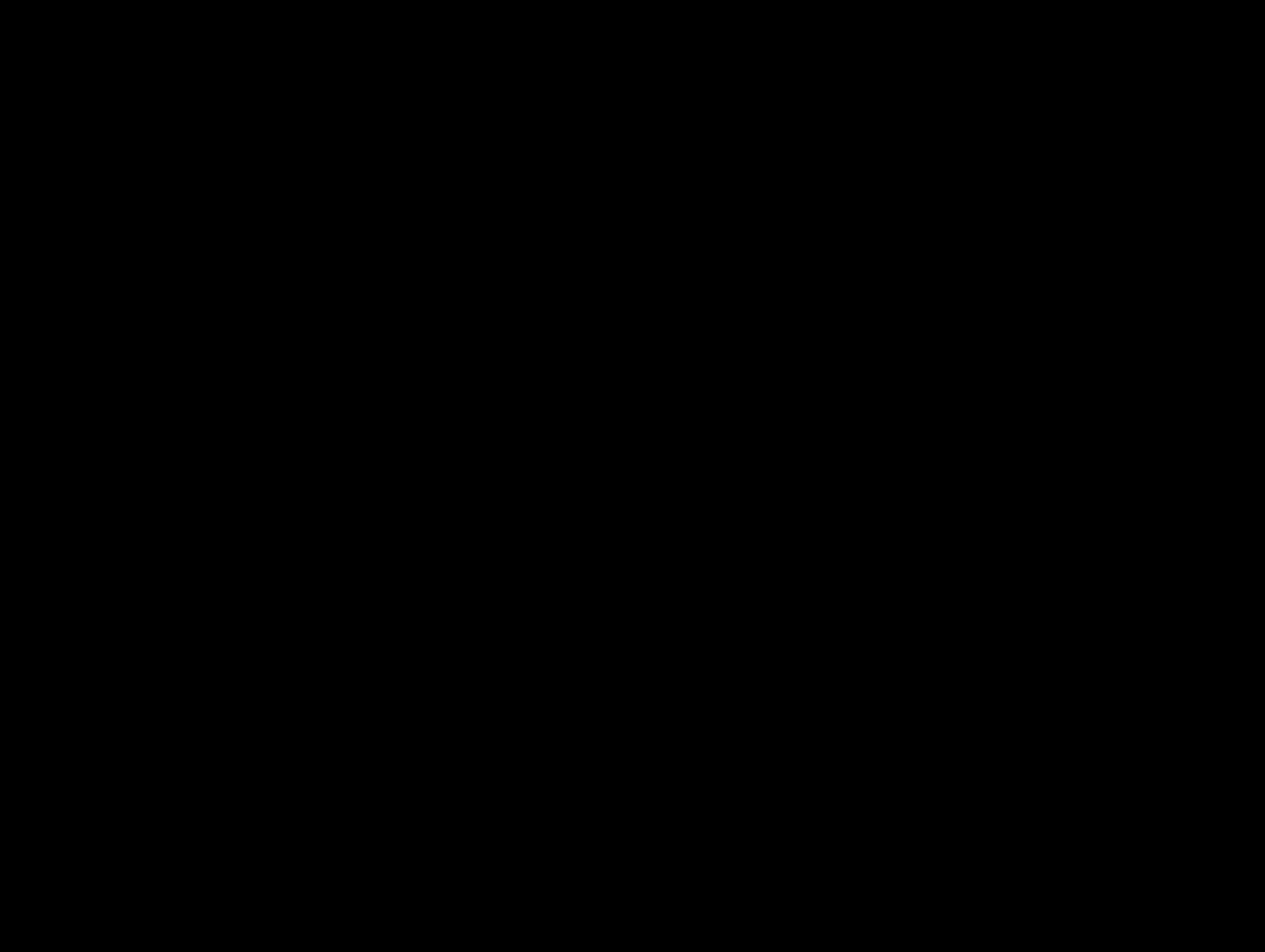

Supplement: Supplementary file 7 [file DataSheet5.ZIP › Immunofluorescence Raw data/AMTS-red-1.tif]

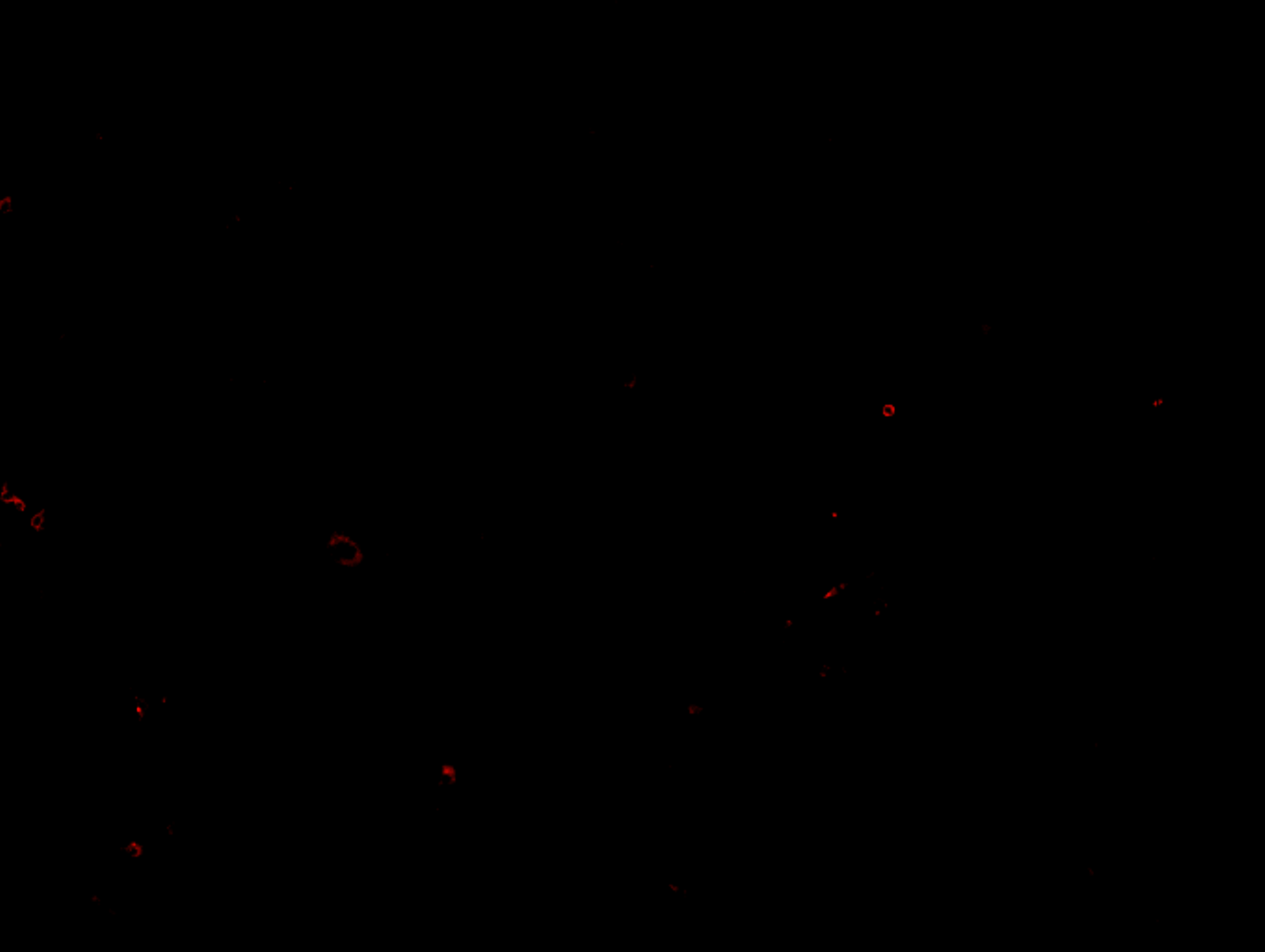

Supplement: Supplementary file 7 [file DataSheet5.ZIP › Immunofluorescence Raw data/AMTS-red-2.tif]

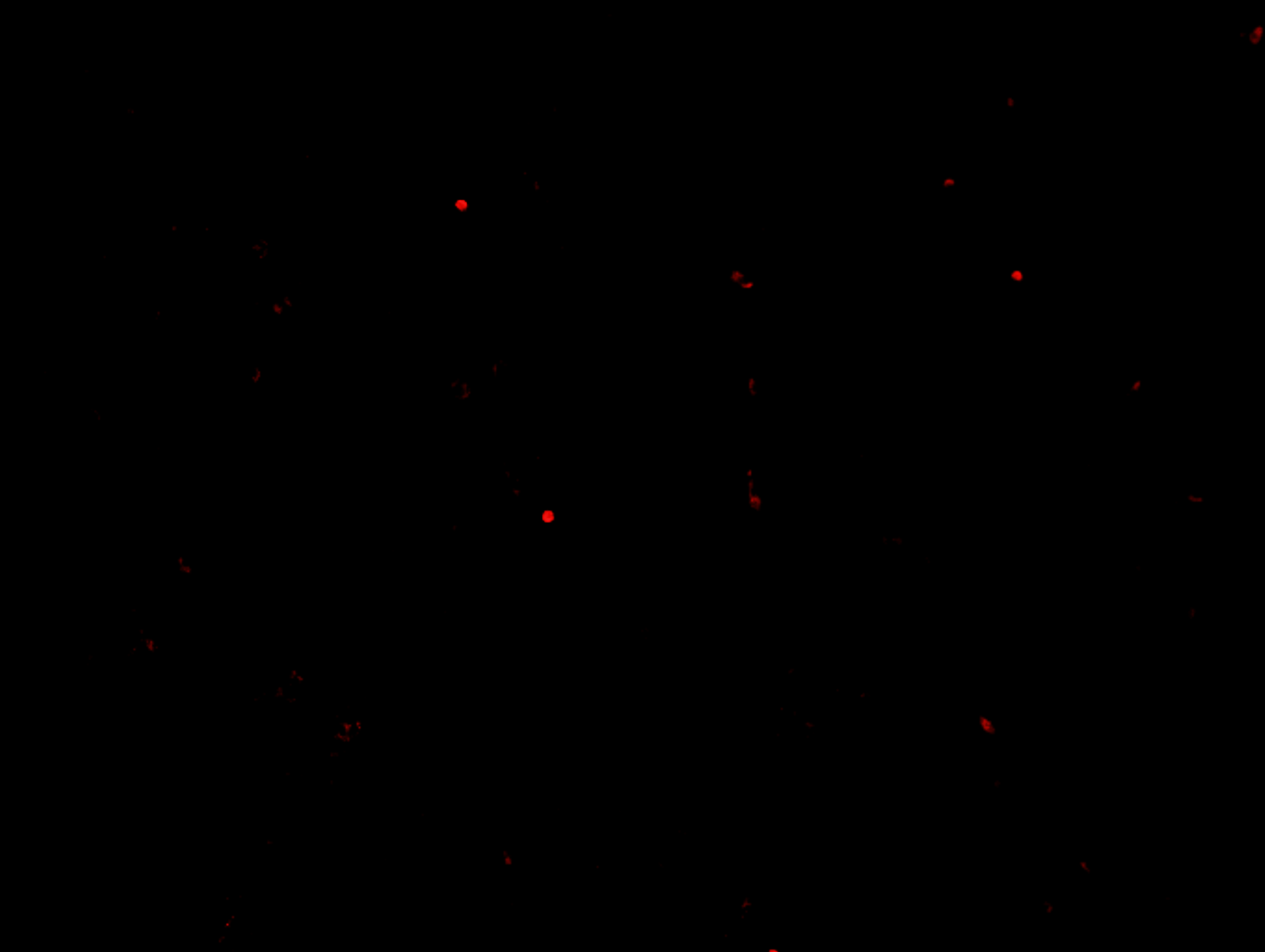

Supplement: Supplementary file 7 [file DataSheet5.ZIP › Immunofluorescence Raw data/AMTS-red-3.tif]

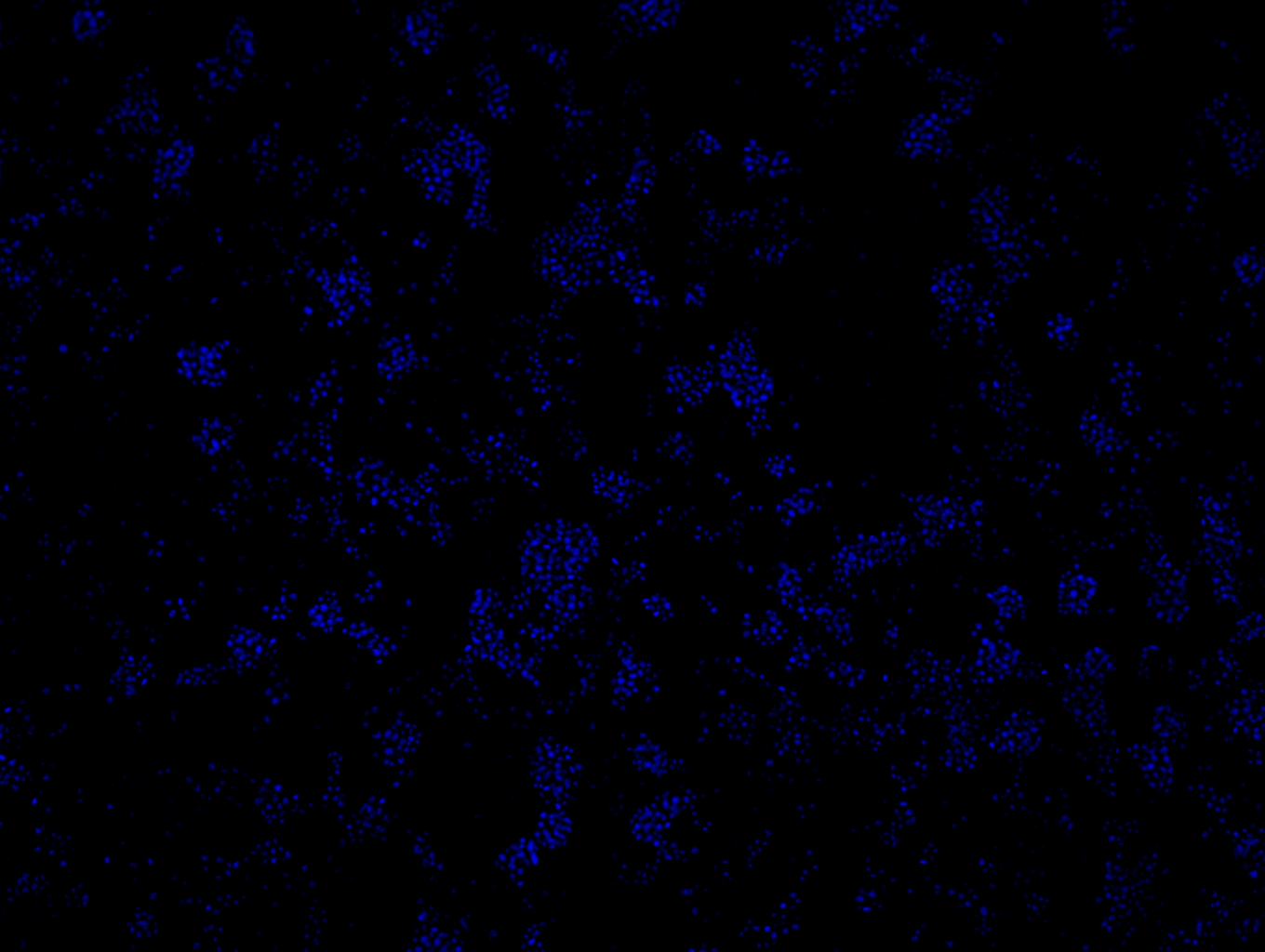

Supplement: Supplementary file 7 [file DataSheet5.ZIP › Immunofluorescence Raw data/C-Blue-1.tif]

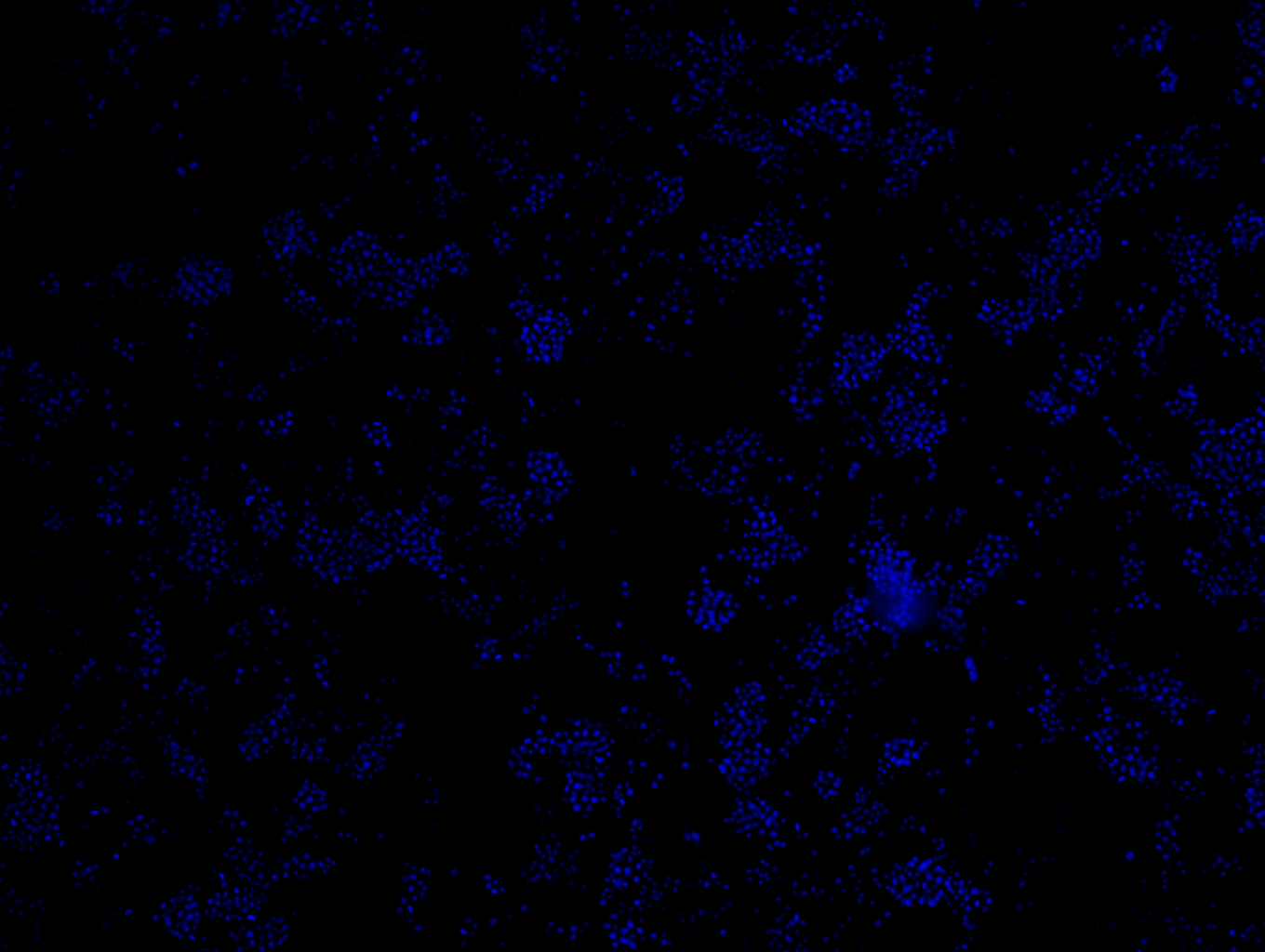

Supplement: Supplementary file 7 [file DataSheet5.ZIP › Immunofluorescence Raw data/C-Blue-2.tif]

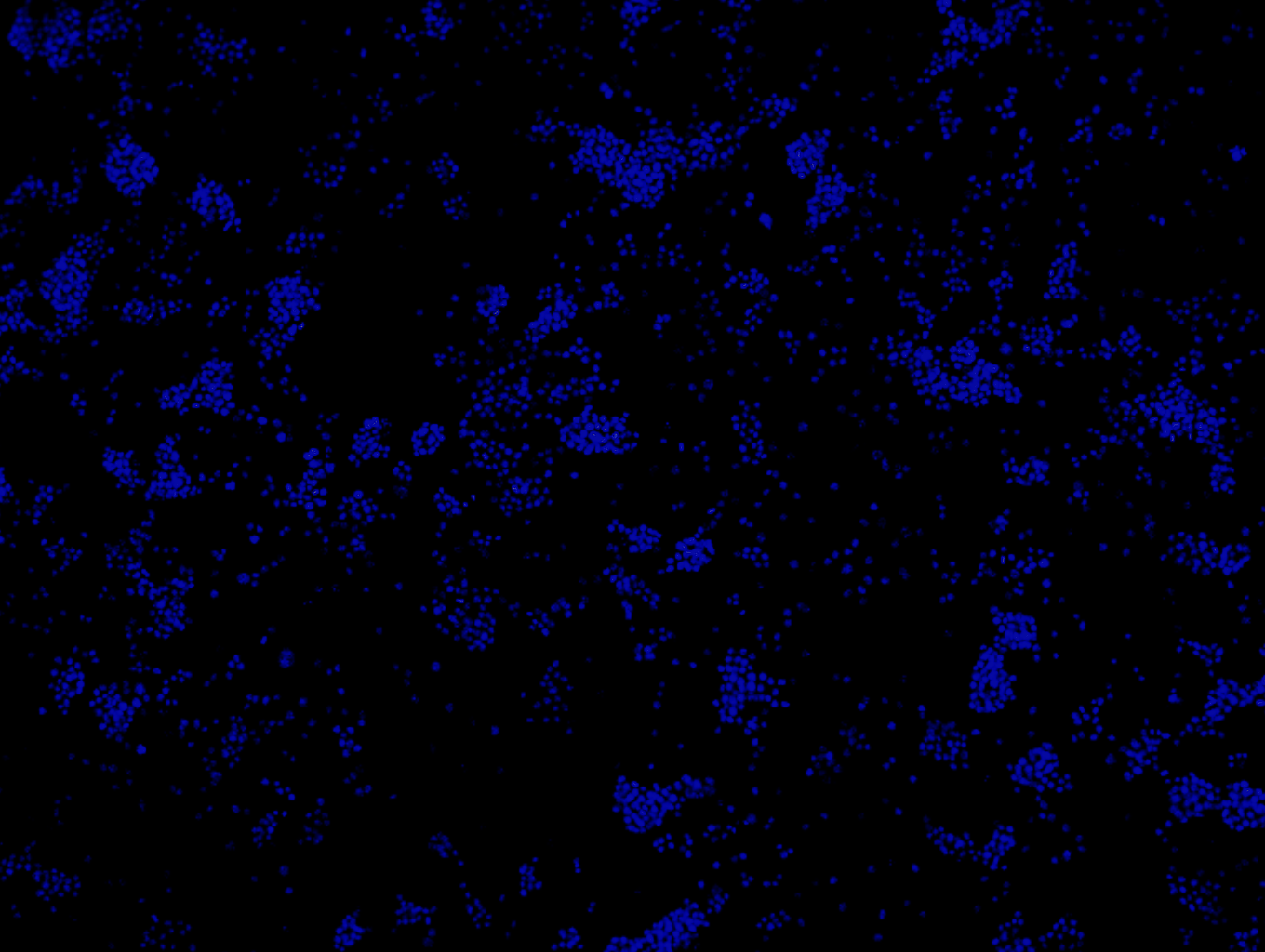

Supplement: Supplementary file 7 [file DataSheet5.ZIP › Immunofluorescence Raw data/C-Blue-3.tif]

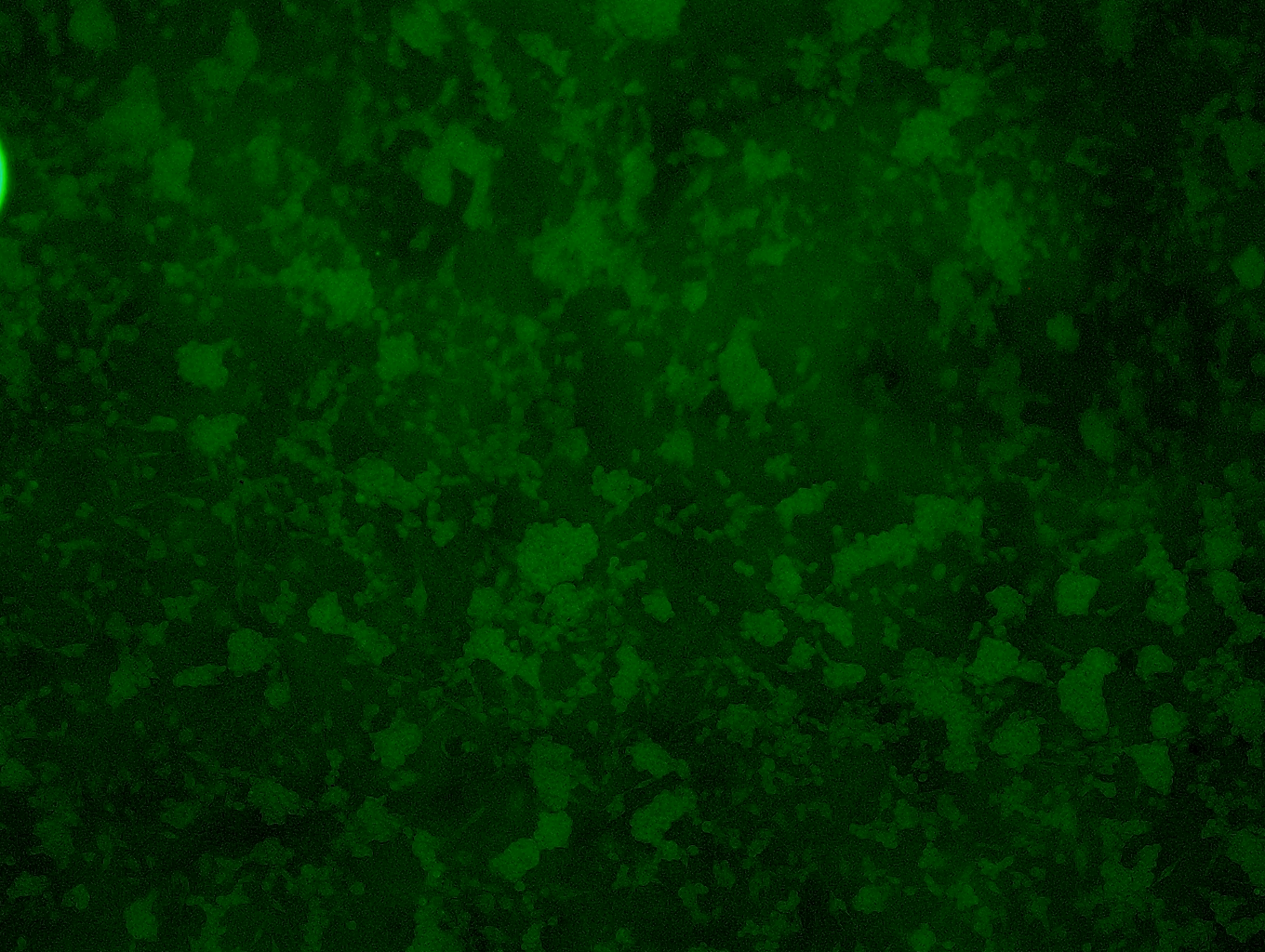

Supplement: Supplementary file 7 [file DataSheet5.ZIP › Immunofluorescence Raw data/C-green-1.tif]

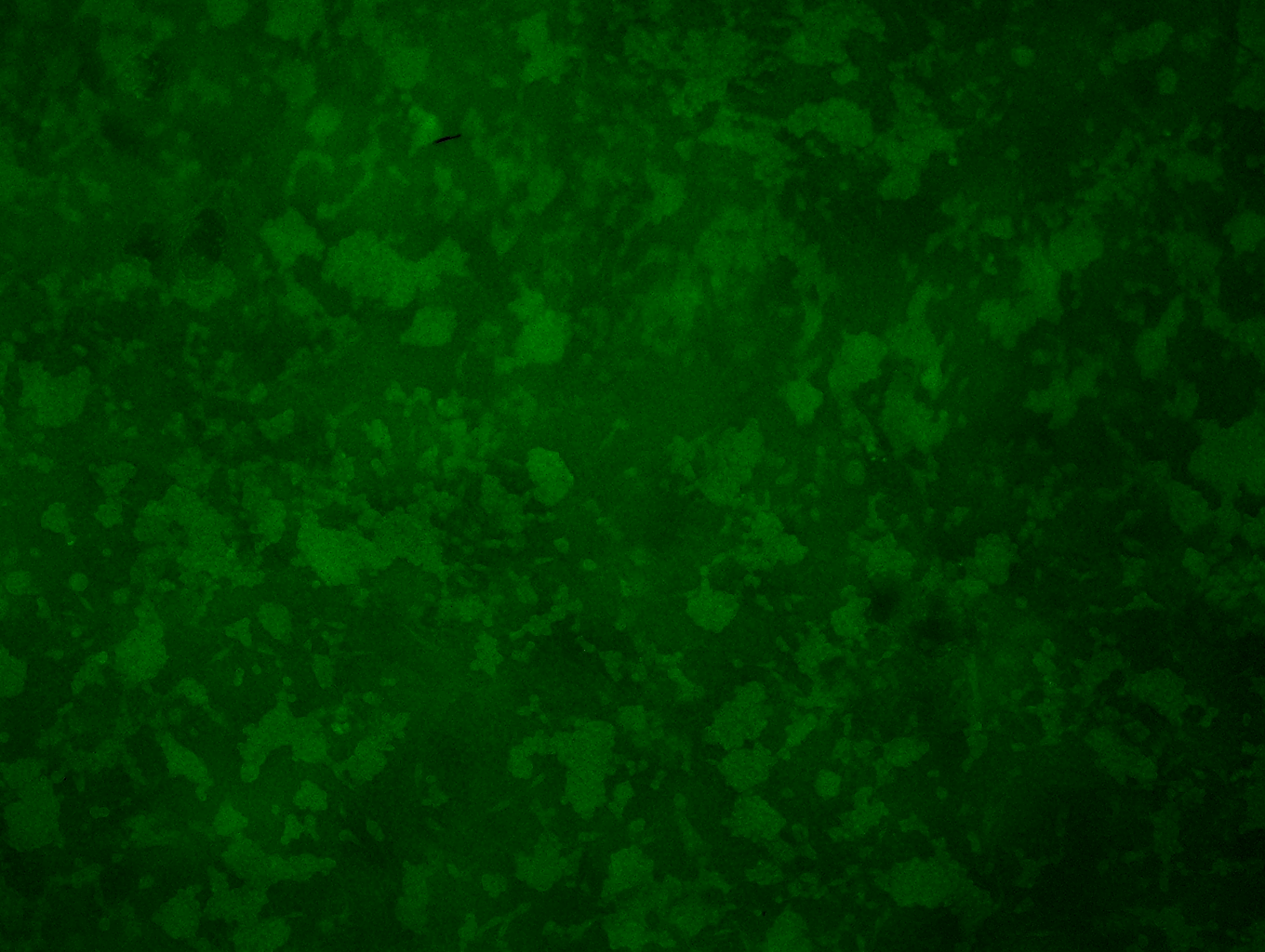

Supplement: Supplementary file 7 [file DataSheet5.ZIP › Immunofluorescence Raw data/C-green-2.tif]

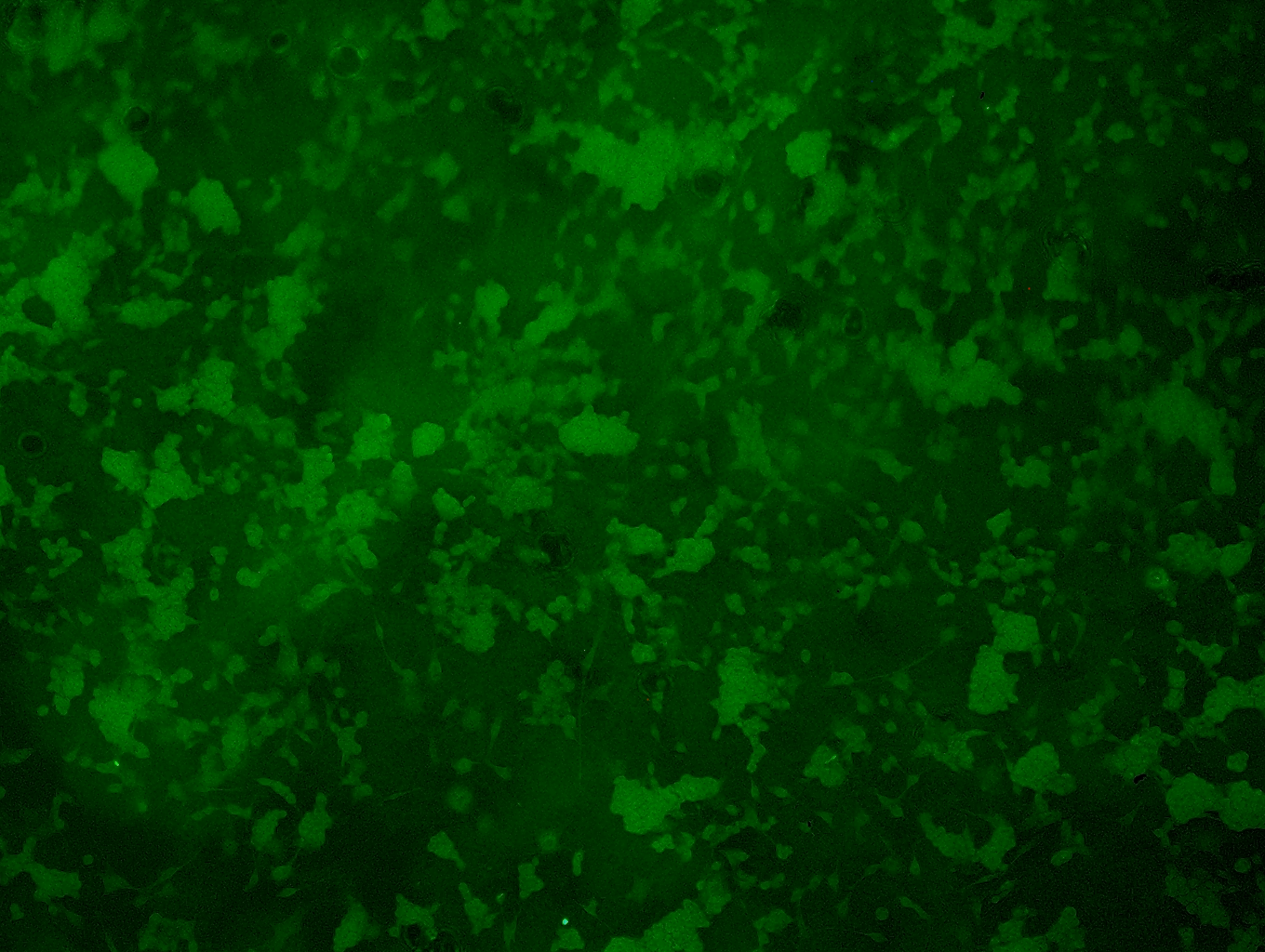

Supplement: Supplementary file 7 [file DataSheet5.ZIP › Immunofluorescence Raw data/C-green-3.tif]

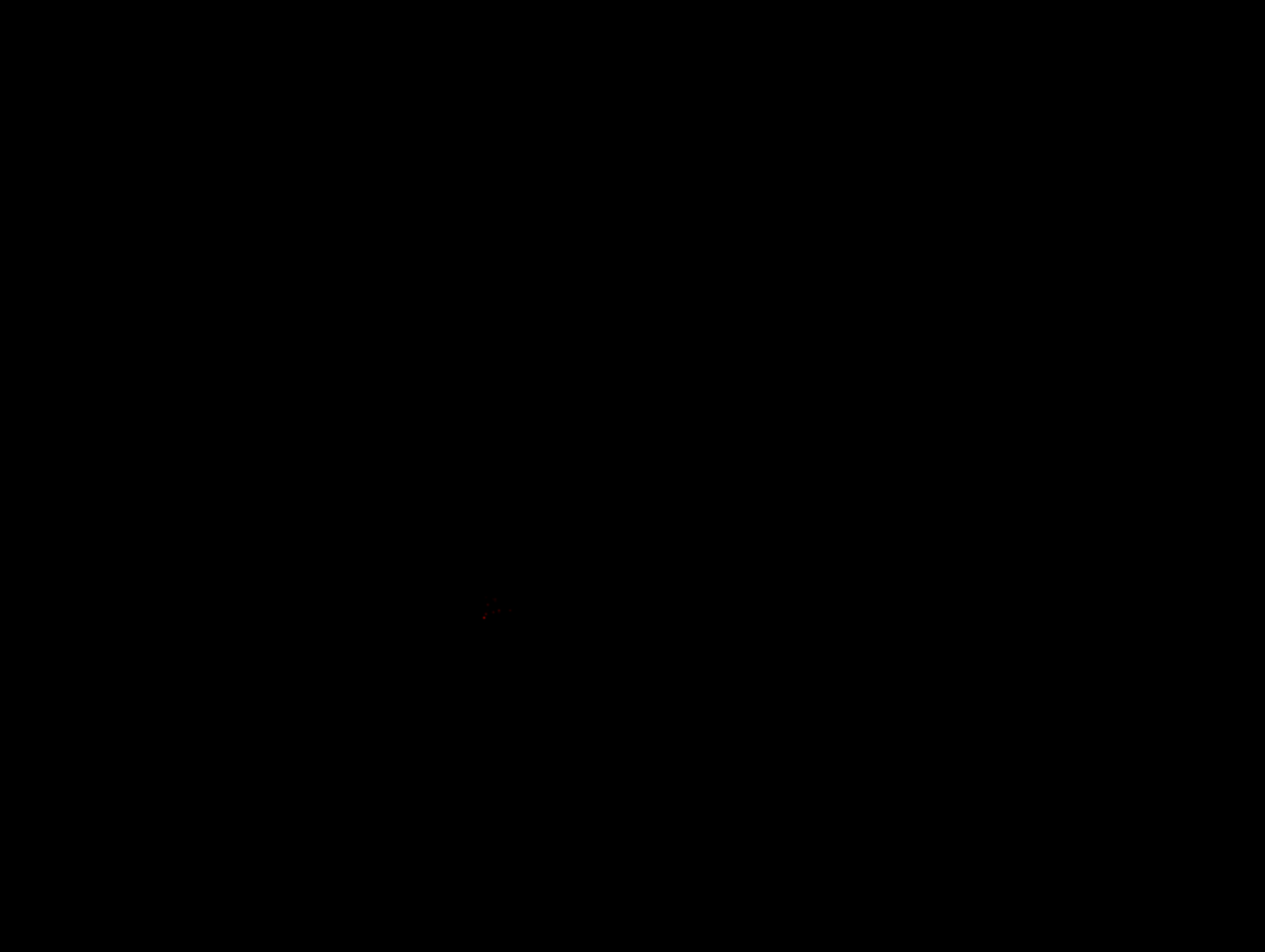

Supplement: Supplementary file 7 [file DataSheet5.ZIP › Immunofluorescence Raw data/C-red-1.tif]

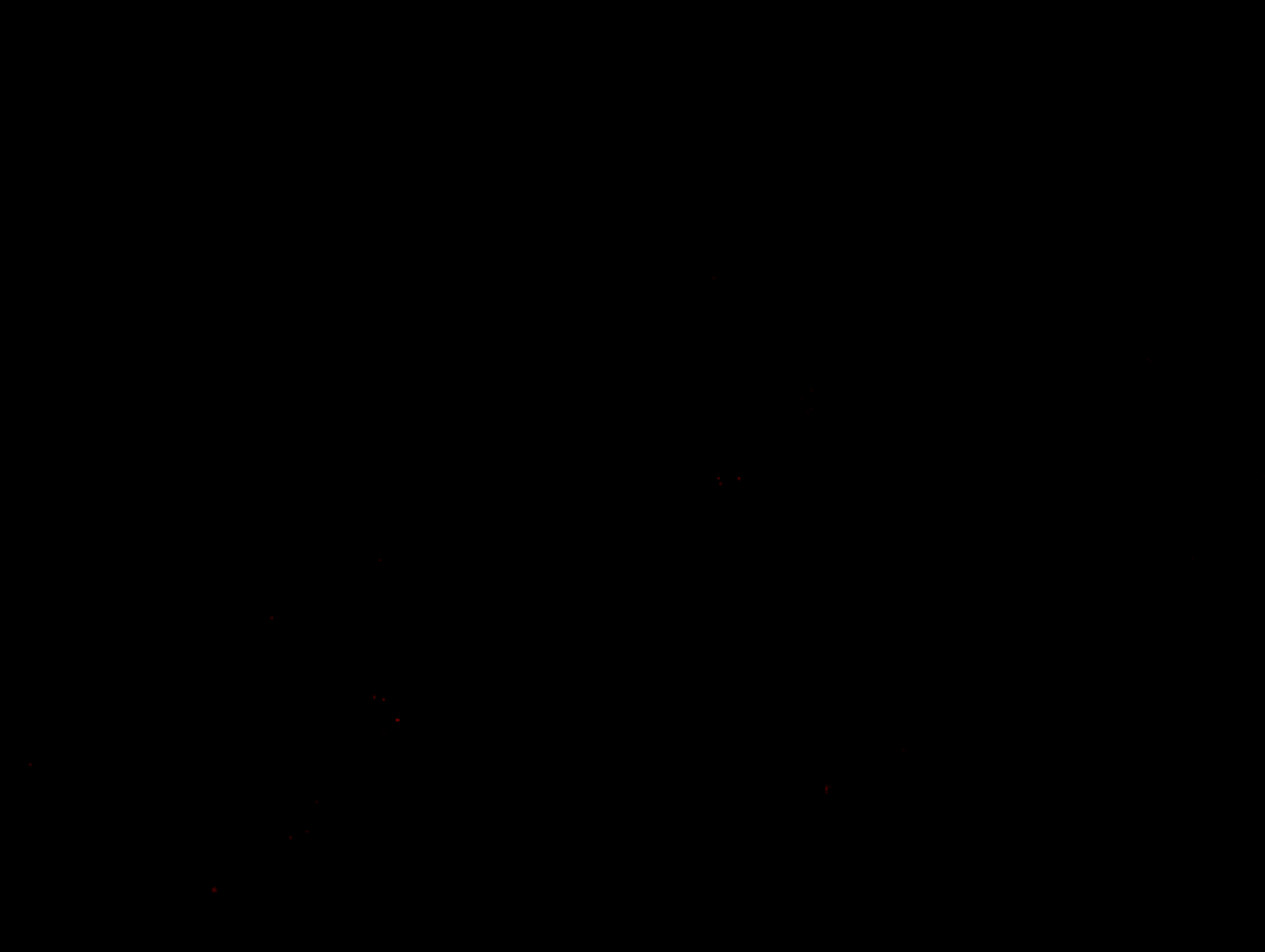

Supplement: Supplementary file 7 [file DataSheet5.ZIP › Immunofluorescence Raw data/C-red-2.tif]

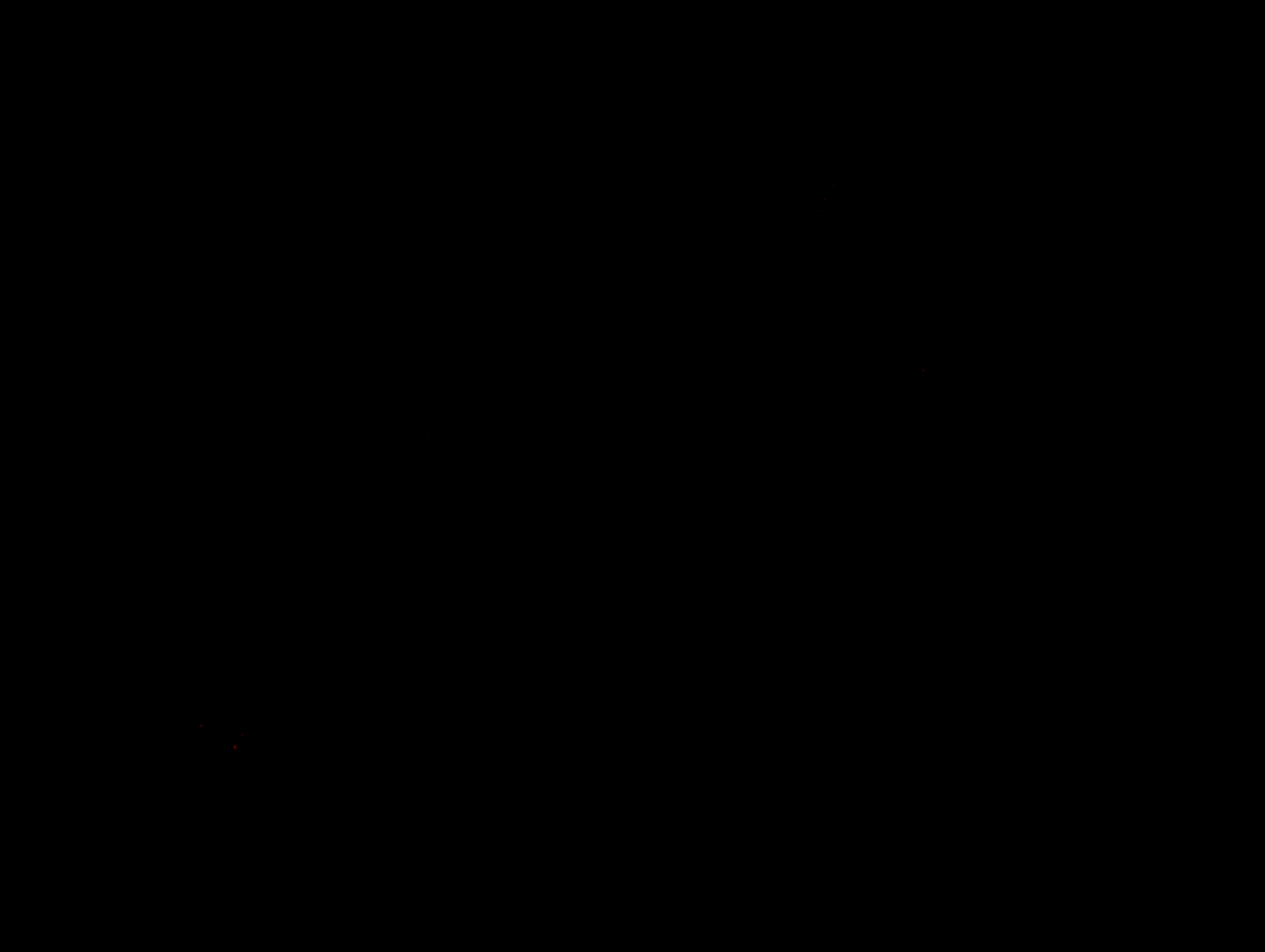

Supplement: Supplementary file 7 [file DataSheet5.ZIP › Immunofluorescence Raw data/C-red-3.tif]

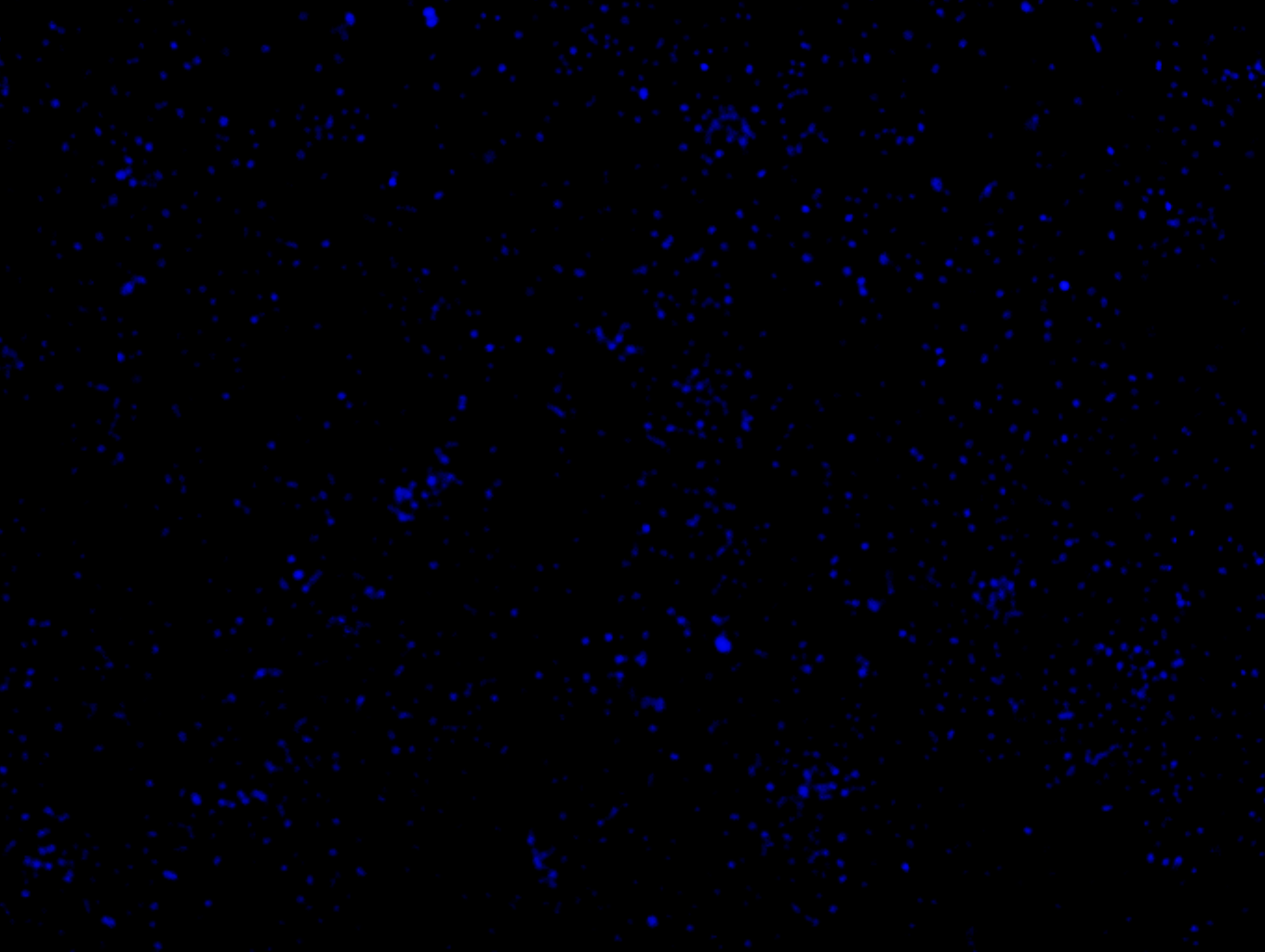

Supplement: Supplementary file 7 [file DataSheet5.ZIP › Immunofluorescence Raw data/LPS-Blue-1.tif]

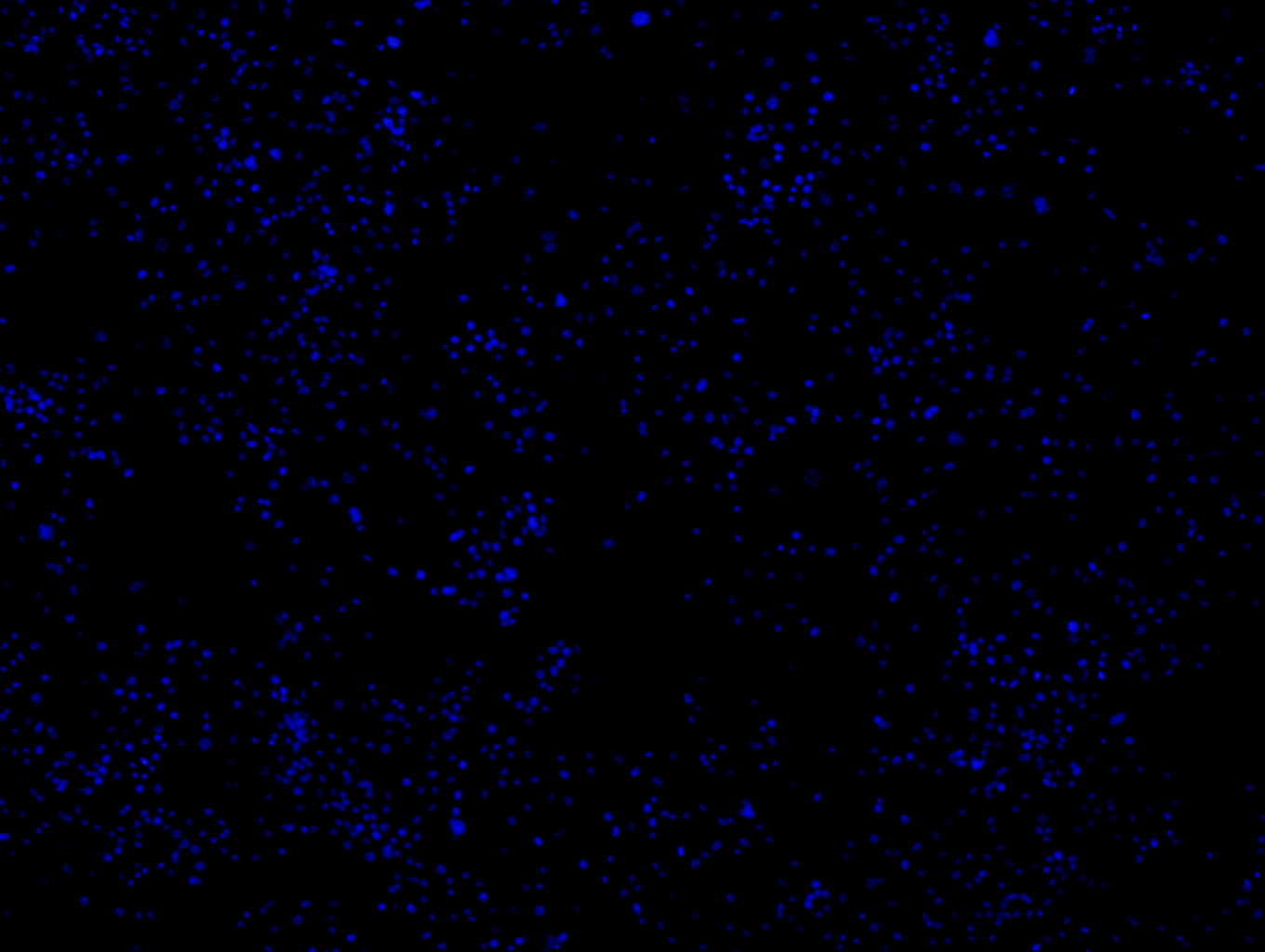

Supplement: Supplementary file 7 [file DataSheet5.ZIP › Immunofluorescence Raw data/LPS-Blue-2.tif]

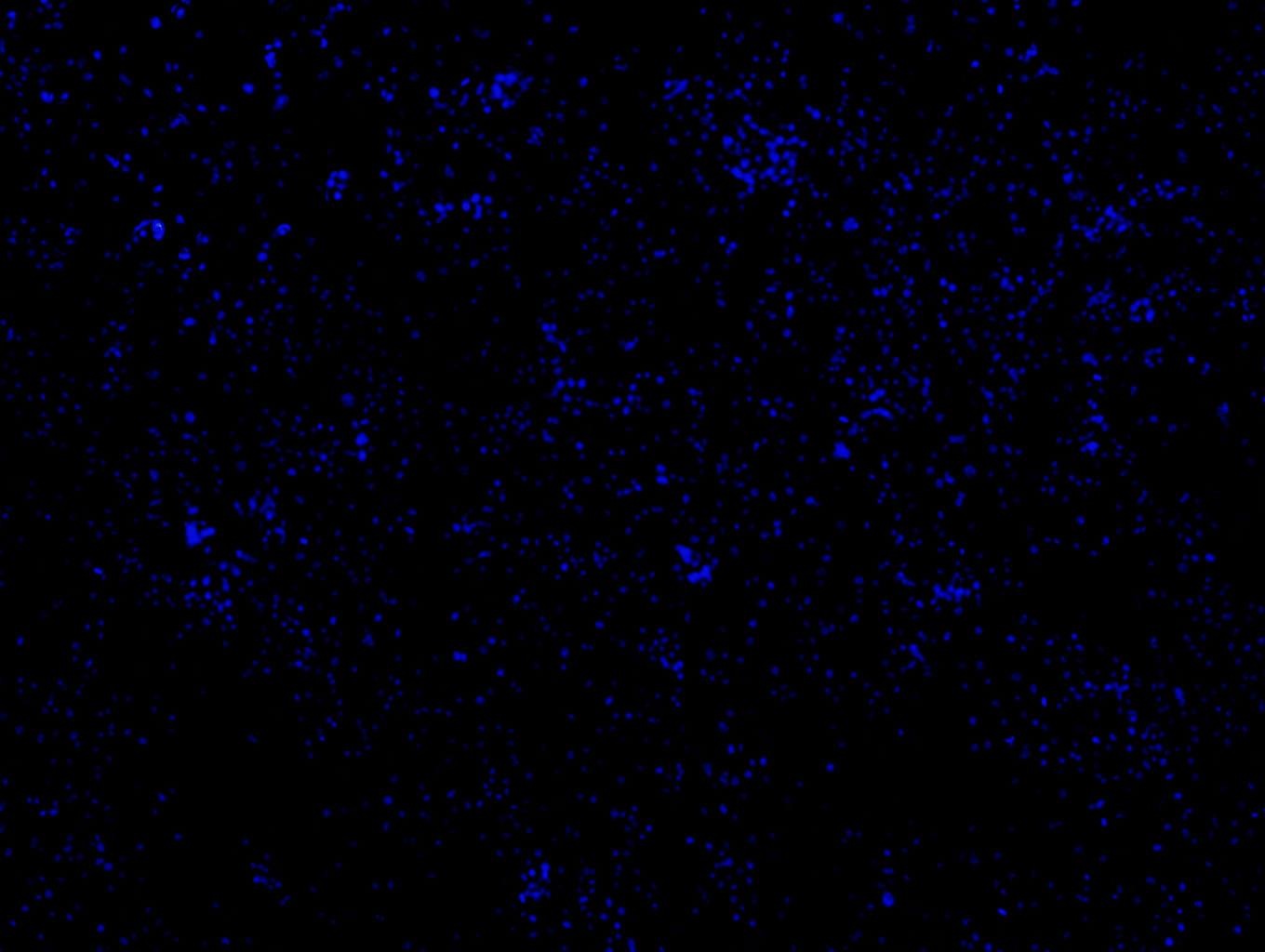

Supplement: Supplementary file 7 [file DataSheet5.ZIP › Immunofluorescence Raw data/LPS-Blue-3.tif]

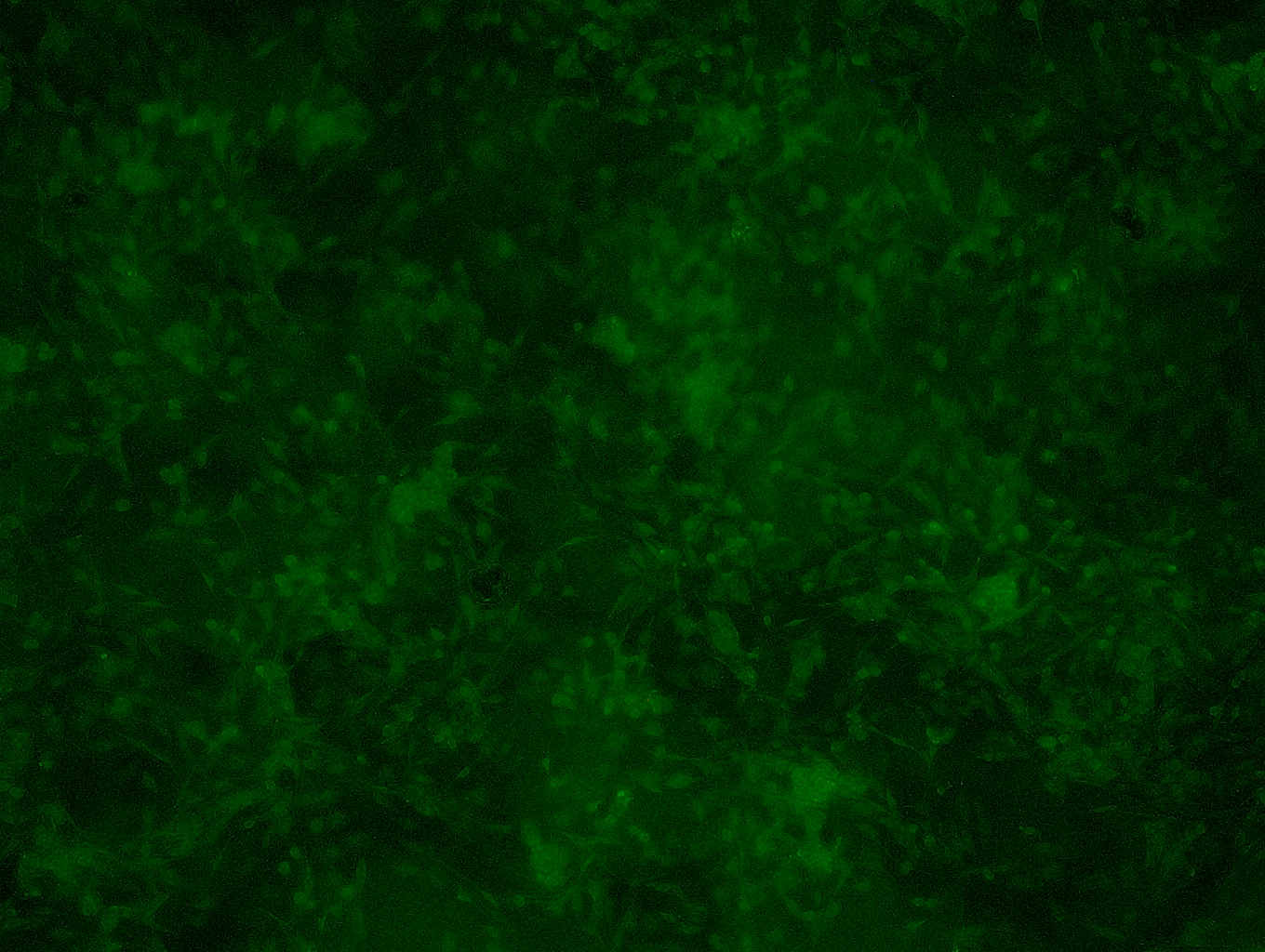

Supplement: Supplementary file 7 [file DataSheet5.ZIP › Immunofluorescence Raw data/LPS-green-1.tif]

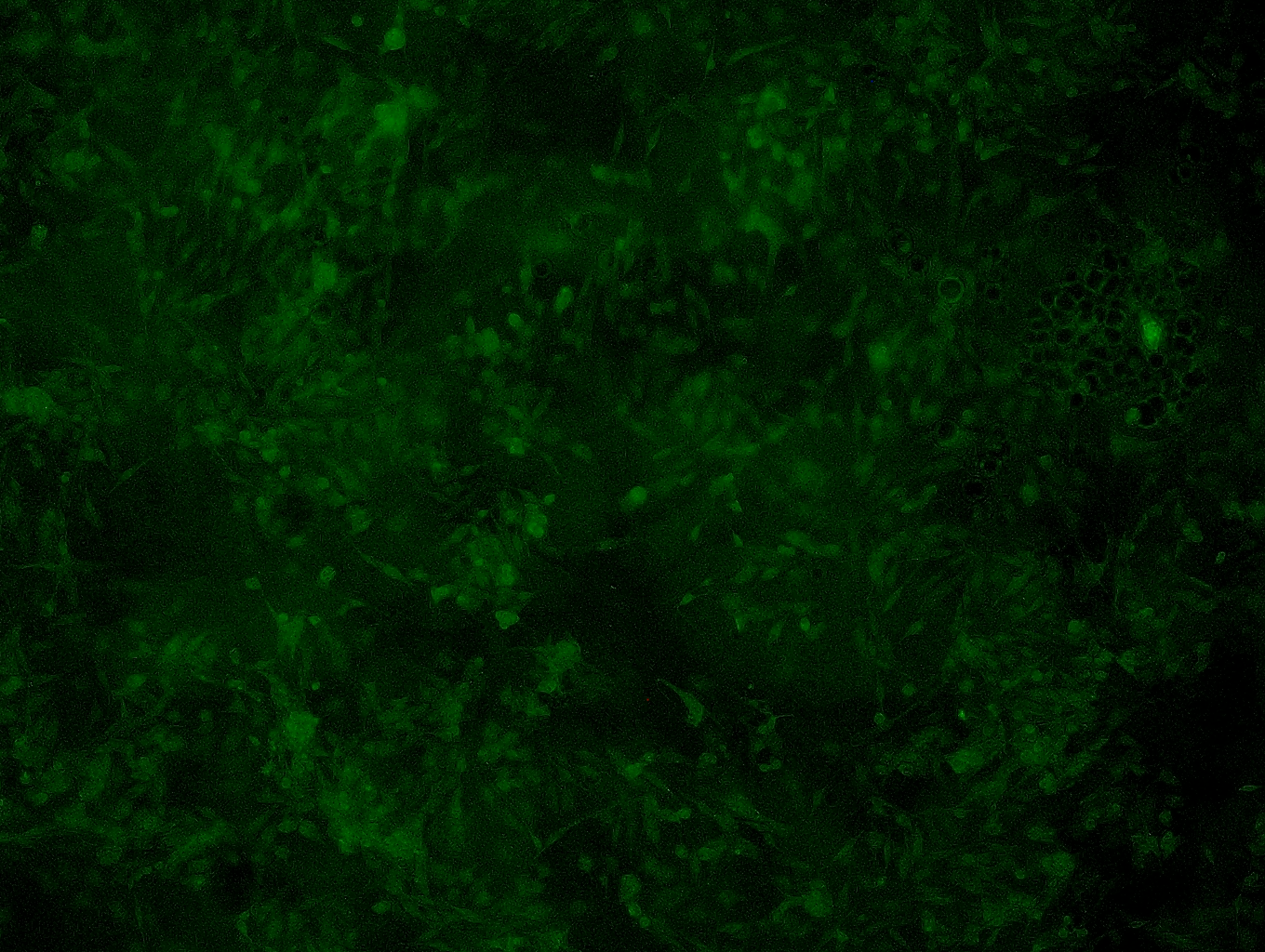

Supplement: Supplementary file 7 [file DataSheet5.ZIP › Immunofluorescence Raw data/LPS-green-2.tif]

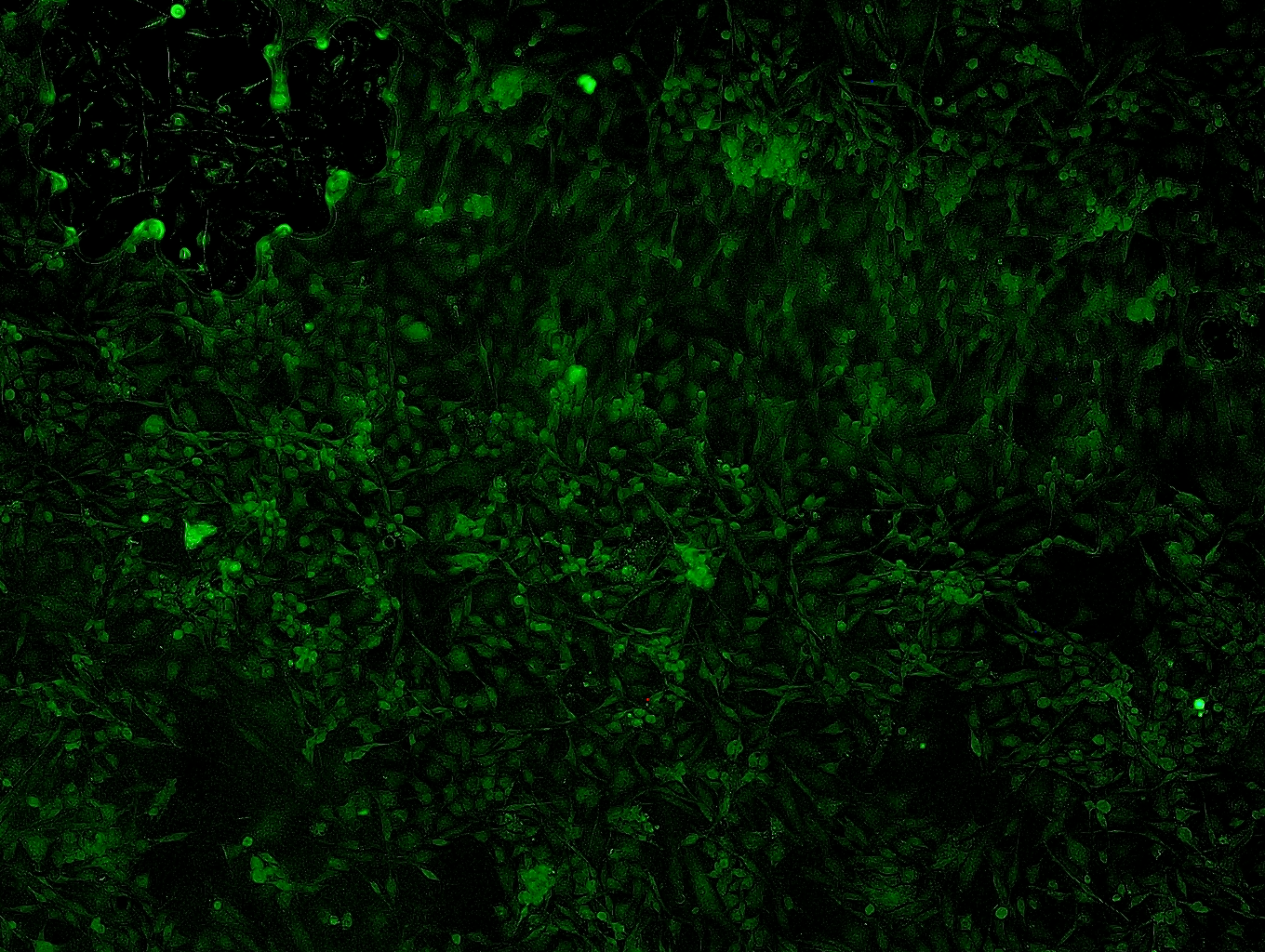

Supplement: Supplementary file 7 [file DataSheet5.ZIP › Immunofluorescence Raw data/LPS-green-3.tif]

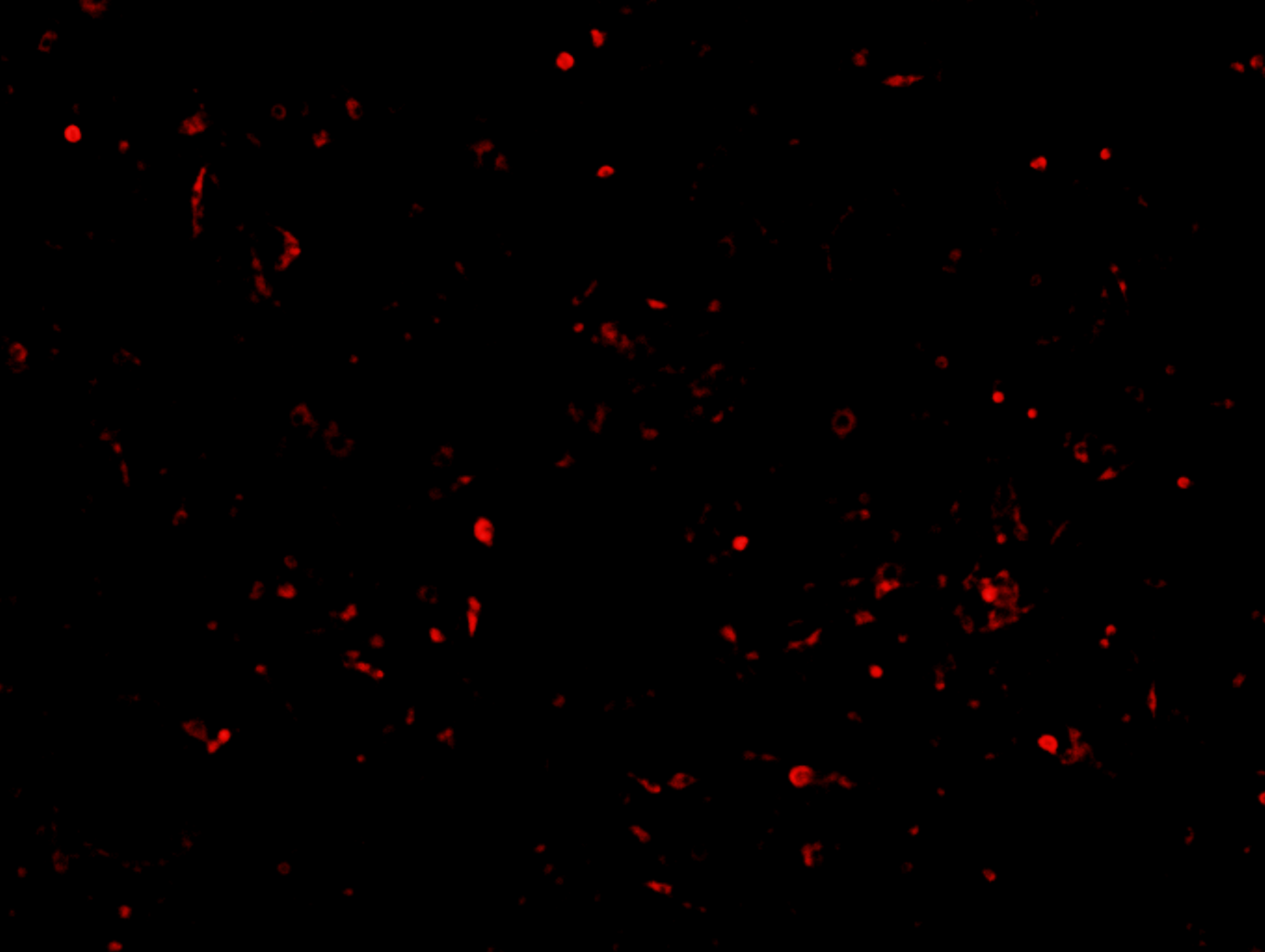

Supplement: Supplementary file 7 [file DataSheet5.ZIP › Immunofluorescence Raw data/LPS-red-1.tif]

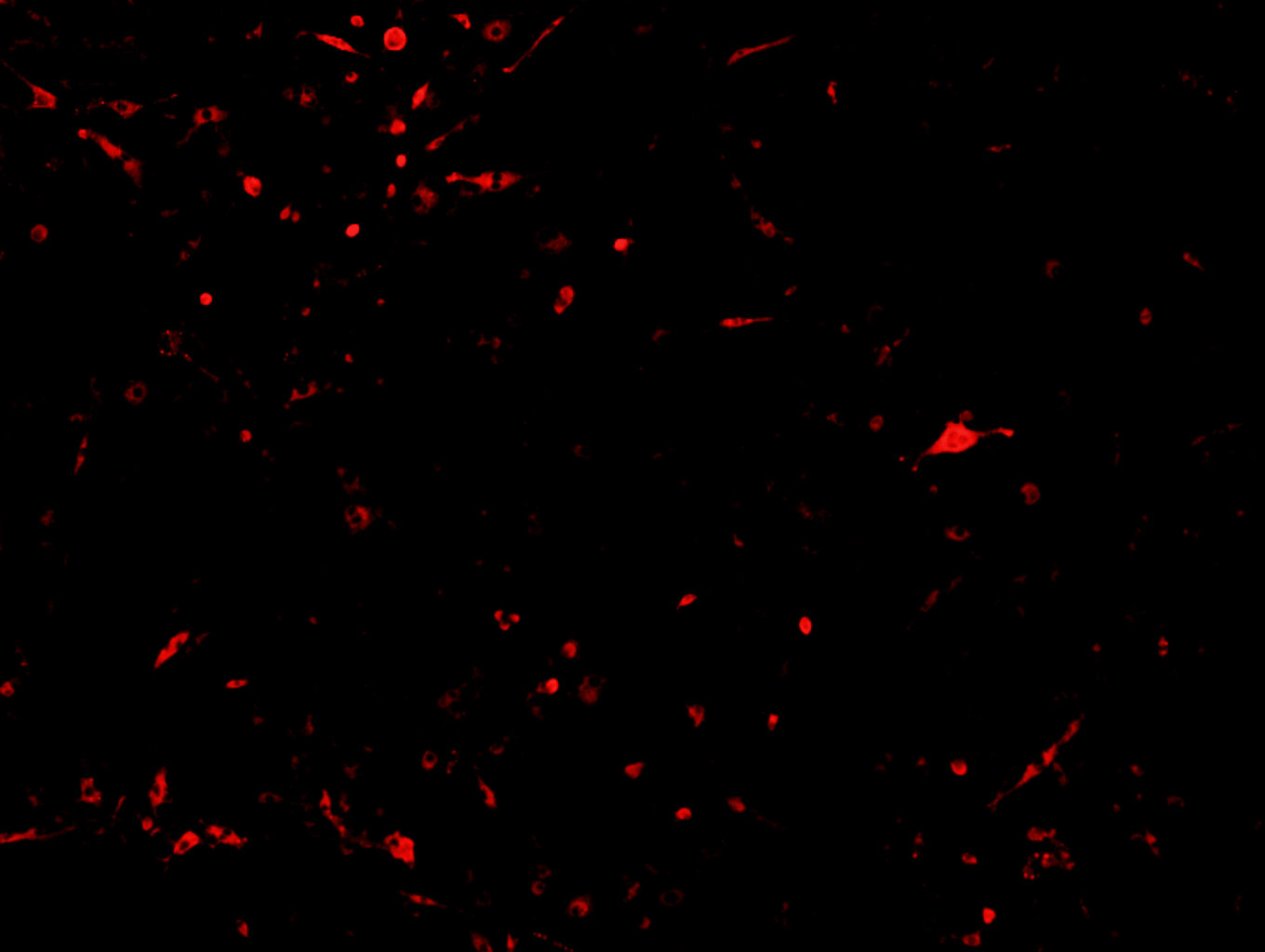

Supplement: Supplementary file 7 [file DataSheet5.ZIP › Immunofluorescence Raw data/LPS-red-2.tif]

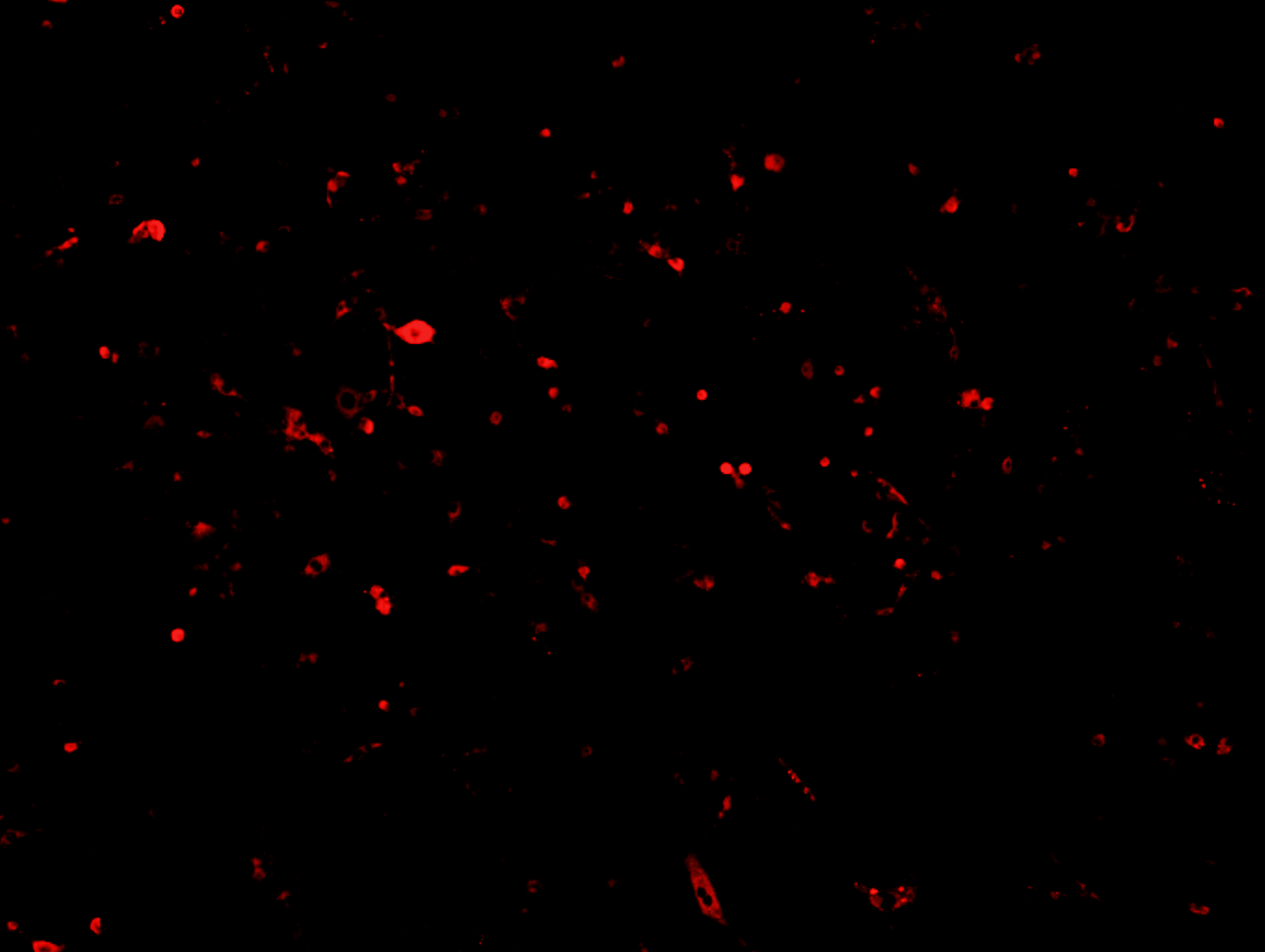

Supplement: Supplementary file 7 [file DataSheet5.ZIP › Immunofluorescence Raw data/LPS-red-3.tif]
