# Supplementary material for: Allyl methyl trisulfide protected against LPS-induced acute lung injury in mice via inhibition of the NF-κB and MAPK pathways
Source: Front Pharmacol. 2022 Aug 8;13:919898. doi: 10.3389/fphar.2022.919898 (PMC9394683; doi:10.3389/fphar.2022.919898)

frontiers

**Supplementary Figure 3.** Western blot bands.

**pIKBα**

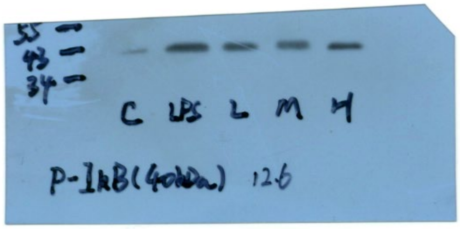


**Nuclear NF-κB p65**

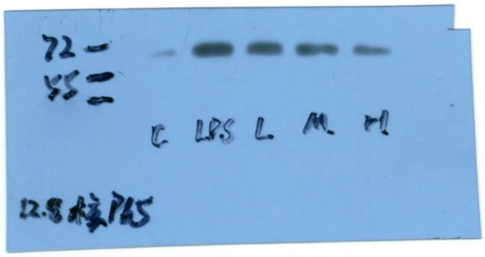


**IKBα**

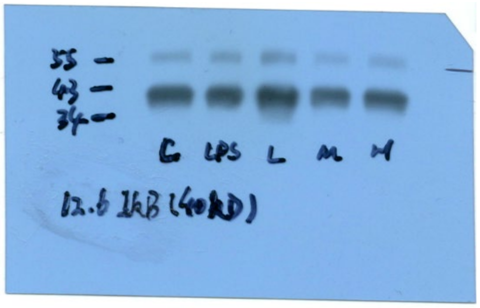


**Lamin B**

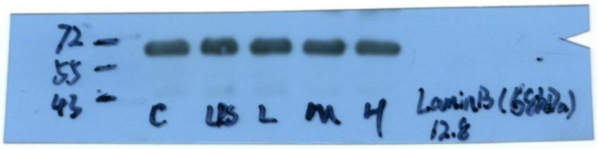


**pERK**

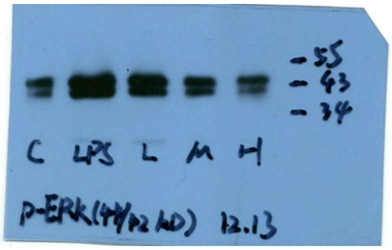


**Cytoplasm NF-κB p65**

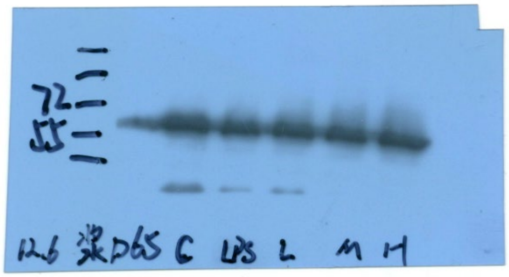


**ERK**

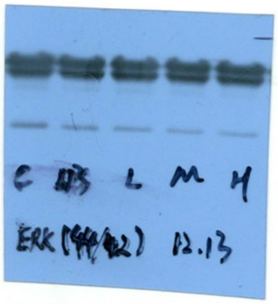


**β-actin**

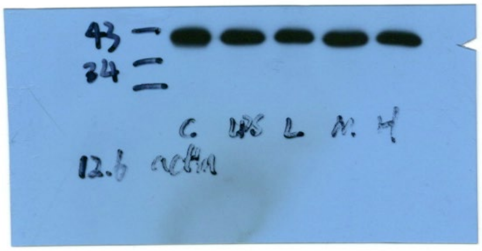

**pSAPK/JNK**

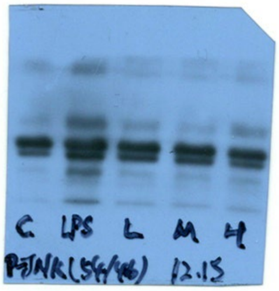


**phospho-p38 MAPK**

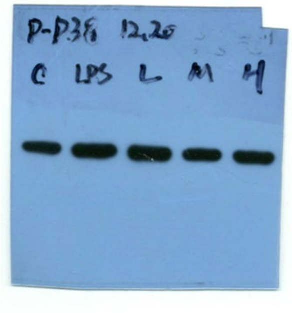


**SAPK/JNK**

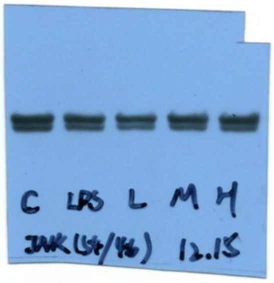


**p38 MAPK**

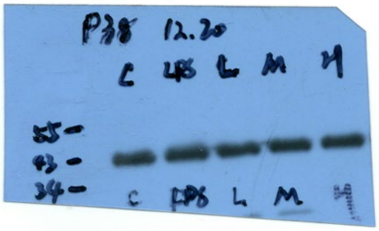

Supplement: Supplementary file 5 [file Image3.pdf]
