# Supplementary material for: Allyl methyl trisulfide protected against LPS-induced acute lung injury in mice via inhibition of the NF-κB and MAPK pathways
Source: Front Pharmacol. 2022 Aug 8;13:919898. doi: 10.3389/fphar.2022.919898 (PMC9394683; doi:10.3389/fphar.2022.919898)
Supplement: Supplementary file 6 [file DataSheet2.DOCX]

**Supplementary Figure 1.** H&E staining of lung tissue.

| **Control Group** | | |
| --- | --- | --- |
| 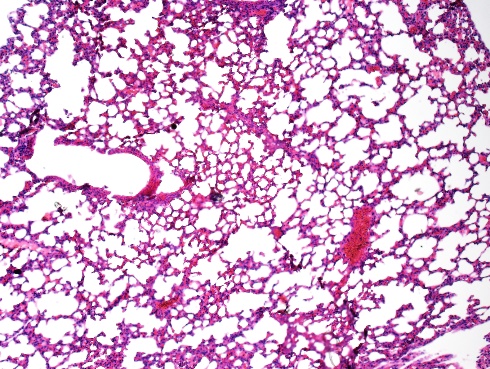 | 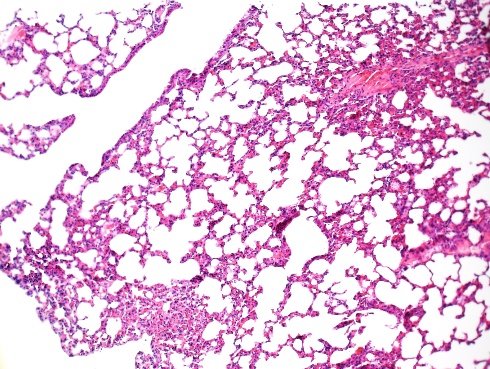 | 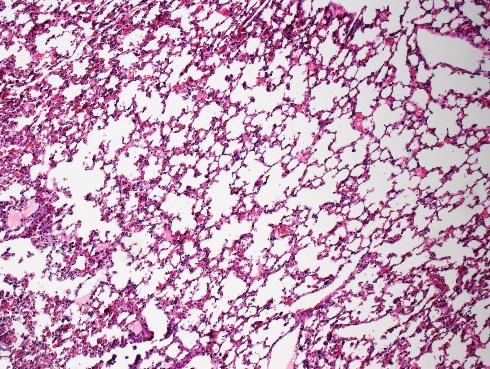 |
| **LPS** | | |
| 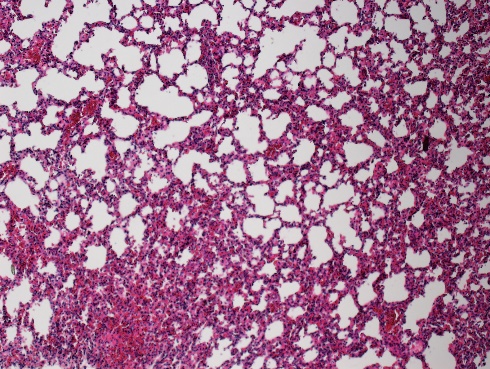 | 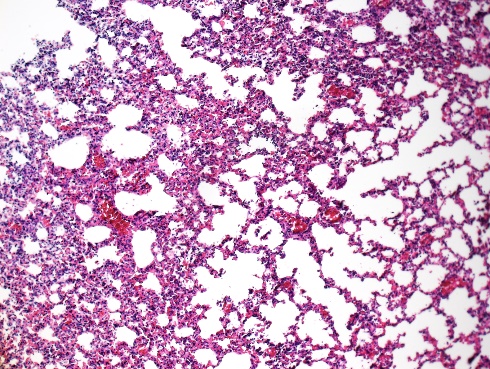 | 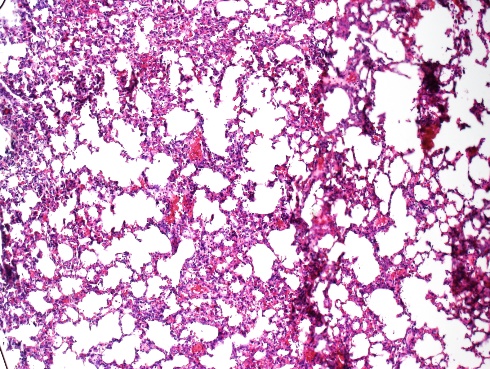 |
| **LPS+AMTS 25 mg/kg** | | |
| 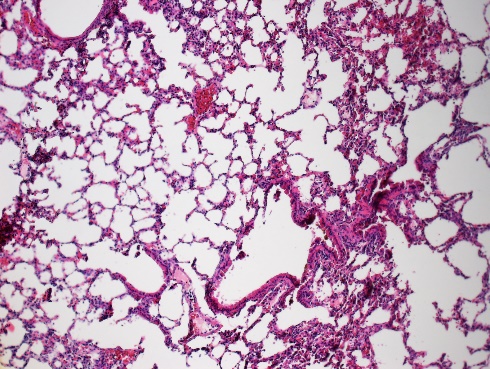 | 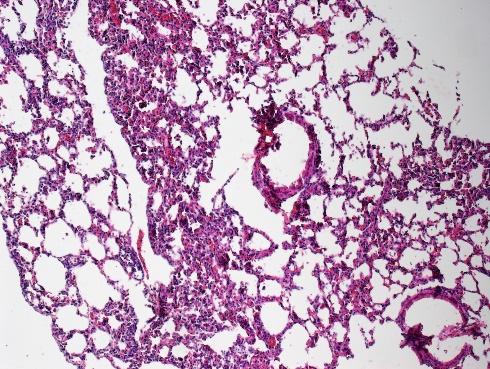 | 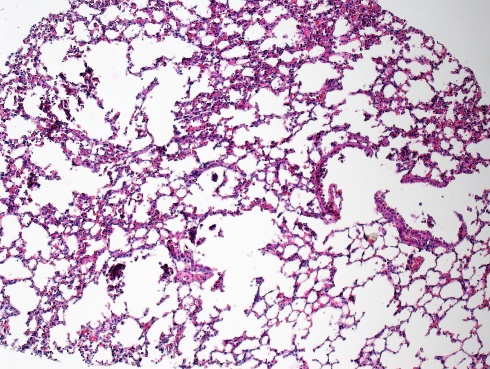 |
| **LPS+AMTS 50 mg/kg** | | |
| 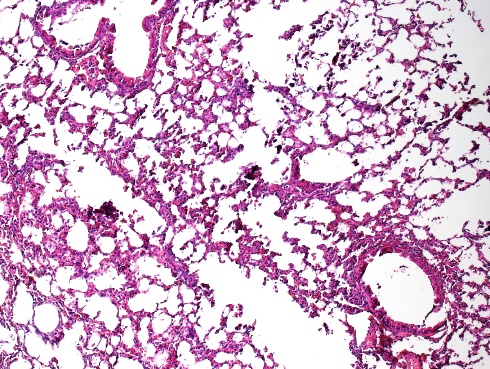 | 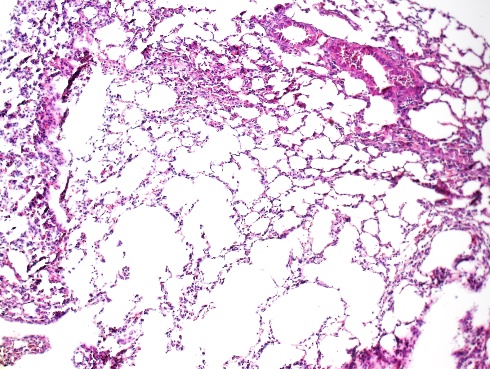 | 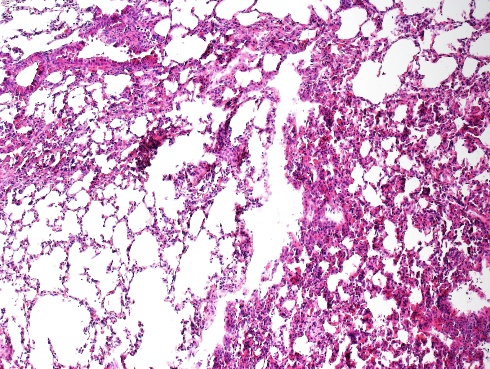 |
| **LPS+AMTS 100 mg/kg** | | |
| 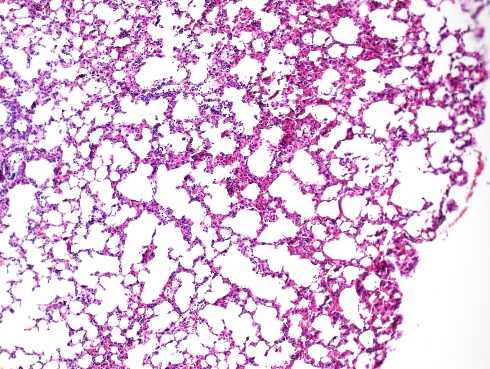 | 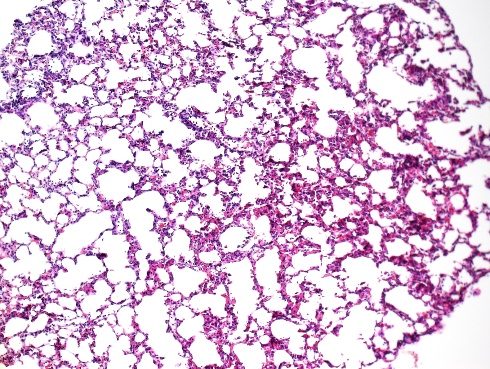 | 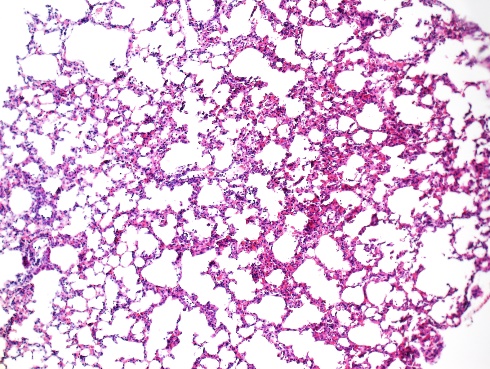 |
