# Supplementary material for: Allyl methyl trisulfide protected against LPS-induced acute lung injury in mice via inhibition of the NF-κB and MAPK pathways
Source: Front Pharmacol. 2022 Aug 8;13:919898. doi: 10.3389/fphar.2022.919898 (PMC9394683; doi:10.3389/fphar.2022.919898)

Supplementary Figure 1. H&E staining of lung tissue.

Control Group

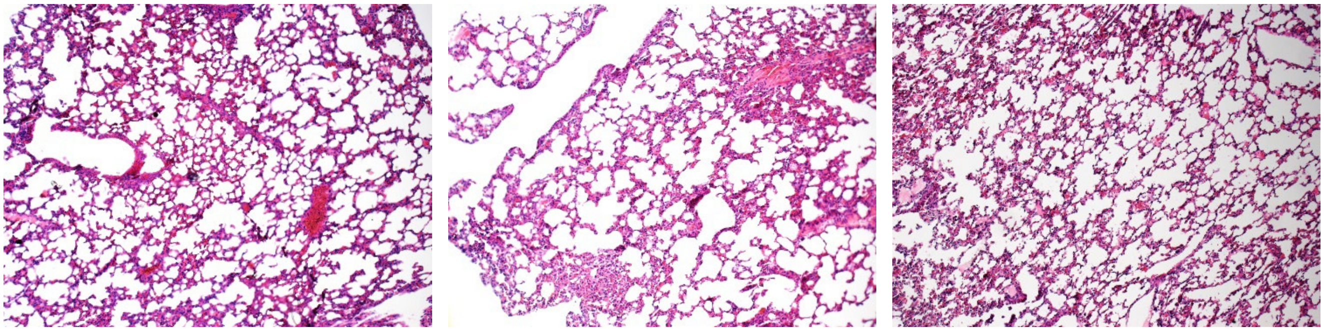

LPS

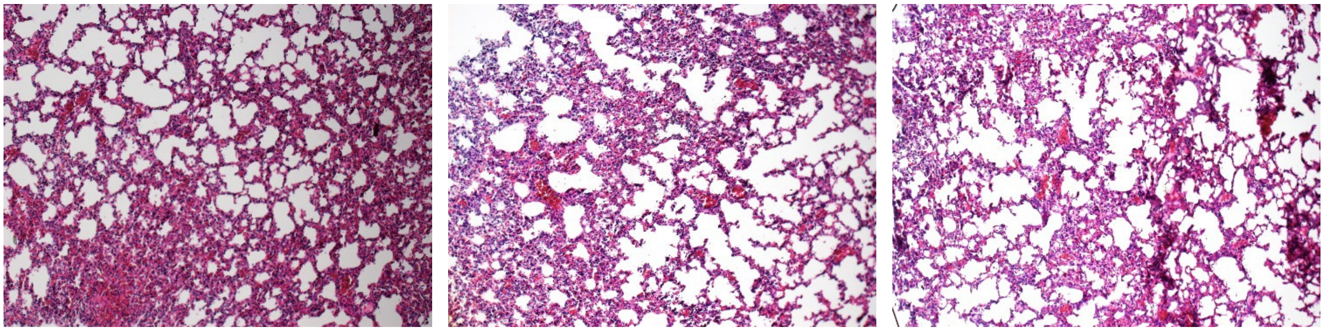

LPS+AMTS 25 mg/kg

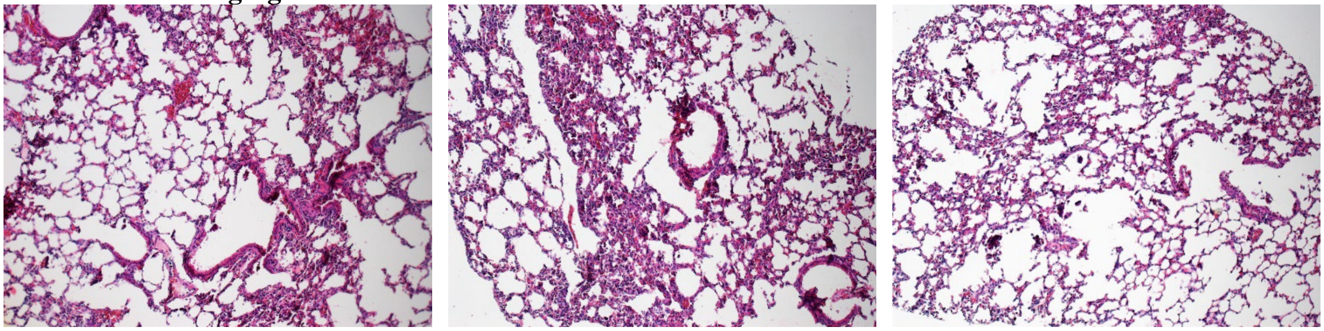

LPS+AMTS 50 mg/kg

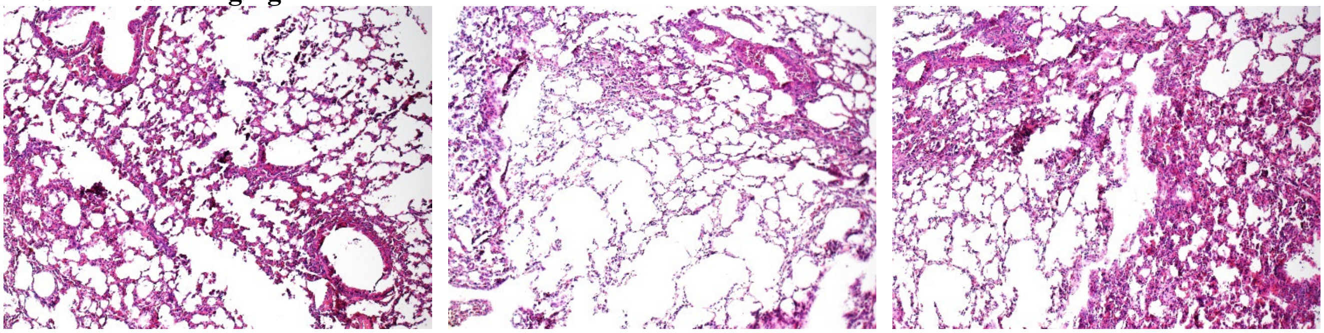

LPS+AMTS 100 mg/kg

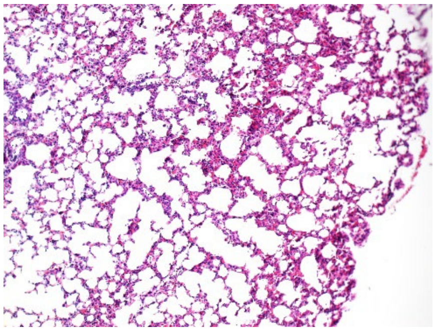
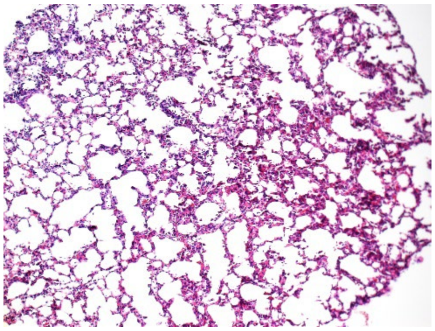
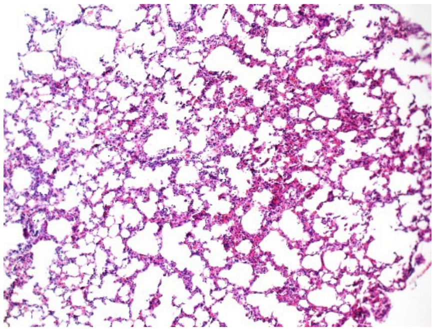

Supplement: Supplementary file 8 [file Image1.pdf]
